# Phenol Derivatives From the Sponge-Derived Fungus *Didymellaceae* sp. SCSIO F46

**DOI:** 10.3389/fchem.2018.00536

**Published:** 2018-11-01

**Authors:** Yongqi Tian, Xiuping Lin, Xuefeng Zhou, Yonghong Liu

**Affiliations:** ^1^College of Biological Science and Technology, Fuzhou University, Fuzhou, China; ^2^CAS Key Laboratory of Tropical Marine Bio-resources and Ecology, Guangdong Key Laboratory of Marine Materia Medica, South China Sea Institute of Oceanology, Chinese Academy of Sciences, Guangzhou, China; ^3^University of Chinese Academy of Sciences, Beijing, China

**Keywords:** sponge-derived fungus, *Didymellaceae* sp., Phenol derivatives, cytotoxic, COX-2 inhibitory

## Abstract

Seven new phenol derivatives named coleophomones E and F (**1**, **2**), diorcinols L and M (**3**, **4**), 1-hydroxy-6-methyl-11-methoxy-8-hydroxymethylxanthone (**5**), porric acid E (**6**), and 7-(2-hydroxyphenyl) butane-7,8,9-triol (**7**), were isolated from the EtOAc extract of the marine sponge-derived fungus *Didymellaceae* sp. SCSIO F46, together with 10 known compounds. Their structures were determined by spectroscopic analyses, including NMR, MS, X-ray diffraction, and theoretical calculations. Each of **1** and **2** contains an unusual spiro [cyclohexane-1,2′-inden] moiety, which is relatively seldom in nature products. Cytotoxic and COX-2 inhibitory activities of all purified compounds were tested and evaluated. Compound **3** displayed obvious cytotoxicities against Huh-7, HeLa, DU145 and HL60 cells (IC_50_ values 5.7–9.6 μM) and weak activities against other five cell lines, while **8** showed weak cytotoxicities against HeLa and HL7702 cells. Compound **6** displayed COX-2 inhibitory activity with IC_50_ value of 3.3 μM.

## Introduction

Natural products are still irreplaceable and continuing sources of novel drug leads, especially in the anti-infective area (Newman and Cragg, 2016). The marine ecosystem is one of the most complex and largest aquatic systems on earth, and host a huge microbial biodiversity (Agrawal et al., 2017; Corinaldesi et al., 2017). The unique and extreme characteristics of marine systems have driven a variety of biological adaptations, leading to the production of a large number of novel molecules for the treatment of many diseases (Gerwick and Fenner, 2013; Blunt et al., 2017). Marine sponges, a kind of precious marine organisms for new drug discovery, are hosts for a large community of microbes (up to 50–60% of the biomass of the sponge host) (Bergmann and Burke, 1955; Wang, 2006; Zhang et al., 2017). It was indicated that the symbiotic microbes of marine sponges might be the true producers of bioactive chemical defense substance of the sponge ecosystem (Richelle-Maurer et al., 2003; Thomas et al., 2010).

**Figure 1:**
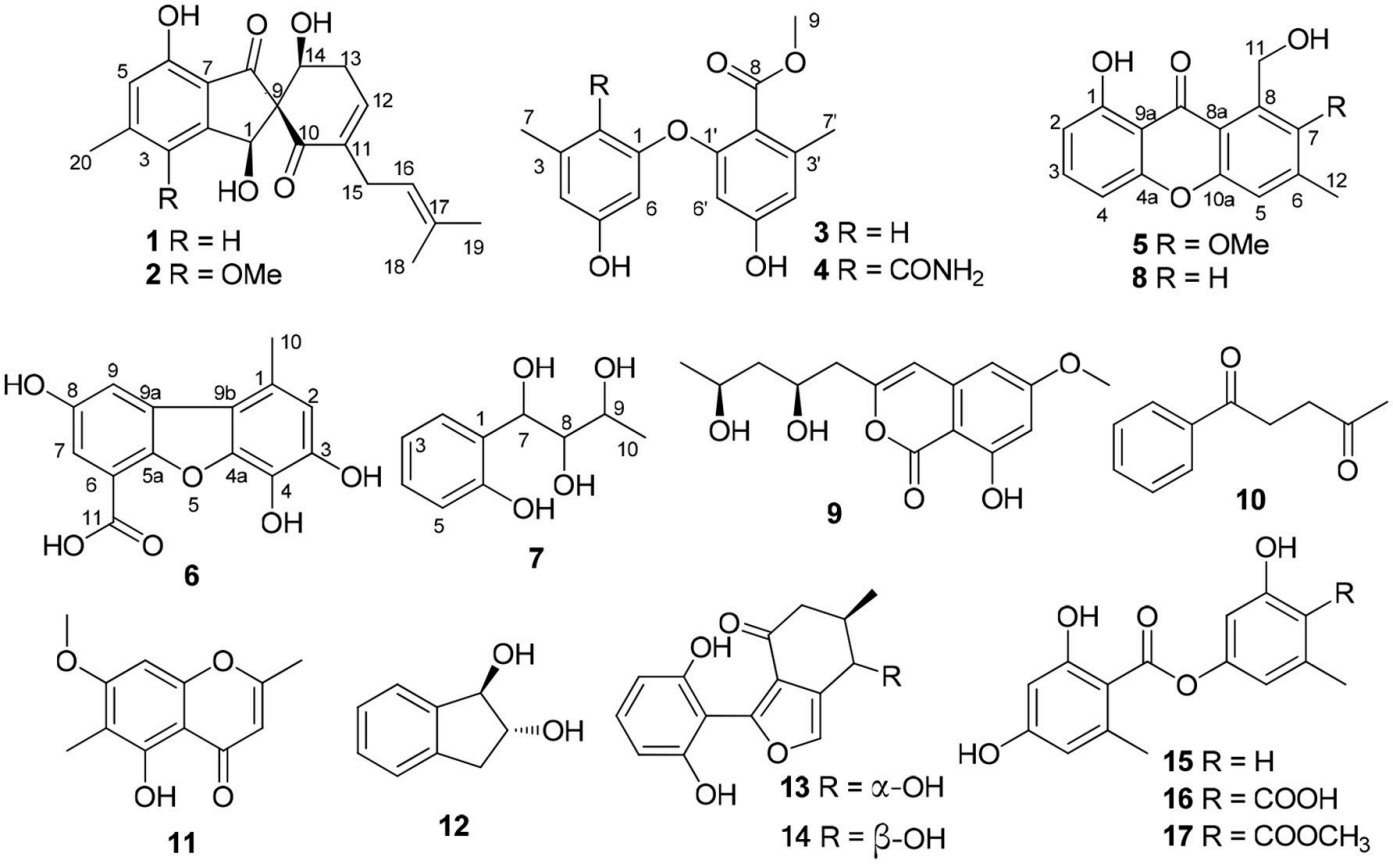
Structures of **1**–**17**.

Sponge-derived fungi have been proven to be a treasure trove of novel biomolecules (Indraningrat et al., 2016; Blunt et al., 2017). During an ongoing search for new bioactive metabolites from the sponge-derived fungi (Tian et al., 2015a, 2018a,b), a strain of *Didymellaceae* sp. (SCSIO F46) isolated from a sponge *Callyspongia* sp. was subjected to chemical study. The EtOAc extract of rice fermentation of F46 showed toxicity against brine shrimp. Further isolation yielded seven new phenol derivatives, coleophomones E, F (**1**, **2**), diorcinols L, M (**3,4**), 1-hydroxy-6-methyl-11-methoxy-8-hydroxymethylxanthone (**5**), porric acid E (**6**), and 7-(2-hydroxyphenyl) butane-7,8,9-triol (**7**), together with ten known compounds (Figure 1). The cytotoxic and COX-2 inhibitory activities of all compounds were evaluated. Details of the isolation structure elucidation, and bioactivity screening of these metabolites are reported herein.

**Figure 2:**
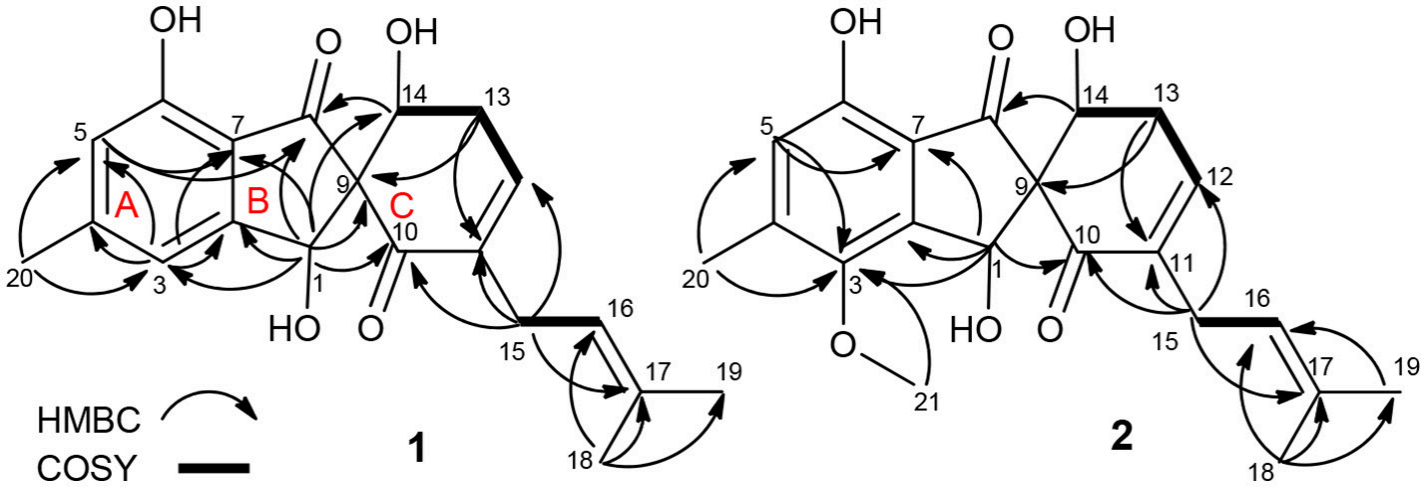
Key COSY and HMBC correlations of **1** and **2**.

## Materials and methods

### General experimental procedures

The NMR spectra were recorded on a Bruker AC 500 NMR (Bruker, Fällanden, Switzerland) spectrometer with TMS as an internal standard. HRESIMS data were measured on a Bruker micro TOF-QII mass spectrometer (Bruker, Fällanden, Switzerland). UV spectra were recorded on a Shimadzu UV-2600 UV-Vis spectrophotometer (Shimadzu, Kyoto, Japan). ECD spectra were performed on a Chirascan circular dichroism spectrometer (Applied Photophysics). X-ray diffraction intensity data were collected on a CrysAlis PRO charge-coupled device (CCD) area detector diffractometer with graphite monochromated Cu Kα radiation (λ = 1.54178 Å). Semi-preparative reversed-phase HPLC (RP-HPLC) was performed on a YMC-Pack Pro C_18_ *RS* column (5 μm, 250 × 10 mm id; YMC, Kyoto, Japan) with a Agilent 1260 separation module equipped with a Photodiode Array (PDA) detector. Silica gel GF254 used for TLC were supplied by the Qingdao Marine Chemical Factory, Qingdao, China. Sephadex LH-20 gel (GE Healthcare, Uppsala, Sweden) was used. Spots were detected on TLC under UV light or by heating by spraying with 12% H_2_SO_4_ in H_2_O.

### Fungal material

The fungal strain SCSIO F46 was isolated from a sponge *Callyspongia* sp., collected from the sea area near Xuwen County, Guangdong Province, China, during August 2013. The isolate was stored on MB agar (malt extract 15 g, sea salt 10 g, agar 15 g) slants at 4°C and then deposited at CAS Key Laboratory of Tropical Marine Bio-resources and Ecology. The fungus was identified using a molecular biological protocol by DNA amplification and sequencing of the ITS region. The nucleotide sequence of the ITS region reported in this article was assigned the GenBank accession number KU361223.

### Extraction and isolation

*Didymellaceae* sp. SCSIO F46 was cultured on MB-agar plates at 25°C for 7 days. The seed medium (malt extract 15 g, sea salt 10 g, distilled water 1,000 mL, pH 7.4–7.8) was inoculated with strain F46 and incubated at 25°C for 72 h on a rotating shaker (170 rpm). Mass scale fermentation of F46 was carried out using solid rice medium in 1,000 mL flasks (rice 200 g, sea salt 2.5 g, distilled water 200 mL), and inoculated with 10 mL of seed solution. Flasks were incubated at 25°C under normal day night cycle. After 30 days, cultures from 30 flasks were harvested. The culture of solid rice medium was soaked in acetone and cut into small pieces and kept for 1 day. The content was filtered and evaporated under vacuum and extracted with EtOAc thrice. The extract was partitioned between petroleum ether, and 90% aqueous MeOH to obtain the crude extract (43.0 g). The crude extract was subjected to silica gel column chromatography (CC) eluted with petroleum ether/EtOAc in a gradient eluent (v/v, 50:1, 30:1, 20:1, 10:1, 5:1, 1:1, 0:1) to obtain 8 fractions (fractions 1–8). Fr. 2 was subjected to ODS CC eluted with MeOH/H_2_O in a gradient eluent (1:9, 2:3, 3:2, 4;1, 9:1), to give 3 sub fractions (fr. 2.1–2.3). Fr. 2.2 was further purified by HPLC eluting with MeOH/H_2_O (60:40) to afford **9** (5.5 mg), **10** (4.7 mg), and **11** (3.8 mg). Fr. 3 was purified by Sephadex LH-20 (CH_3_Cl/MeOH, 1:1) to give 3 sub fractions (fr. 3.1–3.3). Fr. 3.2 was further purified by silica gel CC eluted with petroleum ether/EtOAc in a gradient eluent (v/v, 10:1) to obtain **5** (20.1 mg) and **8** (10.3 mg). Fr. 4 was subjected to an ODS column (MeOH/H_2_O: 10–100%) to give 4 sub fractions (fr. 4.1–4.4). Fr. 4.2 was further purified by HPLC eluting with MeOH/H_2_O (50:50, 1%0TFA) to afford **3** (20.8 mg). Compounds **16** (20.3 mg) and **17** (43.2 mg) were purified from Fr. 4.4 by HPLC (35% MeCN, 1%0 TFA). Fr.5 was purified by ODS CC (MeOH/H_2_O: 10–100%) and HPLC (35% MeCN, 1%0 TFA) to afford **1** (7.4 mg), **2** (4.3 mg) and **6** (5.5 mg). Compound **12** (2.3 mg) was isolated from Fr.6 by an ODS CC and followed by HPLC using 10% MeCN. Fr.7 was subjected to Sephadex LH-20 CC (CH_3_Cl/MeOH, 1:1) to provide 4 subfractions (Frs.7.1–7.4). Fr. 7.1 was further purified on HPLC by 25% MeCN (2.5 mL/min) to give **13** (32.3 mg), **14** (43.7 mg). Fr. 7.2 was purified on HPLC by 10% MeCN to give **7** (15 mg). Finally, compounds **15** (4.4 mg) and **4** (3.5 mg) were isolated from Fr.7.3 and Fr. 7.4 by HPLC using 25% MeCN and 30% MeCN, respectively.

Coleophomone E (**1**): Yellow amorphous solid; [α]25 D+14.1 (*c* 0.31, MeOH); UV (MeOH) λ_max_ (log ε) 266 (3.16), 322 (2.25) nm, HRESIMS *m/z* 343.1541 [M + H]^+^ (calcd for C_20_H_23_O_5_, 343.1541); ^1^H and ^13^C NMR data, Table 1.

**Table 1:** ^1^H, ^13^C NMR data of **1**–**4** (500/125 MHz, in DMSO-*d*_6_, δ ppm, and *J* in Hz).

**No**.	**1**		**2**		**No**.	**3**		**4**	
	**δ_H_**	**δc, type**	**δ_H_**	**δc, type**		**δ_H_**	**δc, type**	**δ_H_**	**δc, type**
1	5.50, s	67.7, CH	5.70, s	66.3, CH	1		158.0, C		155.7, C
2		154.7, C		146.2, C	2	6.22, s	110.3, CH		120.9, C
3	6.84, s	116.8, CH		148.5, C	3		140.6, C		137.4, C
4		147.4, C		141.0, C	4	6.35, s	111.9, CH	6.27, d (1.5)	110.3, CH
5	6.63, s	115.6, CH	6.68, s	118.0, CH	5		158.9, C		157.2, C
6		157.2, C		150.7, C	6	6.14, s	103.2, CH	6.22, d (1.5)	102.7, CH
7		121.7, C		123.1, C	7	2.18, s	21.5, CH_3_	2.20, s	19.5, CH_3_
8		200.1, C		201.2, C	8		167.7, C		167.1, C
9		71.8, C		71.6, C	9	3.67, s	52.2, CH_3_	3.69, s	51.9, CH_3_
10		196.9, C		196.6, C	10	-	-		169.2, C
11		138.4, C		138.3, C	1′		155.8, C		154.5, C
12	6.63, d (5.5)	141.9, CH	6.62, d (5.5)	141.8, CH	2′		117.1, C		116.9, C
13	2.61, m	32.4, CH_2_	2.66, m	32.3, CH_2_	3′		138.9, C		138.6, C
14	4.39, dd (9.2, 5.9)	67.4, CH	4.34, dd (9.5, 5.0)	67.8, CH	4′	6.41, s	112.8, CH	6.47, d (2.0)	112.9, CH
15	2.72, m	27.3, CH_2_	2.72, m	27.3, CH_2_	5′		159.7, C		159.4, C
16	5.03, t (6.8)	121.3, CH	5.06, t (7.0)	121.3, CH	6′	6.14, s	103.9, CH	6.16, d (2.0)	104.0, CH
17		132.2, C		132.3, C	7′	2.20, s	19.9, CH_3_	2.22, s	19.5, CH_3_
18	1.54, s	17.5, CH_3_	1.55, s	17.5, CH_3_	5OH	9.95, br.s		10.02, br.s
19	1.65, s	25.5, CH_3_	1.66, s	25.5, CH_3_	5'OH	9.50, br.s		9.93, br.s
20	2.31, s	21.7, CH_3_	2.22, s	16.2, CH_3_	NH_2_			7.45, 7.35, br.s
21			3.72, s	60.6, CH_3_				

Coleophomone F (**2**): Yellow amorphous solid; [α]25 D+13.2 (*c* 0.46, MeOH); UV (MeOH) λ_max_ 220 (3.85), 266 (3.20), 313 (2.43) nm, HRESIMS *m/z* 373.1638 [M + H]^+^ (calcd for C_21_H_25_O_6_, 373.1646); ^1^H and ^13^C NMR data, Table 1.

Diorcinol L (**3**): Yellow amorphous solid; HRESIMS *m/z* 289.1070 [M + H]^+^ (calcd for C_16_H_17_O_5_, 289.1071); ^1^H and ^13^C NMR data, Table 1; The structure of **3** have been deposited in the Cambridge Crystallographic Data Centre as supplementary publication number CCDC 1502322.

Diorcinol M (**4**): Yellow amorphous solid; HRESIMS *m/z* 332.1127 [M + H]^+^ (calcd for C_17_H_18_NO_6_, 332.1129). ^1^H and ^13^C NMR data, Table 1.

1-Hydroxy-6-methyl-11-methoxy-8-hydroxymethylxanthone (**5**): Yellow needle crystals; HRESIMS *m/z* 287.0913 [M + H]^+^ (calcd for C_16_H_15_O_5_, 287.0919); ^1^H and ^13^C NMR data, Table 2.

**Table 2:** ^1^H, ^13^C NMR data of **5**–**7** (500/125 MHz, in CDCl_3_, δ ppm, *J* in Hz).

**No**.	**5**		**No**.	**6**		**No**.	**7**	
	**δ_H_**	**δc, type**		**δ_H_**	**δc, type**		**δ_H_**	**δc, type**
1		161.7, C	1		126.5, C	1		154.3, C
2	6.71, d (8.0)	110.4, CH	2	6.71, s	116.9, CH	2	6.72, d (8.0)	114.9, CH
3	7.49, t (8.0)	136.7, CH	3		146.9, C	3	7.04, td (8.0, 2.0)	127.4, CH
4	6.80, d (8.0)	106.5, CH	4		131.2, C	4	6.77, t (7.5)	118.6, CH
4a		155.4, C	4a		141.6, C	5	7.29, dd (7.5, 1.0)	128.7,CH
5	7.18, s	119.5, CH	5a		164.7, C	6		129.8, C
6		142.4, C	6		97.3, C	7	5.01, d (3.5)	67.5, CH
7		153.5, C	7	6.36, d (1.5)	101.0, CH	8	3.32, dd (6.0, 3.5)	78.0, CH
8		133.9, C	8		165.5, C	9	3.58, dq (6.0, 6.0)	66.8, CH
8a		117.8, C	9	7.21, d (1.5)	104.5, CH	10	1.11, d (6.0)	19.7, CH_3_
9		184.2, C	9a		138.8, C	6-OH	9.33, br.s
9a		108.9, C	9b		109.4, C		
10a		153.8, C	10	2.60, s	24.8, CH_3_		
11	4.99, s	56.7, CH_2_	11		164.2, C		
12	2.40, s	17.2, CH_3_	COO*H*	11.84, s			
13	3.81, s	62.6, CH_3_					
1-OH	12.53, s						
11-OH	4.37, br.s						

Porric acid E (**6**): Colorless needle crystals; HRESIMS *m/z* 275.0544 [M + H]^+^ (calcd for C_14_H_11_O_6_, 275.0550); ^1^H and ^13^C NMR data, Table 2.

7-(2-Hydroxyphenyl) butane-7,8,9-triol (**7**): Yellow oil; HRESIMS *m/z* 221.0781 [M + Na]^+^ (calcd for C_10_H_14_NaO_4_, 221.0784); ^1^H and ^13^C NMR data, Table 2.

### ECD calculation

The eight possible stereoisomers (**a-h**) of **1** were initially performed using Confab (O'Boyle et al., 2011) with systematic search at MMFF94 force field. Room-temperature equilibrium populations were calculated according to Boltzmann distribution law. The conformers with Boltzmann-population of over 1% were chosen were chosen for ECD calculations using Gaussian 09 (Frisch et al., 2009) software, and the stable conformers were initially optimized at the B3LYP/6-311G(d,p) in methanol using the IEFPCM model. Vibrational frequency analysis confirmed the stable structures. Under the same condition, the ECD calculation was conducted using Time-dependent Density functional theory (TD-DFT). Rotatory strengths for a total of 30 excited states were calculated. The ECD spectrum was simulated in SpecDis (University of Würzburg) with a half-bandwidth of 0.3–0.4 eV, according to the Boltzmann-calculated contribution of each conformer after UV correction.

### NMR calculation

The two stereoisomers **1e** and **1f** were delivered to geometry optimization at B3LYP/6-31+G(d,p) in gas phase. The theoretical calculation of NMR was conducted using the Gauge-Independent Atomic Orbitals (GIAO) method at mPW1PW91/6-311+G(2d,p) in methanol by the IEFPCM model. Finally, the TMS-corrected NMR chemical shift values were averaged according to Boltzmann distribution for each conformer and fitting to the experimental values by linear regression. The calculated ^13^C-NMR chemical shift values of TMS in methanol were 0 ppm.

### Cytotoxicity assay

The cytotoxic activities of **1-17** were screened against the growth panel of 10 tumor cell lines (K562, MCF-7, A549, Huh-7, H1975, HeLa, HL7702, HL60, MOLT-4, and DU145) (Bergeron et al., 1994) (Supplementary Material).

### COX-2 inhibitory activity assay

According to the manufacturer's instructions. The test compounds were dissolved in DMSO and the final concentration was set as 10 μM. The percentage inhibition has been calculated by comparison with control incubations (Tian et al., 2015b) (Supplementary Material).

## Results and discussion

Compound **1** was assigned a molecular formula of C_20_H_22_O_5_ (10 degrees of unsaturation) by HRESIMS (*m/z* 343.1541 [M + H]^+^). Its NMR spectra showed resonances for three methyls, two methylenes, two oxygenated sp^3^ methine, one sp^3^ quaternary carbon, four sp^2^ methine, six sp^2^ quaternary carbon, and two ketocarbonyl carbons (δ_C_ 200.1 and 196.9) (Table 1). These data suggested a tricyclic skeleton of **1**. The ^1^H NMR spectrum exhibited aromatic signals at δ_H_ 6.84 (s, H-3), 6.63 (s, H-5) indicating the presence of a tetrasubstituted aromatic ring. The HMBC correlations of H-3/C-2, C-4, C-5, C-7, and C-20, H_3_-20 (δ_H_ 2.31, s)/C-4, and C-5, and H-5/C-6, and C-7 suggested a methyl (C-20) and a hydroxy at C-4 and C-6, respectively. The HMBC correlations of H-1 (δ_H_ 5.50, s)/C-2, C-3, C-4, C-6, C-7, C-8 (δ_C_ 200.1) and C-9 (δ_C_ 71.8), and H-5/C-8 indicated linkage of ring B and the connection of C-1 to C-2 and C-7 to C-8. The COSY cross-peaks of H-13 (δ_H_ 2.61, m) /H-14 (δ_H_ 4.39, dd, *J* = 9.2, 5.9 Hz), and H-12 (δ_H_ 6.33, d, *J* = 5.9 Hz) delineated the spin system C_12_-C_13_-C_14_. Moreover, the HMBC correlations of H-12/C-10 and C-14, H-13/C-9, C-11, and C-12, and H-14/C-1, C-8 and C-9 indicated the presence of a α,β-unsaturated ketone (ring C) and rings B/C are connected by C-9. Finally, a 2-methyl-2 butene group was attached to C-11 (δ_C_ 138.4) by the evidence of the HMBC correlations of H_3_-18 (δ_H_ 1.54, s)/C-16 (δ_C_ 121.3), C-17 (δ_C_ 132.2), and C-19 and H_2_-15 (δ_H_ 2.72, s)/C-10, C-11, C-12, C-13, C-16, and C-17.

**Figure 3:**
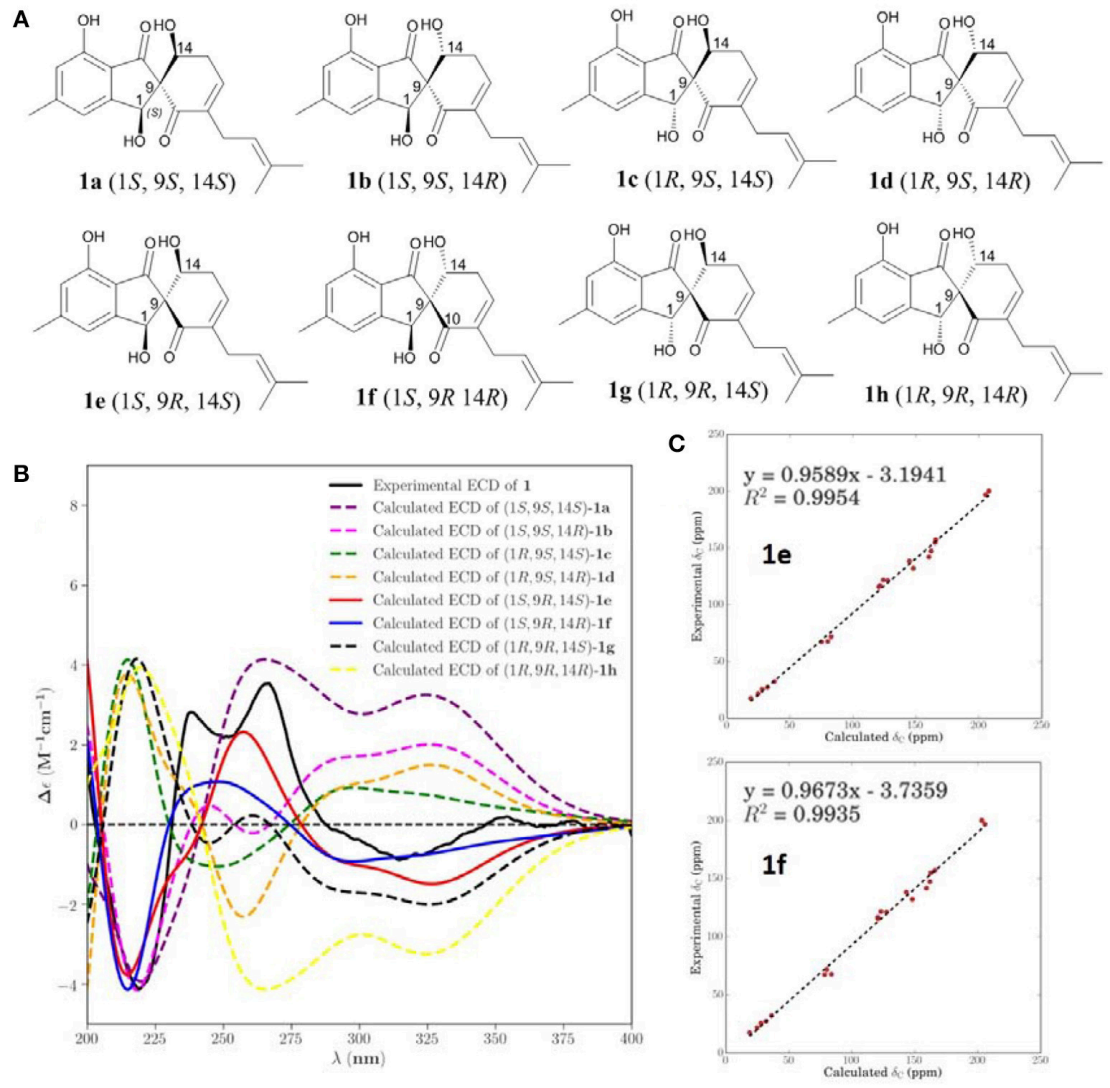
Calculated ECD and NMR studies of **1**. **(A)** Eight possible stereoisomers **(a-h)** of **1**. **(B)** Calculated ECD spectra of configurations **a**-**h** were compared with the experimental ECD spectra. **(C)** Linear regression fitting of calculated ^13^C-NMR chemical shifts of **1e** and **1f** with experimental values.

NOESY correlations and Mosher method were failure to determine the configurations of **1**, so theoretical calculations were used to solve it. There are eight possible stereoisomers (**a-h**) of **1**, as shown in Figure 3A. Computational studies of electron circular dichroism (ECD) were carried out. All stereoisomers (**a–h**) were selected for theoretical calculations using time dependent density functional theory (TDDFT) B3LYP/6-311G (d,p) level with the IEFPCM model in MeOH (Tables S1, 2, 4). A comparison of the experimental spectrum of **1** with the calculated ECD spectra of eight possible stereoisomers (**a-h**) were presented in Figure 3B. The measured ECD curve exhibits two negative Cotton effects (CEs) at 219 and 315 nm, and two positive cotton effects at 238 and 267 nm, matching well with the calculated ECD curve of **1e** (1*S*, 9*R*, 14*S*) and **1f** (1*S*, 9*R*, 14*R*). Then, computed ^13^C-NMR chemical shifts was carried out to define the stereochemistry of C-14. Computed ^13^C-NMR chemical shifts of each conformer were first Boltzmann-weighted averaged, and then fitted to experimental values by Ordinary Least Squares (OLS) Linear Regression method in order to remove systematic error that results from the conformational search and random error from experimental conditions (Tables S3, 5). As a result, the computed chemical shift for C-14 of **1e** is δ_C_ = 68.7 ppm, with only a deviation of 1.3 ppm from the experimental value (δ_C_ = 67.4 ppm) (Table S8). All in all, the computed chemical shifts of **1e** showed good agreement with the experimental values and has the higher *R*^2^ and *R*${}_{\text{adj}}^{2}$ values than that of **1f**, which suggesting that **1e** (1*S*, 9*R*, 14*S*) be the true isomer of **1** (Figure 3C, Table S7).

The molecular formula of **2** was determined as C_21_H_24_O_6_ by its HRESIMS (*m/z* 373.1638 [M + H]^+^), corresponding to 10 units of unsaturation. Its UV and NMR date were similar to those of **1**, except for the presence of a methoxy (δ_H_ 3.72, δ_C_ 60.6) in **2** (Table 1). The extra methoxy (C-21) was located at C-3 by HMBC correlations from H_3_-21 to C-3 (δ_C_ 148.5) (Figure 2). The absolute configuration of **2** was suggested as (1*S*,9*R*,14*S*), as its chemical shift, coupling constant, optical rotation and CD effect (Table 1, Figure S20) almost the same as those of **1**.

**Figure 4:**
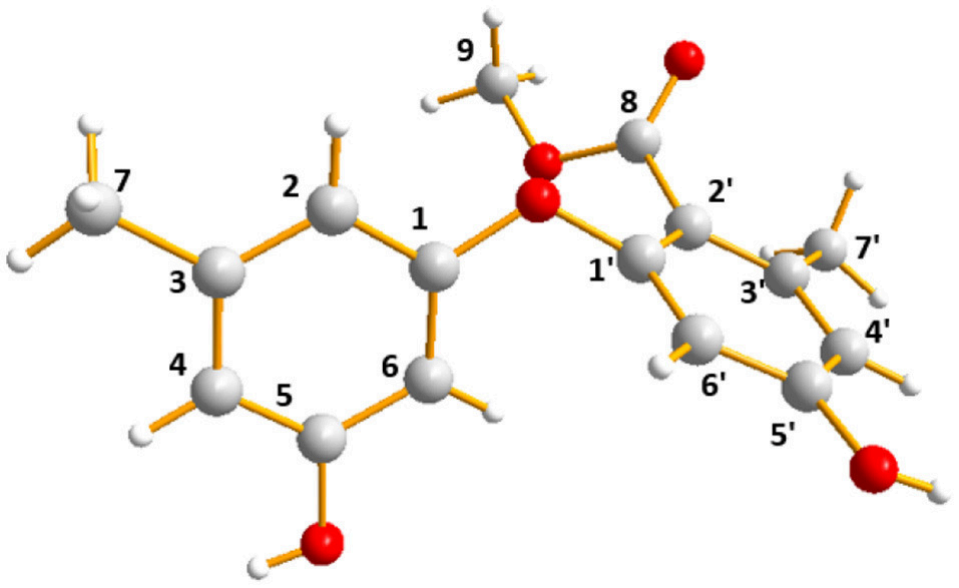
X-ray crystallographic structure of **3**.

The molecular formula of **3** was assigned as C_16_H_16_O_5_ by its HRESIMS ion peak at *m/z* 289.1070 [M + H]^+^ and NMR date. The ^1^H NMR spectrum of **3** exhibited two hydroxyl proton at δ_H_ 9.95 and 9.50, five aromatic signals at δ_H_ 6.41, 6.35, 6.22, and 6.14 × 2, one *O*-methyl at δ_H_ 3.67, and two single methyls at δ_H_ 2.18 and 2.20 (Table 2). Analysis of the ^13^C and DEPT-135 NMR spectra of **3** indicated the presence of 16 carbons, including one carbonyl carbon (δ_C_ 167.7), 12 aromatic carbons (four oxygenated ones at δ_C_ 159.7, 158.9, 158.0 and 155.8), one methoxy and two methyls. These spectra of **3** were similar to those of diorcinol (Tian et al., 2015b), except for the presence of a metheyl formate group (one carbonyl carbon at δ_C_ 167.7 and one methoxy at δ_H_ 3.67/δ_C_ 52.2). However, relying solely on the NMR date, the location of metheyl formate was more difficult to determine. In order to determine location of metheyl formate of **3**, a single-crystal X-ray diffraction pattern was obtained using the anomalous scattering of Cu Kα radiation shows an ORTEP drawing (Figure 4, Table S9) and unambiguously determined metheyl formate at C-2′. Thus, the structure of **3** was determined, and named as diorcinol L.

**Figure 5:**
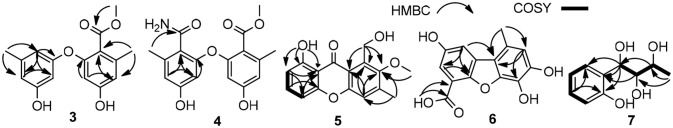
Key COSY and HMBC correlations of **3**–**7**.

Compound **4** was obtained as brown powder. The molecular formula of was determined as C_17_H_17_NO_6_ by its HRESIMS (*m/z* 332.1131 [M + H]^+^), which corresponded to ten units of unsaturation. The ^1^H and ^13^C NMR data of **4** were similar to those of **3**, except for the presence of one amide [δ_C_ 169.2 (*C*ONH_2_)/ δ_H_ 7.45, 7.35 (CON*H*_2_)] (Table 1). The extra amide was determined at C-2 by the HMBC correlations of H-4, H-6/C-2, as well as H_3_-7/C-2, C-3, C-4, and C-10 (Figure 5). Hence, the structure of was elucidated and the trivial name diorcinol M was assigned.

Compound **5** was isolated as pale yellow needle-like crystals. Its molecular formula was determined to be C_16_H_14_O_5_, by HR-ESI-MS, indicating 10 degrees of unsaturation. The 1D NMR data (Table 3) of **5** contained resonances for one carbonyl carbon, eight sp^2^ quarternary carbons, four sp^2^ methines, one sp^3^ methylene, two sp^3^ methys. Comparison of UV-vis and NMR data with those of 1-Hydroxy-6-methyl-8-hydroxy- methylxanthone (**8**) revealed a high degree of similarity skeleton, where the only obvious differences is in the presence of one methoxy (δ_H_ 3.81; δ_C_ 62.6) and low-field chemical shift of C-7 (from δ_C_ 127.2 in **8** to 153.5 in **1**). Meanwhile, this difference can also be ascribed by the HMBC correlations (Figure 5) of δ_H_ (3.81, s, H-13) with δ_C_ (153.5, C-7) and requirements of HRESIMS spectrum. Thus, the structure of **5** was determined and named as 1-hydroxy-6-methyl-11-methoxy-8-hydroxymethylxanthone.

**Table 3:** Cytotoxic results of the compounds (IC_50_, μM).

**Comp**.	**K562**	**MCF-7**	**A549**	**Huh-7**	**H1975**	**HeLa**	**HL7702**	**HL60**	**MOLT-4**	**DU145**
3	43.5	10.5	17.7	5.7	15.3	7.1	68.2	9.6	NA	9.1
8	NA	141.0	128.0	122.0	NA	14.3	33.8	NA	NA	NA
TSA	0.1	0.7	0.3	0.08	0.09	0.08	0.09	0.04	0.03	0.04

*NA, No active (IC_50_ > 200 μM)*.

Compound **6** showed a molecular ion peak at *m/z* 275.0544 [M + H]^+^ in the HR-ESI-MS, in accordance with the molecular formula C_14_H_10_O_6_, which was also supported by NMR data. The ^1^H NMR data displayed one sharp hydroxy proton singlet (11.84, s, COO*H*), two aromatic protons at δ_H_ 7.21 (1H, d, *J* = 1.5 Hz), 6.71 (1H, s), 6.36 (1H, d, *J* = 1.5 Hz), and a singlet methyl at δ_H_ 2.60 (3H, s). The ^13^C and DEPT-135 NMR spectra (Table 2) showed 14 carbons, including one carboxyl group (δ_C_ 164.2), three aromatic methine carbons (δ_C_ 116.9, 104.5, 101.0), 11 aromatic quaternary carbons (δ_C_ 165.5, 164.7, 146.9, 141.6, 138.8, 131.2, 126.5, 109.4, 97.3), and one methyl (δ_C_ 24.8). The aforementioned NMR data showed **1** was closely related structurally to the porric acid C (Carotenuto et al., 1998). The only difference was the substituent of C-4, the chemical shift to low field of C-4 (δ_C_ 131.2 in **4**; δ_C_ 101.0 in porric acid C), the HMBC correlation (Figure 5, Table 2) from H-2 (δ_H_ 6.71, s) to C-3 (δ_C_ 146.9) and C-4 (δ_C_ 131.2) requirements of HRESIMS spectrum suggested that a hydroxy proton group was connected to C-4. Thus, the structure of **6** was determined and named porric acid E.

Compound **7** was obtained as a yellow oil. Its HRESIMS gave the molecular formula C_10_H_14_O_4_, requiring four degrees of unsaturation. The ^1^H NMR spectrum (Table 2) exhibited aromatic signals at δ_H_ 7.29 (dd, *J* = 7.5, 1.0 Hz, H-5), 7.04 (td, *J* = 8.0, 2.0 Hz, H-3), 6.77 (t, *J* = 8.0, Hz, H-4), and 6.72 (d, *J* = 8.0 Hz, H-2), indicating the presence of a disubstituted aromatic ring. The substitutions of a trihydroxybutyl group (C-7—C-10) and a hydroxy (δ_H_ 9.33, br.s, OH-6) at C-1 (δ_C_ 154.3) and C-6 (δ_C_ 129.8), were assigned by the COSY and HMBC interactions (Figure 5, Table 2).

### Biological activity

Cytotoxic activities of the isolated compounds **1**–**17** and trichostatin A (TSA, positive control) against 10 tumor cell lines (K562, MCF-7, A549, Huh-7, H1975, HeLa, HL7702, HL60, MOLT-4, and DU145) were tested. Among all of them, **3** displayed a wide range of cytotoxicities with IC_50_ values in the range of 5.7–68.2 μM, and **8** showed weak selective cytotoxicities against HeLa and HL7702 cells (Table 3). Furthermore, compound **6** displayed COX-2 inhibitory activity with the prominent IC_50_ values of 3.3 μM. Celecoxib was used as the positive control with IC_50_ values of 0.01 μM.

## Conclusion

In conclusion, 17 phenol derivatives were isolated from the EtOAc extract of a marine sponge-derived fungus *Didymellaceae* sp. SCSIO F46. Coleophomones E and F (**1** and **2**) possess unprecedented spiro [cyclohexane-1,2′-inden] moiety, which is relatively seldom in natural products. Other new compounds **3-7** represent common types of phenol derivatives, which are widely found in fungal metabolites. Amongst, compounds **3** and **8** displayed a wide range of cytotoxicities against several tumor cell lines. In addition, **6** displayed significant COX-2 inhibitory activity with the prominent IC_50_ value of 3.3 μM.

## Author contributions

YT designed the experiments and performed the isolation and characterization of all the compounds and wrote the manuscript. XL performed the isolation and purification of the fungal strain. XZ designed the research work and revised the manuscript. YL contributed in project design and manuscript preparation. All authors reviewed the manuscript and approved for submission.

### Conflict of interest statement

The authors declare that the research was conducted in the absence of any commercial or financial relationships that could be construed as a potential conflict of interest.
