# Supplementary material for: Phenol Derivatives From the Sponge-Derived Fungus Didymellaceae sp. SCSIO F46
Source: Front Chem. 2018 Nov 1;6:536. doi: 10.3389/fchem.2018.00536 (PMC6221957; doi:10.3389/fchem.2018.00536)
Supplement: Supplementary file 1 [file Data_Sheet_1.docx]

**Supporting information**

**Phenol Derivatives from the Sponge-Derived Fungus *Didymellaceae* sp. SCSIO F46**

*Yongqi Tian,^1,2^, Xiuping Lin^2^, Xuefeng Zhou^2^*, Yonghong Liu^2,3^**

*^1^ College of Biological Science and Technology, Fuzhou University, Fuzhou 350116, China;*

^2^ *CAS Key Laboratory of Tropical Marine Bio-resources and Ecology, Guangdong Key Laboratory of Marine Materia Medica, South China Sea Institute of Oceanology, Chinese Academy of Sciences, Guangzhou 510301,* *China,*

^3^ *University of Chinese Academy of Sciences, Beijing 100049, China*

*Corresponding Authors: Xuefeng Zhou (xfzhou@scsio.ac.cn), or Yonghong Liu (yonghongliu@scsio.ac.cn).

| **Contents** | **Pages** |
| --- | --- |
| **Figure S1**. The ^1^H NMR spectrum of **1** in DMSO | 4 |
| **Figure S2**. The ^13^C NMR spectrum of **1** in DMSO | 4 |
| **Figure S3**. The DEPT spectrum of **1** in DMSO | 5 |
| **Figure S4**. The HSQC spectrum of **1** in DMSO | 5 |
| **Figure S5**. The COSY spectrum of **1** in DMSO | 6 |
| **Figure S6**. The HMBC spectrum of **1** in DMSO | 6 |
| **Figure S7**. The NOESY spectrum of **1** in DMSO | 7 |
| **Figure S8**. The HRESIMS spectrum of **1** | 7 |
| **Figure S9**. The UV spectrum of **1** | 8 |
| **Figure S10**. Experimental ECD of **1** | 8 |
| **Figure S11**. The ^1^H NMR spectrum of **2** in DMSO | 9 |
| **Figure S12**. The ^13^C NMR spectrum of **2** in DMSO | 9 |
| **Figure S13**. The DEPT spectrum of **2** in DMSO | 10 |
| **Figure S14**. The HSQC spectrum of **2** in DMSO | 10 |
| **Figure S15**. The COSY spectrum of **2** in DMSO | 11 |
| **Figure S16**. The HMBC spectrum of **2** in DMSO | 11 |
| **Figure S17.** The NOESY spectrum of **2** in DMSO | 12 |
| **Figure S18**. The HRESIMS spectrum of **2** | 12 |
| **Figure S19**. The UV spectrum of **2** | 13 |
| **Figure S20**. Experimental ECD of **2** | 13 |
| **Figure S21**. The ^1^H NMR spectrum of **3** in DMSO | 14 |
| **Figure S22**. The ^13^C NMR spectrum of **3** in DMSO | 14 |
| **Figure S23**. The HSQC spectrum of **3** in DMSO | 15 |
| **Figure S24**. The HMBC spectrum of **3** in DMSO | 15 |
| **Figure S25**. The HRESIMS spectrum of **3** | 16 |
| **Figure S26**. The ^1^H NMR spectrum of **4** in DMSO | 16 |
| **Figure S27**. The ^13^C NMR spectrum of **4** in DMSO | 17 |
| **Figure S28**. The HSQC spectrum of **4** in DMSO | 17 |
| **Figure S29**. The HMBC spectrum of **4** in DMSO | 18 |
| **Figure S30**. The HRESIMS spectrum of **4** | 18 |
| **Figure S31**. The ^1^H NMR spectrum of **5** in CDCl_3_ | 19 |
| **Figure S32**. The ^13^C NMR spectrum of **5** in CDCl_3_ | 19 |
| **Figure S33**. The DEPT spectrum of **5** in CDCl_3_ | 20 |
| **Figure S34**. The HSQC spectrum of **5** in CDCl_3_ | 20 |
| **Figure S35**. The HMBC spectrum of **5** in CDCl_3_ | 21 |
| **Figure S36**. The HRESIMS spectrum of **5** | 21 |
| **Figure S37**. The ^1^H NMR spectrum of **6** in DMSO | 22 |
| **Figure S38**. The ^13^C NMR spectrum of **6** in DMSO | 22 |
| **Figure S39**. The DEPT spectrum of **6** in DMSO | 23 |
| **Figure S40**. The HSQC spectrum of **6** in DMSO | 23 |
| **Figure S41**. The HMBC spectrum of **6** in DMSO | 24 |
| **Figure S42**. The HRESIMS spectrum of **7** | 24 |
| **Figure S43**. The ^1^H NMR spectrum of **7** in DMSO | 25 |
| **Figure S44**. The ^13^C NMR spectrum of **7** in DMSO | 25 |
| **Figure S45**. The DEPT spectrum of **7** in DMSO | 26 |
| **Figure S46**. The HSQC spectrum of **7** in DMSO | 26 |
| **Figure S47**. The HMBC spectrum of **7** in DMSO | 27 |
| **Figure S48**. The HRESIMS spectrum of **7** | 27 |
| **Figure S49** Eight possible stereoisomers (**a**-**h**) of **1** | 28 |
| **Table S1** Energies of **1** at MMFF94 force field. | 28-29 |
| **Table S2** Energies of **1** at B3LYP/6-311G(d,p) in methanol | 29-33 |
| **Table S3** Energies of configurations **e** and **f** of **1** at B3LYP/6-31+G(d,p) in gas phase. | 33-34 |
| **Table S4** Standard orientations of configurations of **1** at B3LYP/6-311G(d,p) level in methanol for ECD calculations. | 34-73 |
| **Table S5** Standard orientations of configurations **e** and **f** at B3LYP/6-31+G(d,p) level in gas phase for NMR calculations. | 73-83 |
| **Table S6** UV-shift and σ values for ECD spectrum comparisons. | 83 |
| **Table S7** Statistics of Ordinary Least Squares (OLS) Linear Regression of experimental and computed ^13^C-NMR chemical shifts. | 84 |
| **Table S8** Experimental and computed ^13^C-NMR chemical shifts of **e** and **f** | 85 |
| **Table S9** Crystal data and structure refinement for **3** | 85-86 |
| **Cytotoxicity Assay** | 86 |
| **COX-2 Inhibitory Activity Assay** | 86 |


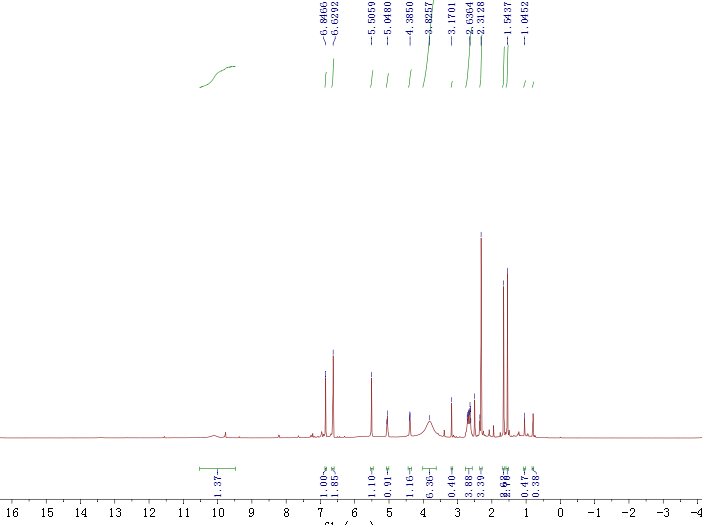


**Figure S1** ^1^H NMR spectrum of compound **1** (500 MHz, DMSO-*d*6)


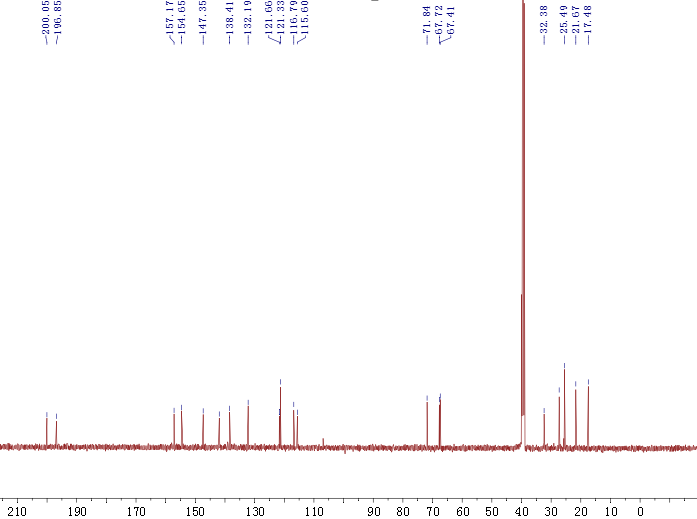


**Figure S2** ^13^C NMR spectrum of compound **1** (125 MHz, DMSO-*d*6)


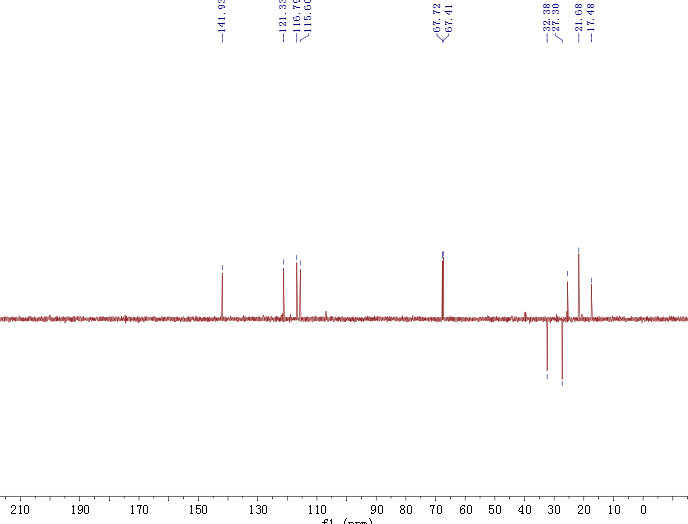


**Figure S3** DEPT-135 spectrum of compound **1** (125 MHz, DMSO-*d*6)


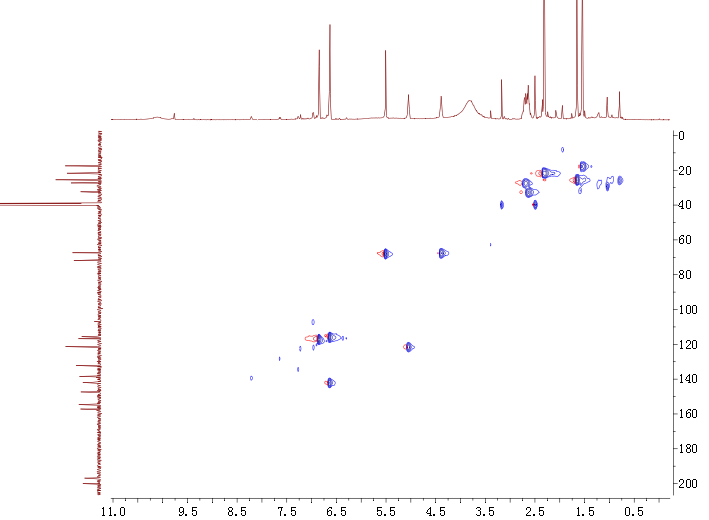


**Figure S4** HSQC spectrum of compound **1**


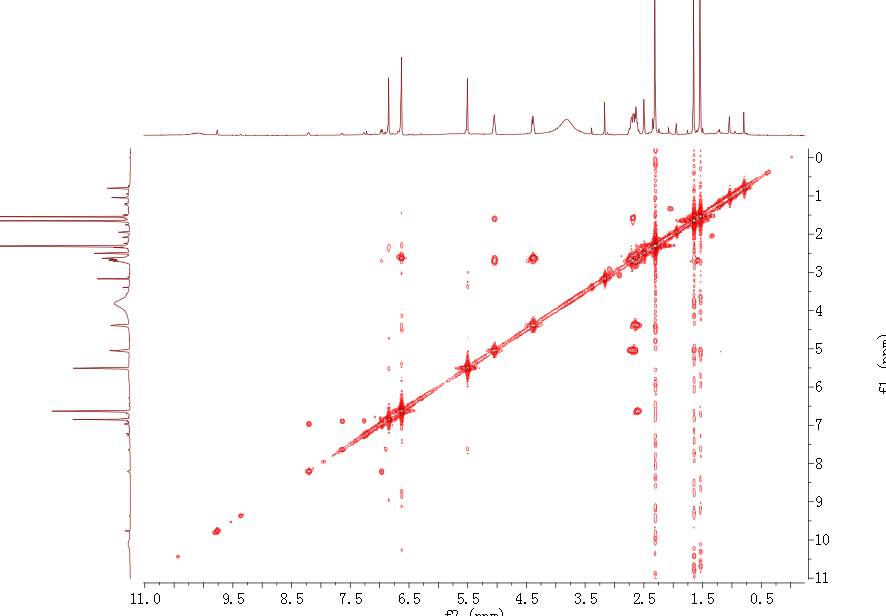


**Figure S5** COSY spectrum of compound **1**


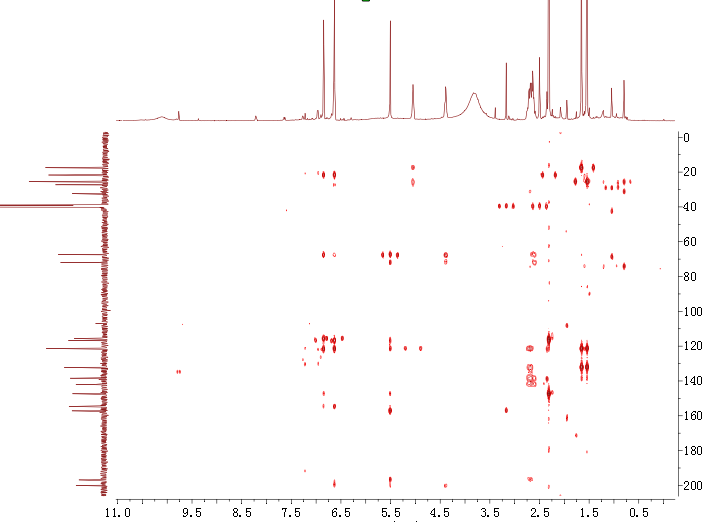


**Figure S6** HMBC spectrum of compound **1**


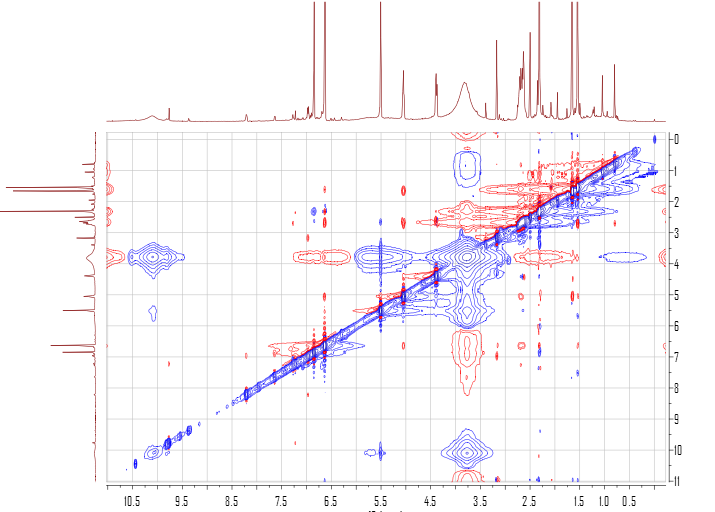


**Figure S7** NOESY spectrum of compound **1**


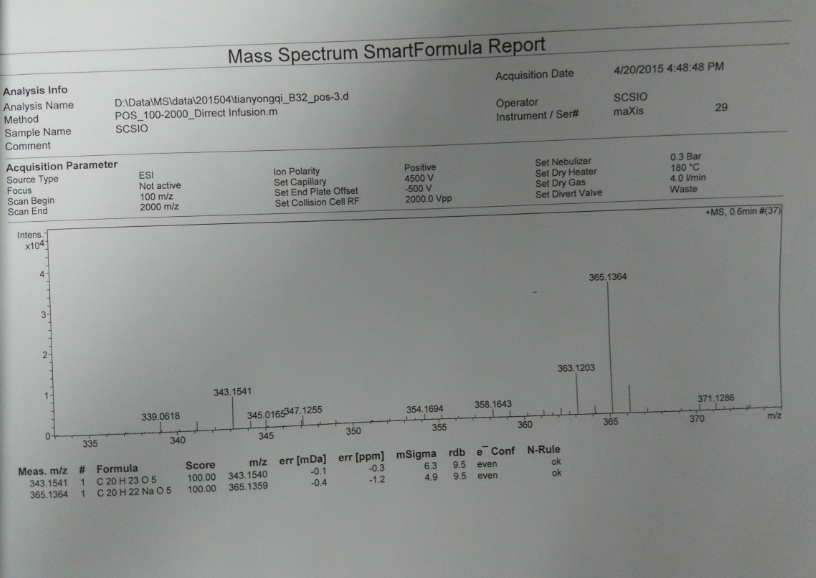


**Figure S8** HRESIMS spectrum of compound **1**


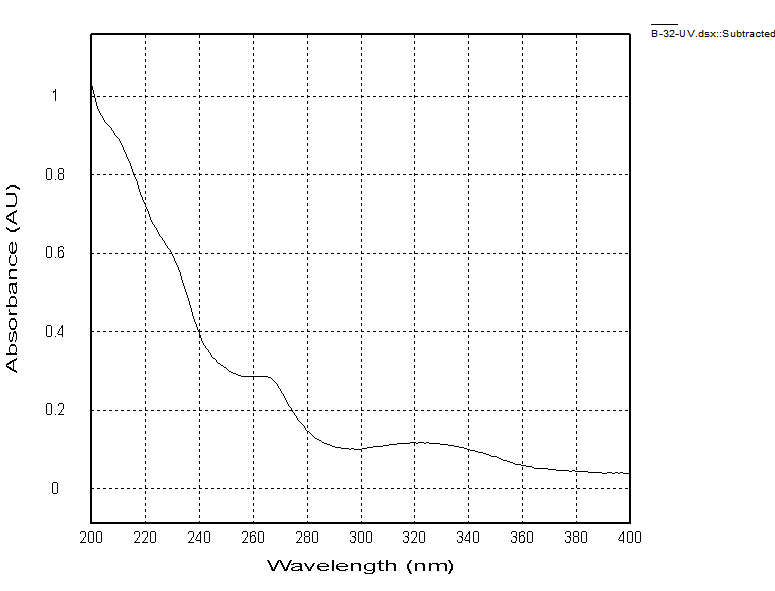


**Figure S9** UV spectrum of compound **1**


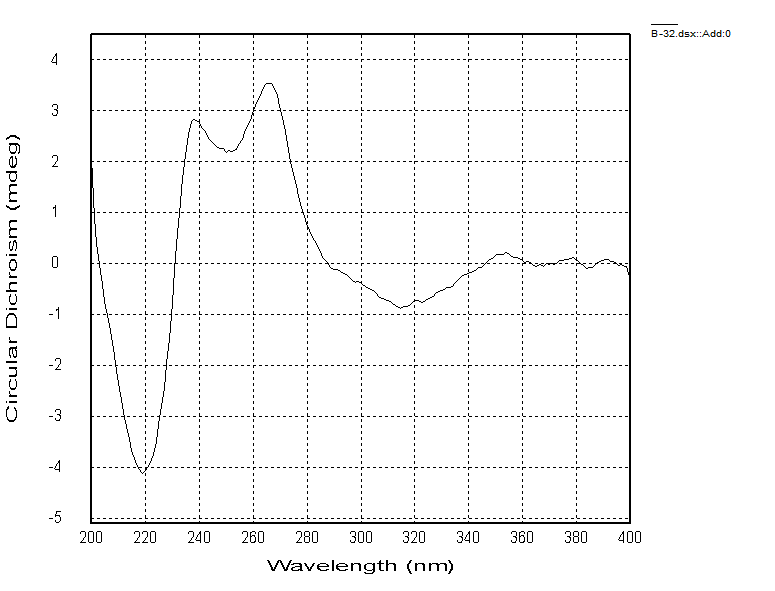


**Figure S10** Experimental ECD of compound **1**


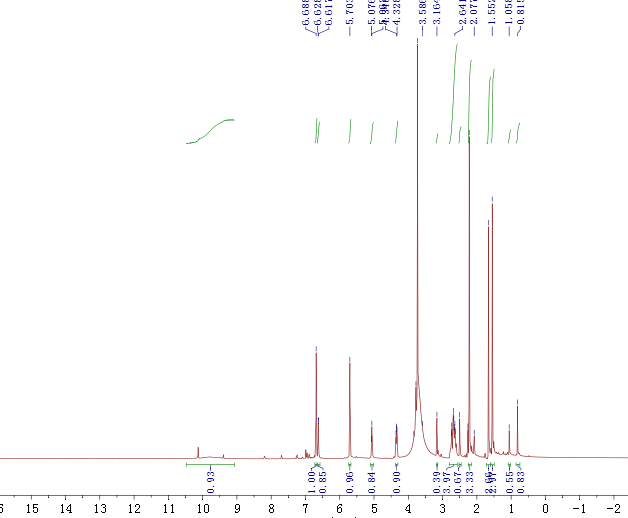


**Figure S11** ^1^H NMR spectrum of compound **2** (500 MHz, DMSO-*d*6)


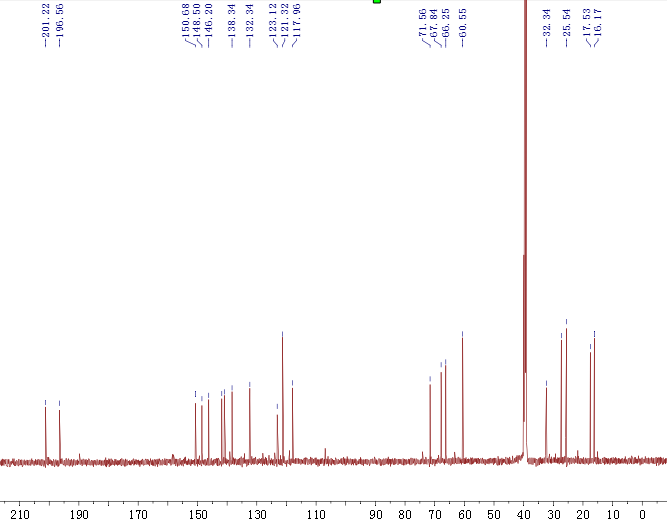


**Figure S12** ^13^C NMR spectrum of compound **2** (125 MHz, DMSO-*d*6)


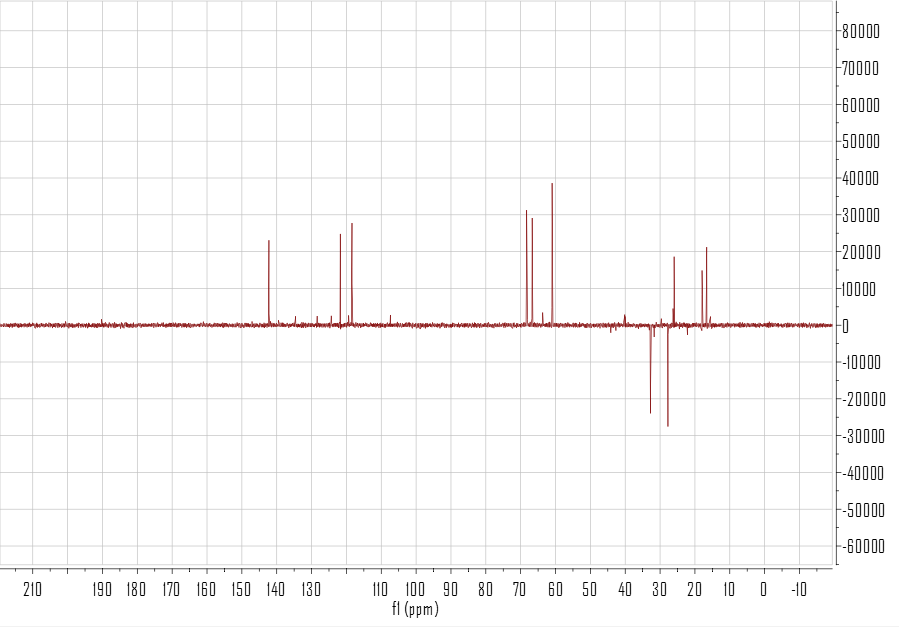


**Figure S13** DEPT-135 spectrum of compound **2**


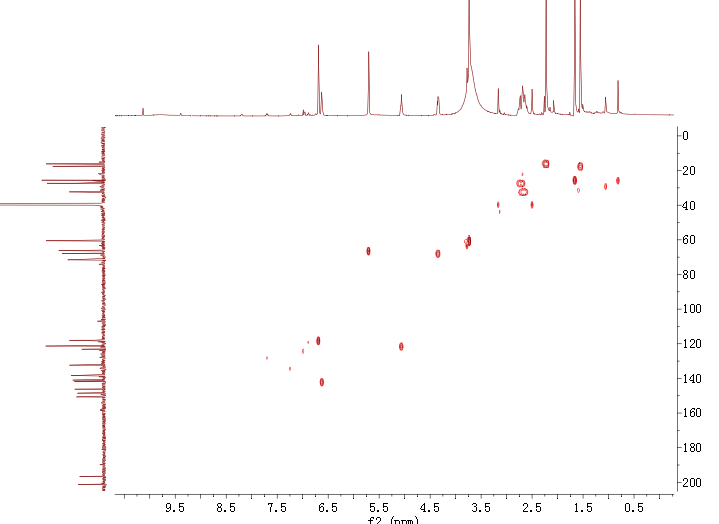


**Figure S14** HSQC spectrum of compound **2**


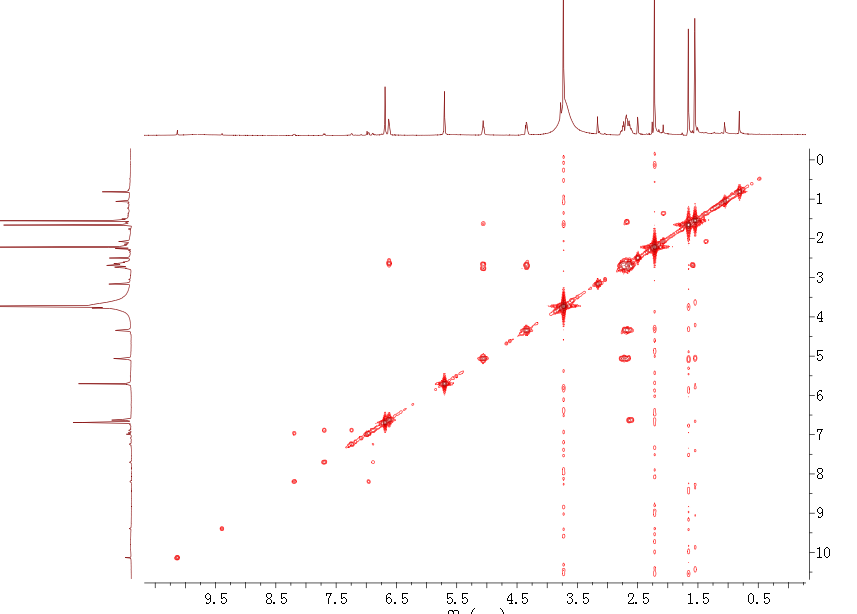


**Figure S15** COSY spectrum of compound **2**


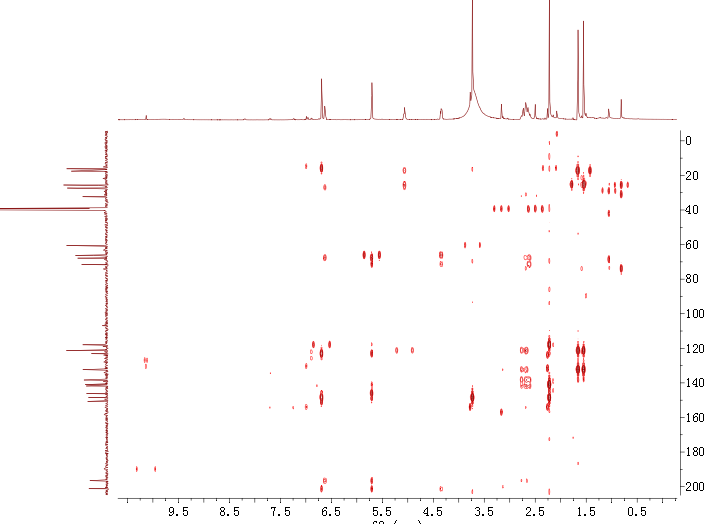


**Figure S16** HMBC spectrum of compound **2**


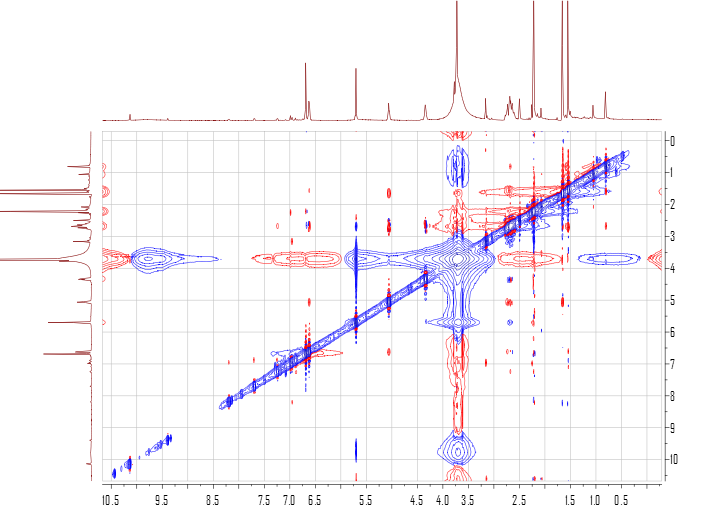


**Figure S17** NOESY spectrum of compound **2**


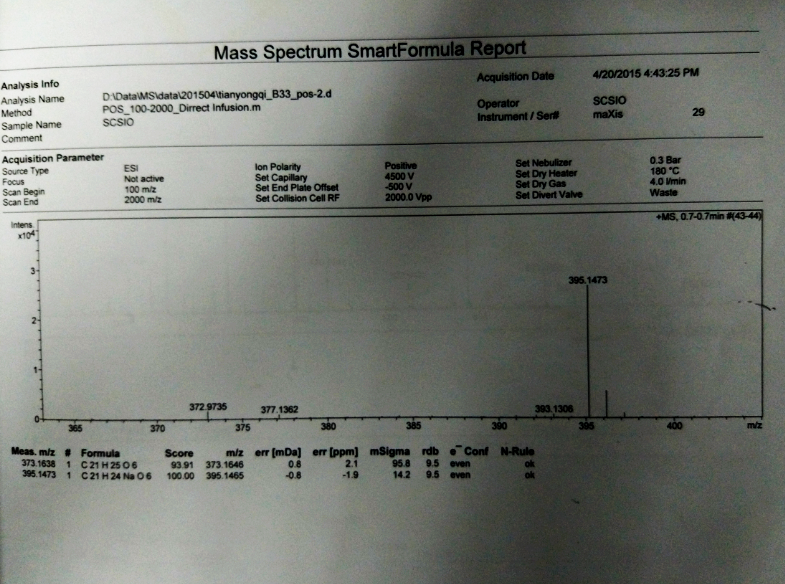


**Figure S18** HRESIMS spectrum of compound **2**


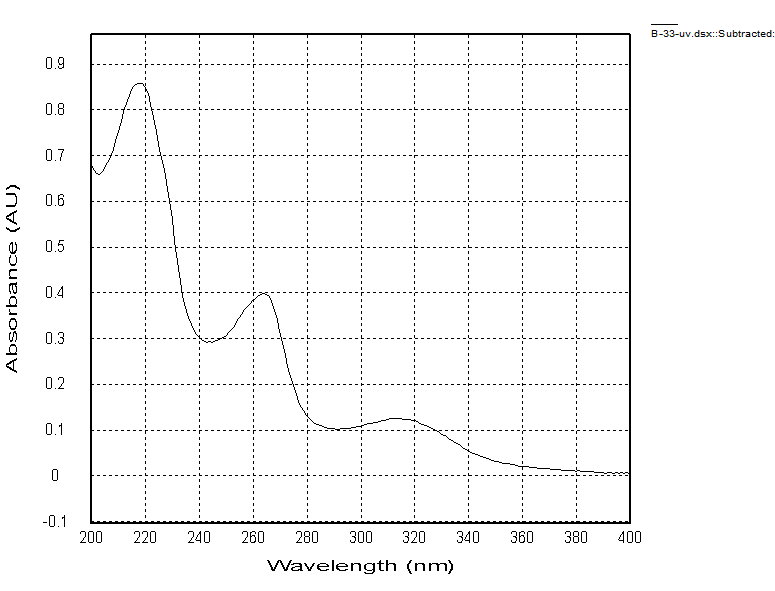


**Figure S19** UV spectrum of compound **2**


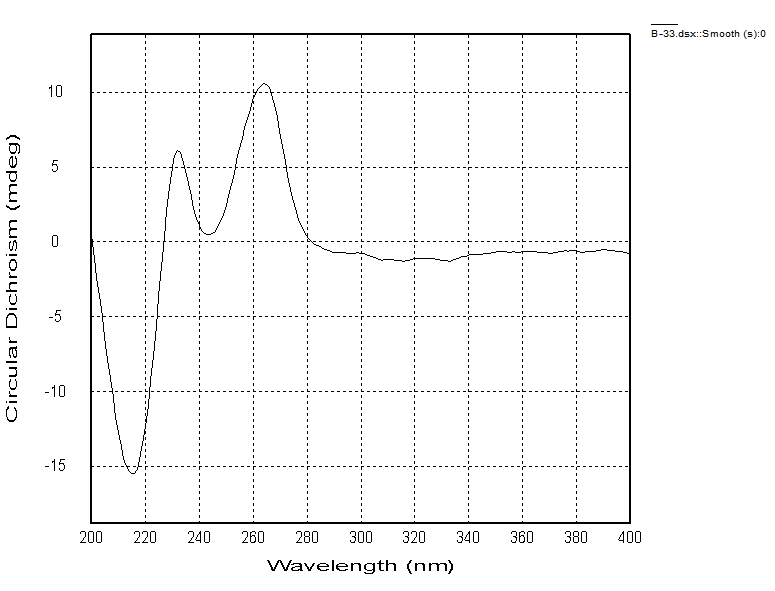


**Figure S20** Experimental ECD of compound **2**


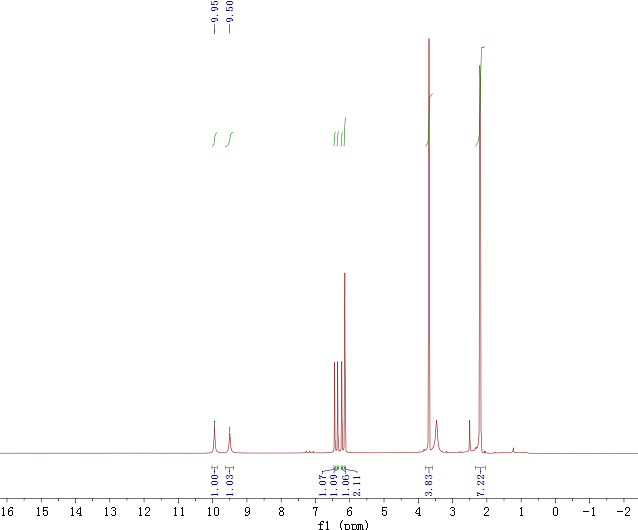


**Figure S21** ^1^H NMR spectrum of compound **3** (500 MHz, DMSO-*d*6)


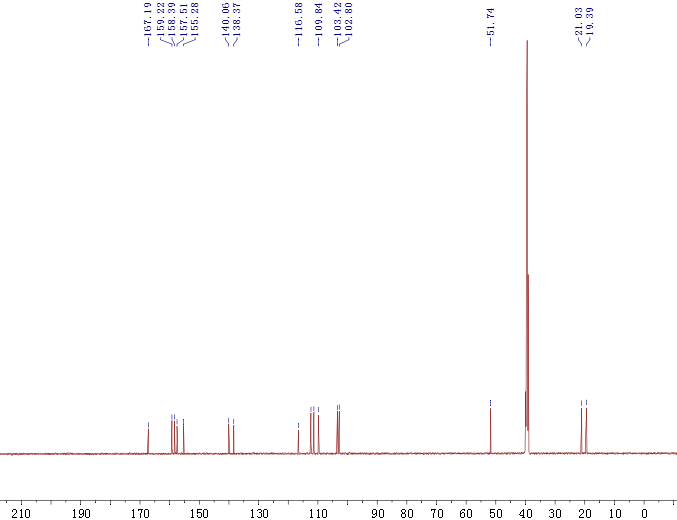


**Figure S22** ^13^C NMR spectrum of compound **3** (125 MHz, DMSO-*d*6)


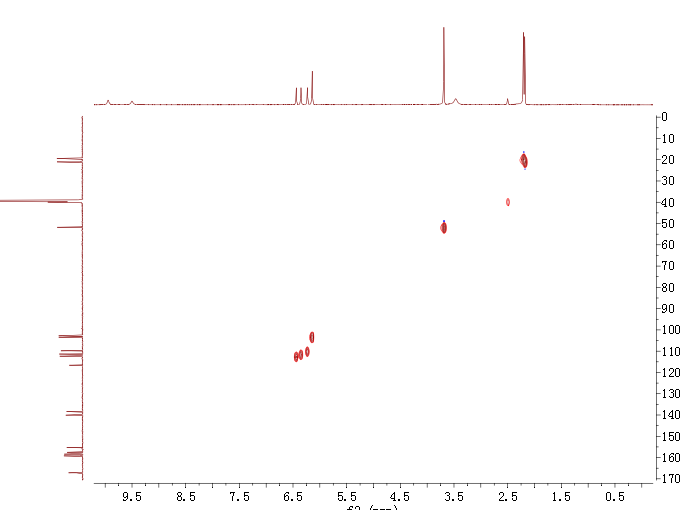


**Figure S23** HSQC spectrum of compound **3**


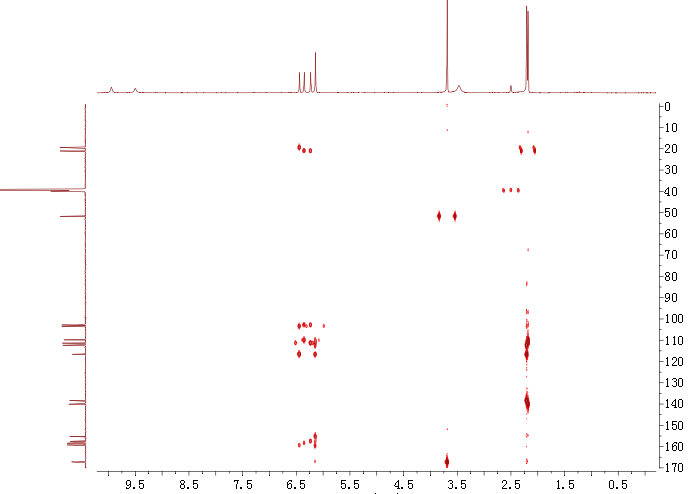


**Figure S24** HMBC spectrum of compound **4**


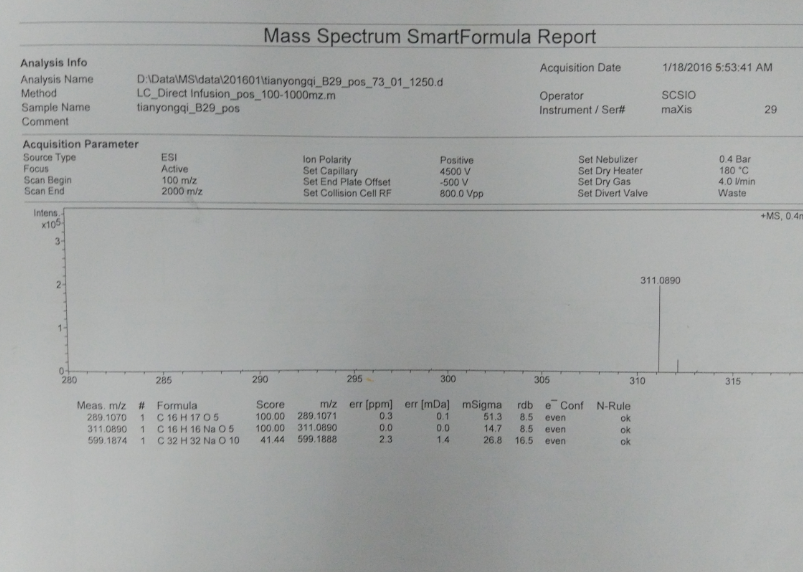


**Figure S25** HRESIMS spectrum of compound **3**


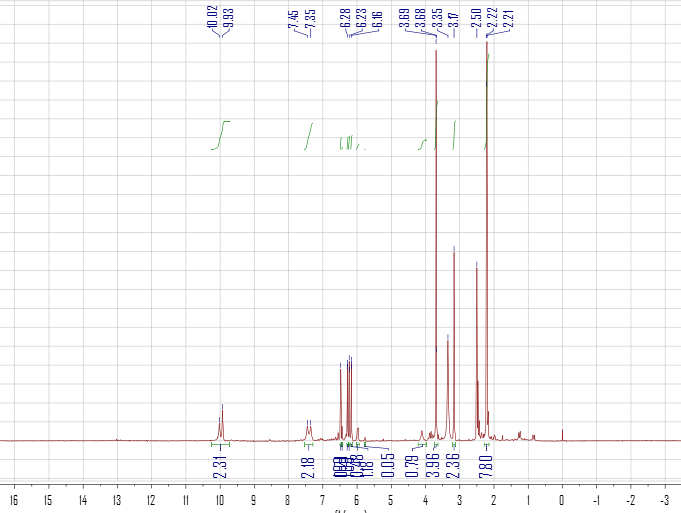


**Figure S26** ^1^H NMR spectrum of compound **4** (500 MHz, DMSO-*d*6)


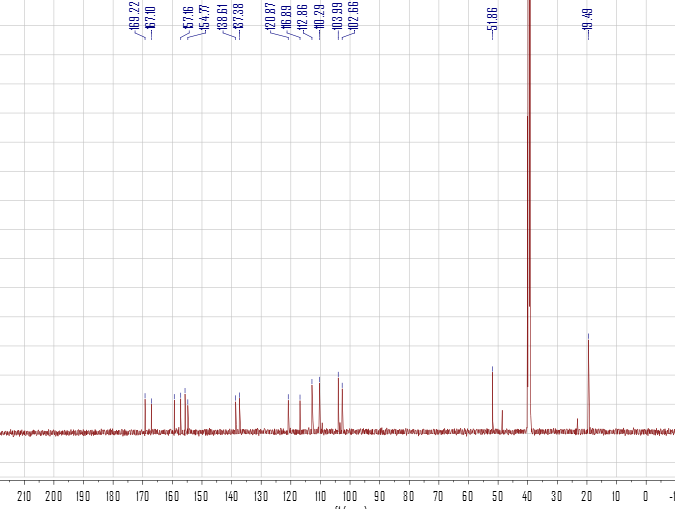


**Figure S27** ^13^C NMR spectrum of compound **4** (125 MHz, DMSO-*d*6)


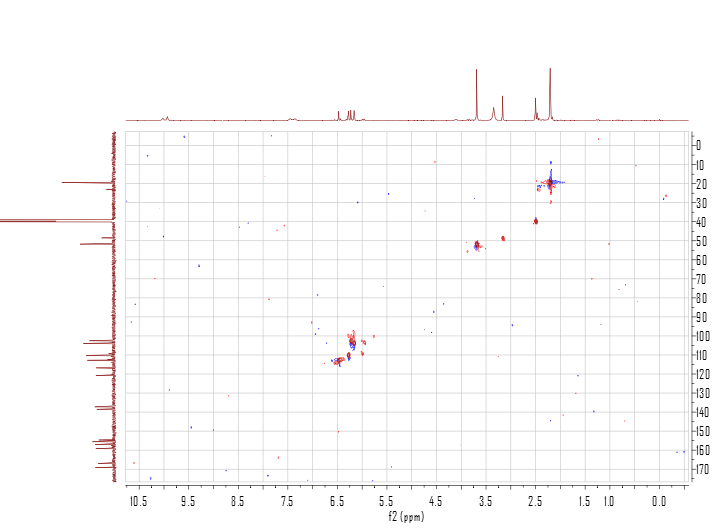


**Figure S28** HSQC spectrum of compound **4**


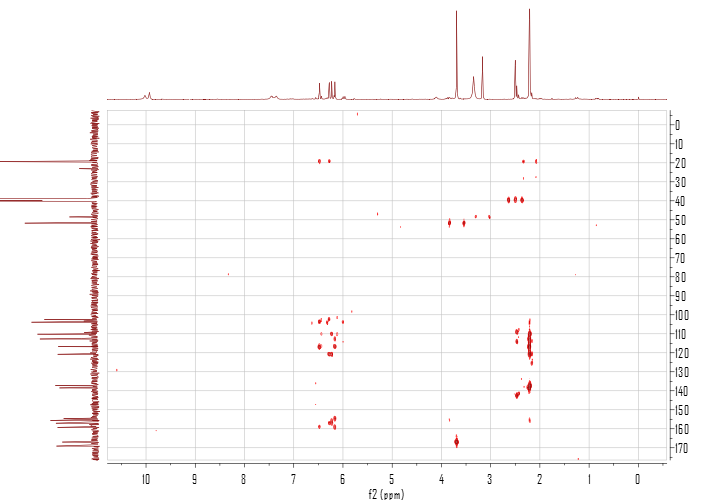


**Figure S29** HMBC spectrum of compound **4**


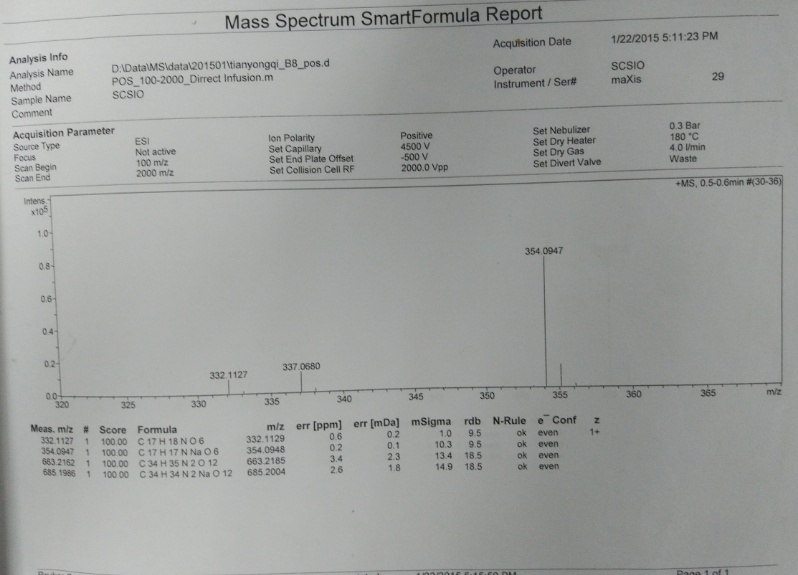


**Figure S30** HRESIMS spectrum of compound **4**


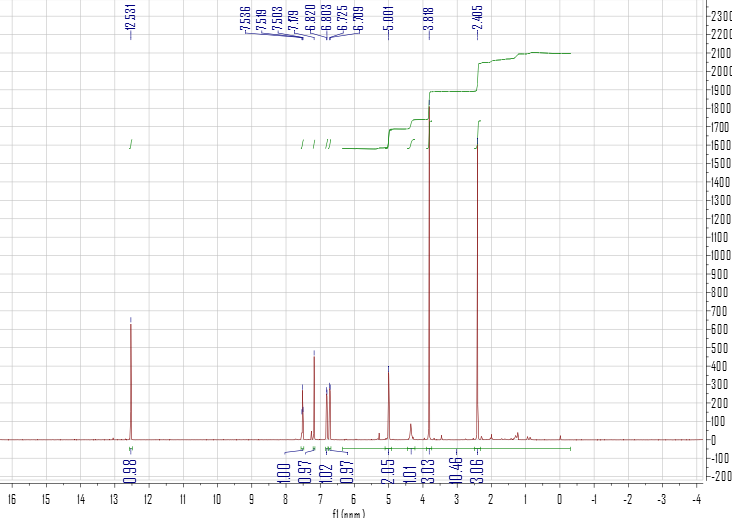


**Figure S31** ^1^H NMR spectrum of compound **5** (500 MHz, CDCl_3_)


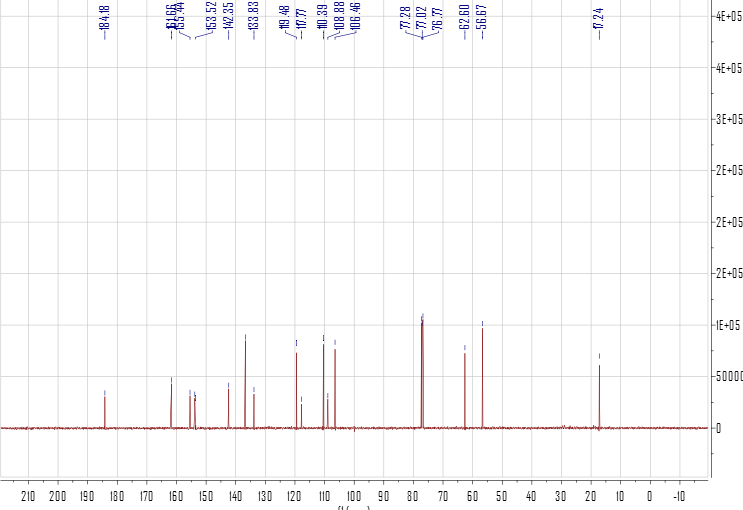


**Figure S32** ^13^C NMR spectrum of compound **5** (125 MHz, CDCl_3_)


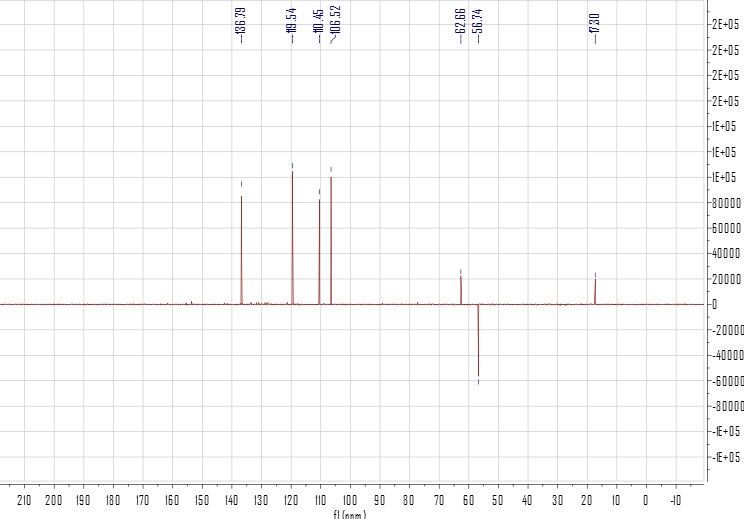


**Figure S33** DEPT-135 spectrum of compound **6** (125 MHz, CDCl_3_)


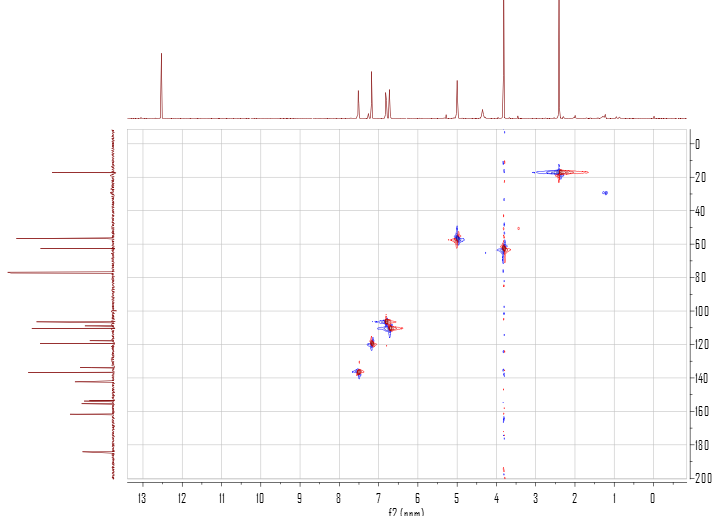


**Figure S34** HSQC spectrum of compound **7**


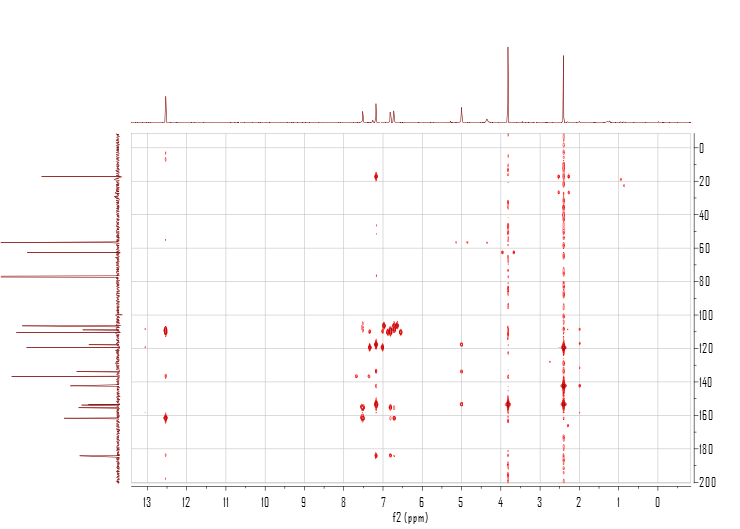


**Figure S35** HMBC spectrum of compound **5**


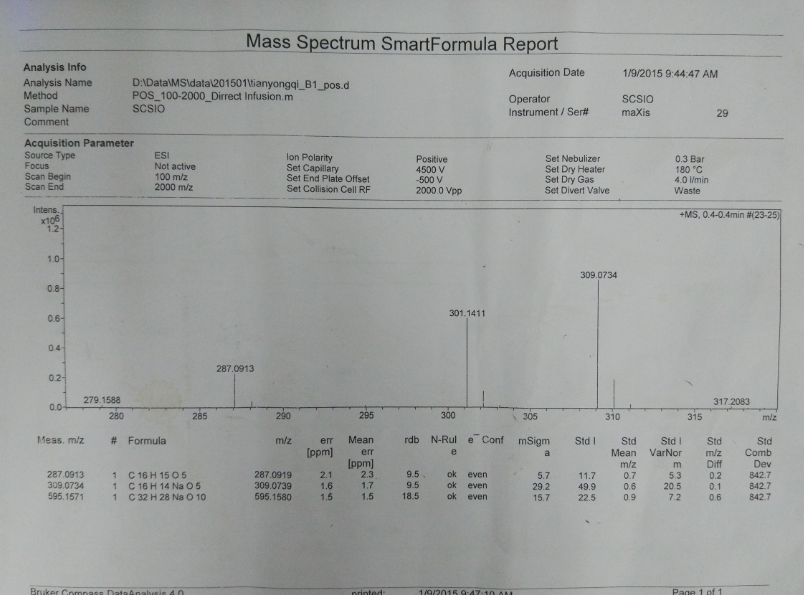


**Figure S36** HRESIMS spectrum of compound **5**


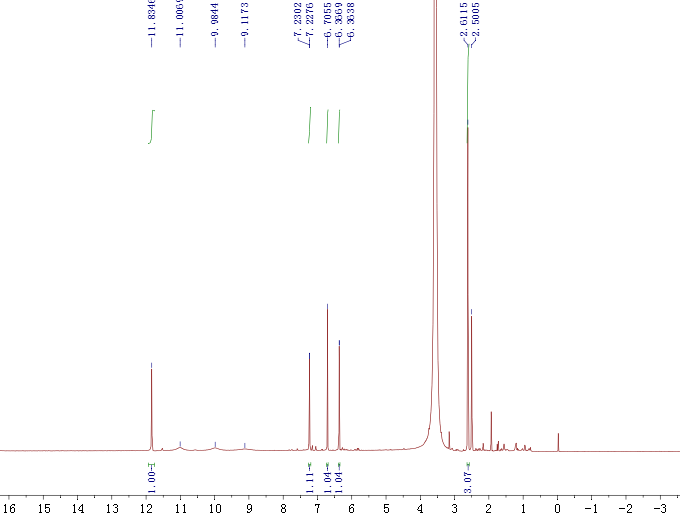


**Figure S37** ^1^H NMR spectrum of compound **6** (500 MHz, DMSO-*d*6)


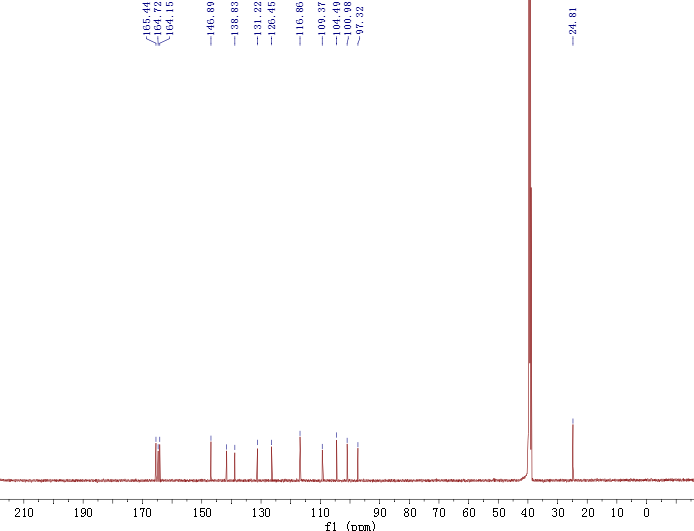


**Figure S38** ^13^C NMR spectrum of compound **6** (125 MHz, DMSO-*d*6)


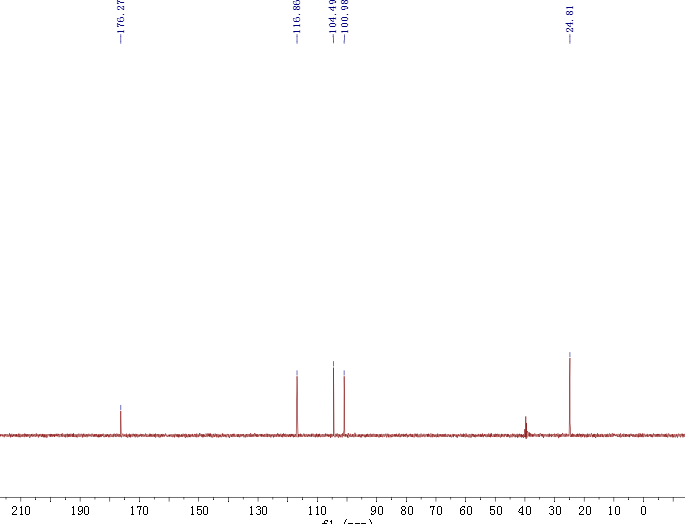


**Figure S39** DEPT-135 spectrum of compound **6** (125 MHz, DMSO-*d*6)


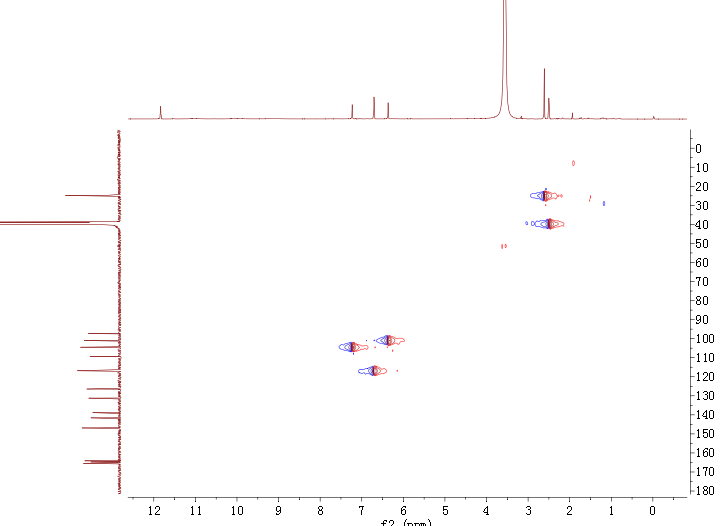


**Figure S40** HSQC spectrum of compound **6**


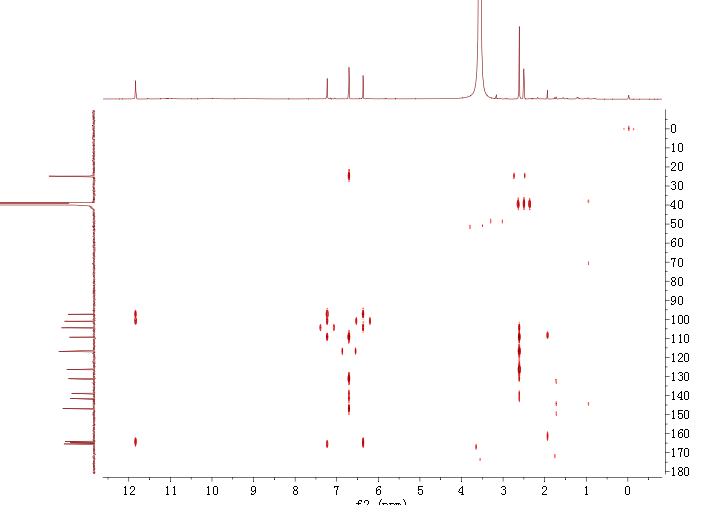


**Figure S41** HMBC spectrum of compound **6**


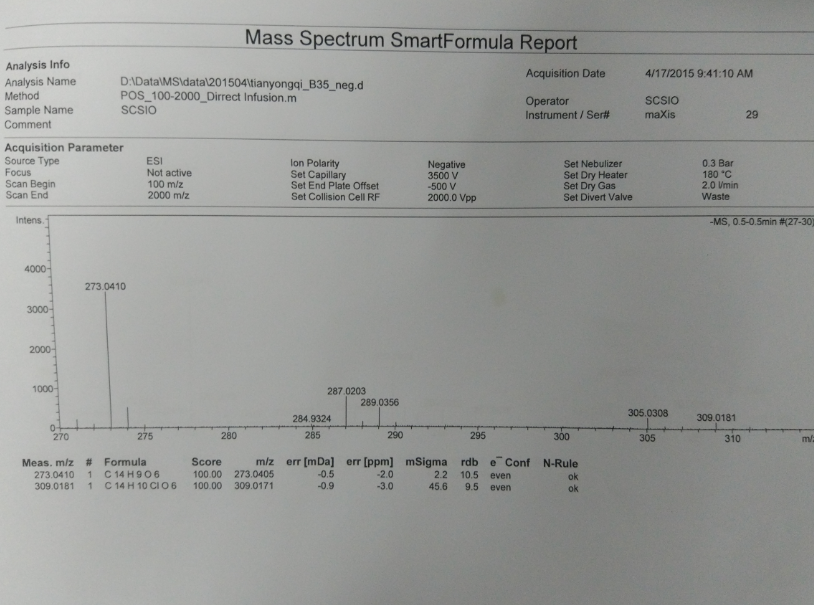


**Figure S42** HRESIMS spectrum of compound **6**


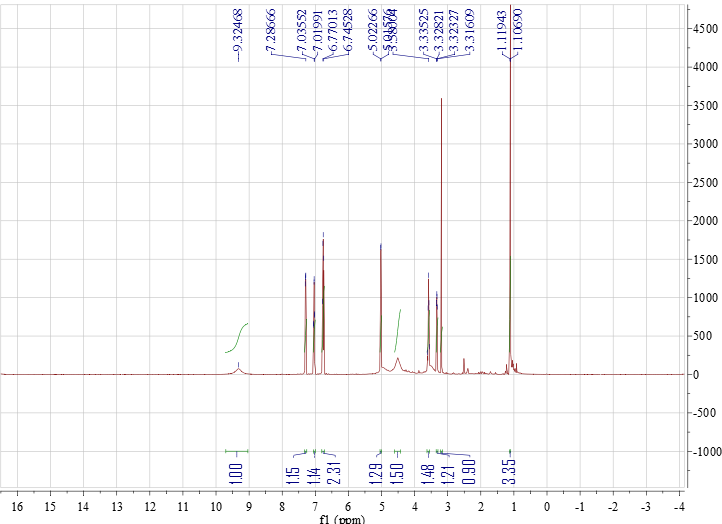


**Figure S43** ^1^H NMR spectrum of compound **7** (500 MHz, DMSO-*d*6)


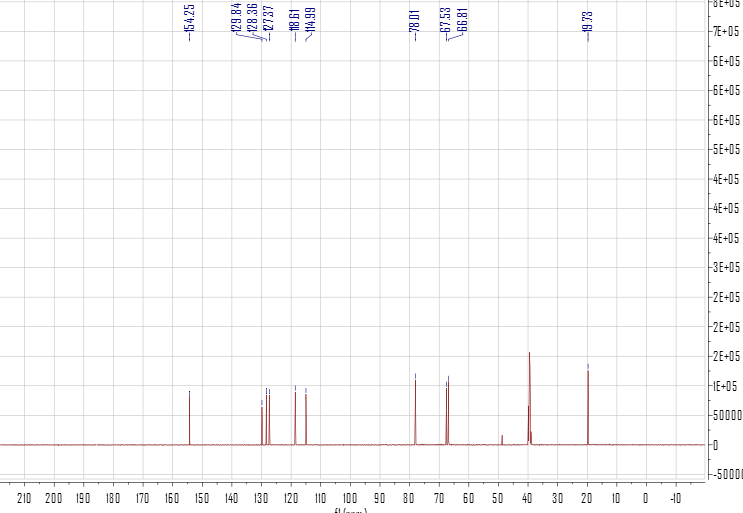


**Figure S44** ^13^C NMR spectrum of compound **7** (125 MHz, DMSO-*d*6)


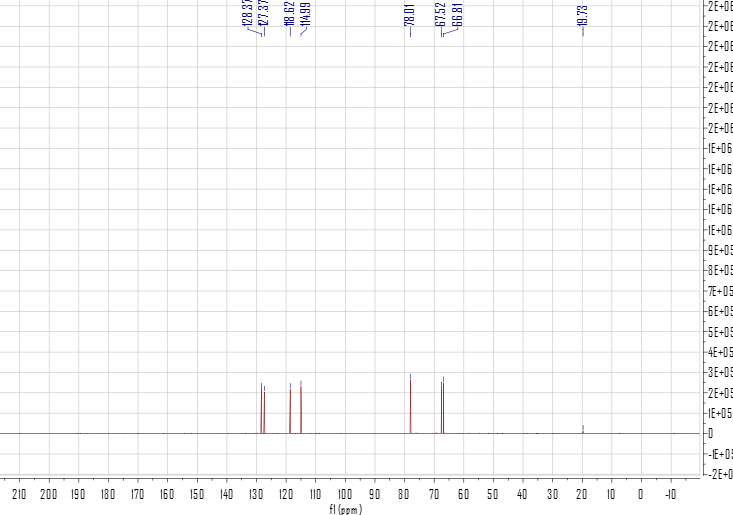


**Figure S45** DEPT-135 spectrum of compound **7** (125 MHz, DMSO-*d*6)


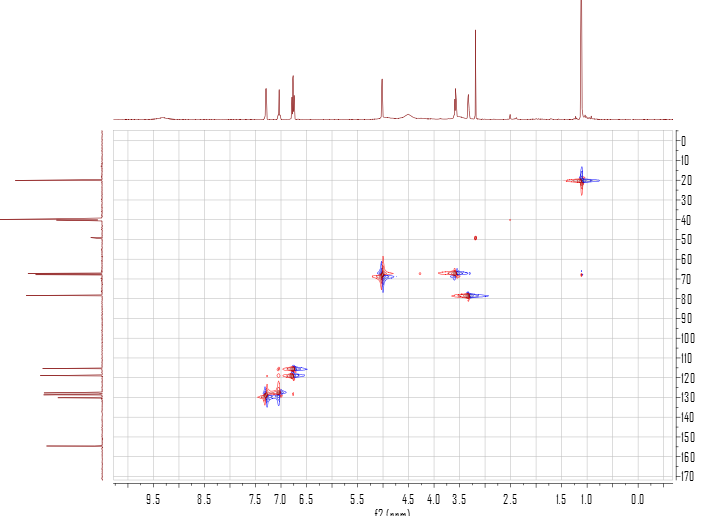


**Figure S46** HSQC spectrum of compound **7**


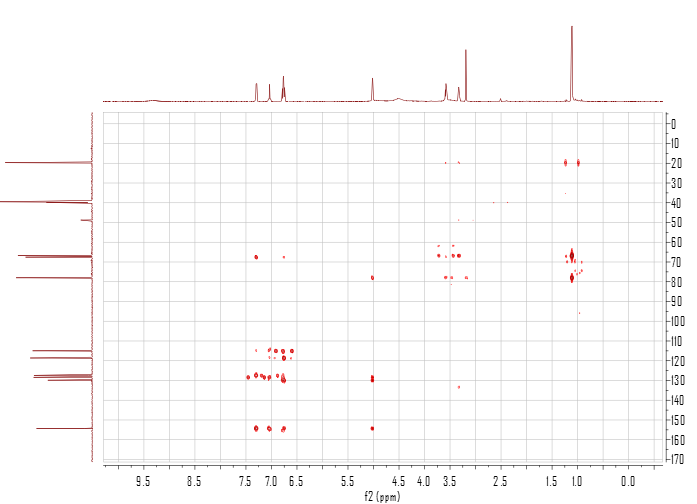


**Figure S47** HMBC spectrum of compound **7**


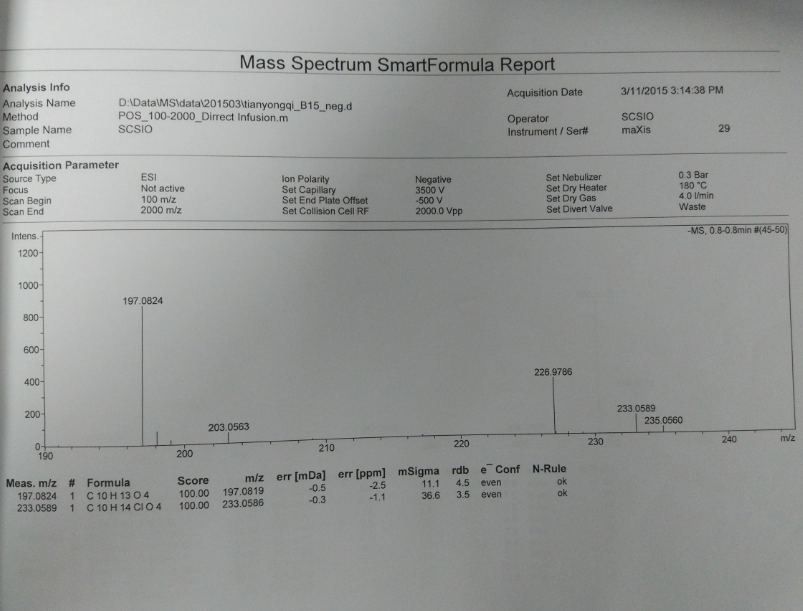


**Figure S48** HRESIMS spectrum of compound **7**

**Figure S49** Eight possible stereoisomers (**a**-**h**) of compound **1**

Systematic conformational search was performed by Confab program at MMFF94 force field. Conformers for each configuration were obtained with filtration by RMSD threshold of 2.0 Å.

**Table S1** Energies of compound **1** at MMFF94 force field.

| Configuration | Conformer | Energy (kcal/mol) | Population (%) |
| --- | --- | --- | --- |
| a | 1 | 38.82 | 45.54 |
|  | 2 | 39.20 | 23.63 |
|  | 3 | 39.26 | 21.51 |
|  | 4 | 39.82 | 8.30 |
| b | 1 | 42.65 | 39.50 |
|  | 2 | 42.93 | 24.90 |
|  | 3 | 43.01 | 21.78 |
|  | 4 | 43.38 | 11.50 |
| c | 1 | 44.27 | 35.29 |
|  | 2 | 44.44 | 26.39 |
|  | 3 | 44.60 | 20.07 |
|  | 4 | 44.70 | 17.01 |
| d | 1 | 46.01 | 39.40 |
|  | 2 | 46.23 | 26.89 |
|  | 3 | 46.43 | 19.20 |
|  | 4 | 46.68 | 12.64 |
| e | 1 | 46.01 | 40.35 |
|  | 2 | 46.16 | 31.22 |
|  | 3 | 46.47 | 18.37 |
|  | 4 | 46.92 | 8.65 |
| f | 1 | 44.44 | 35.27 |
|  | 2 | 44.48 | 32.98 |
|  | 3 | 44.77 | 20.29 |
|  | 4 | 45.25 | 8.94 |
| g | 1 | 42.65 | 38.32 |
|  | 2 | 42.75 | 32.35 |
|  | 3 | 43.11 | 17.66 |
|  | 4 | 43.52 | 8.85 |
| h | 1 | 38.82 | 46.23 |
|  | 2 | 39.19 | 24.42 |
|  | 3 | 39.47 | 15.25 |
|  | 4 | 39.70 | 10.34 |

Structures for ECD calculations were optimized at B3LYP/6-311G(d,p) in methanol and optimized at B3LYP/6-31+G(d,p) in gas phase.

**Table S2** Energies of compound **1** at B3LYP/6-311G(d,p) in methanol.

| Configuration | Conformer | Structure | E (Hartree) | E (kcal/mol) | Population (%) |
| --- | --- | --- | --- | --- | --- |
| **a** | 1 | 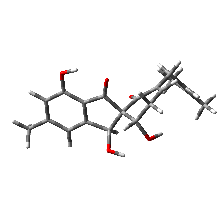 | -1151.80101303 | -722766.04 | 18.22 |
| **a** | 2 | 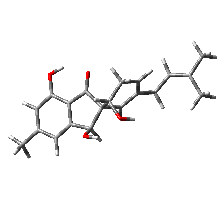 | -1151.80150795 | -722766.35 | 30.79 |
| **a** | 3 | 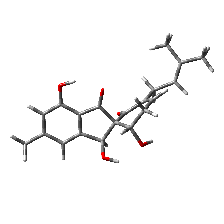 | -1151.80154509 | -722766.38 | 32.02 |
| **a** | 4 | 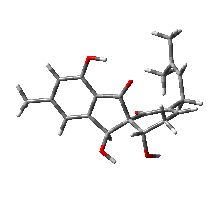 | -1151.80105119 | -722766.07 | 18.97 |
| **b** | 1 | 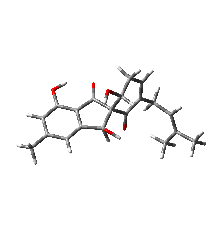 | -1151.80356528 | -722767.64 | 16.52 |
| **b** | 2 | 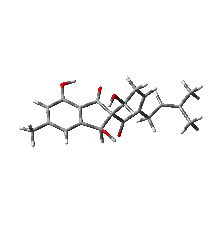 | -1151.80412029 | -722767.99 | 29.76 |
| **b** | 3 | 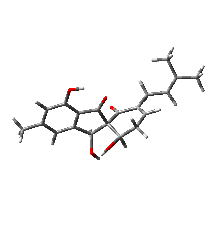 | -1151.80418957 | -722768.04 | 32.02 |
| **b** | 4 | 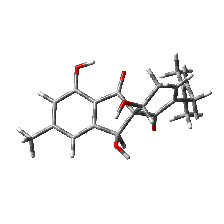 | -1151.80382230 | -722767.80 | 21.70 |
| **c** | 1 | 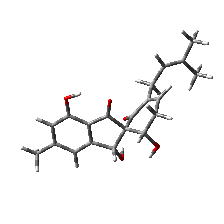 | -1151.80269722 | -722767.10 | 34.14 |
| **c** | 2 | 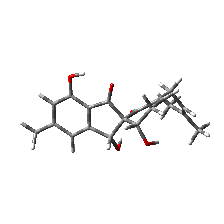 | -1151.80083476 | -722765.93 | 4.74 |
| **c** | 3 | 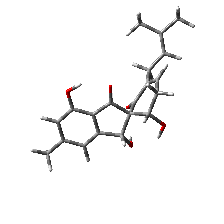 | -1151.80262762 | -722767.06 | 31.71 |
| **c** | 4 | 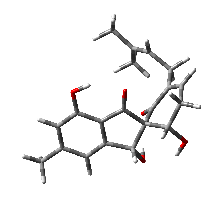 | -1151.80255619 | -722767.01 | 29.40 |
| **d** | 1 | 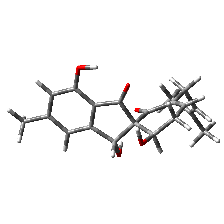 | -1151.80292104 | -722767.24 | 17.65 |
| **d** | 2 | 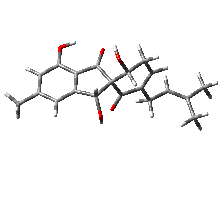 | -1151.80342311 | -722767.55 | 30.04 |
| **d** | 3 | 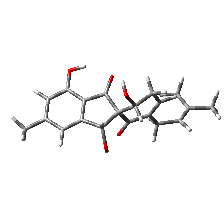 | -1151.80343752 | -722767.56 | 30.50 |
| **d** | 4 | 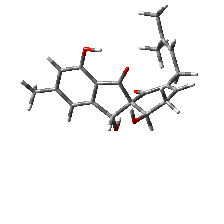 | -1151.80312081 | -722767.36 | 21.81 |
| **e** | 1 | 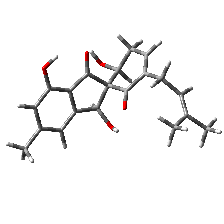 | -1151.80292104 | -722767.24 | 17.65 |
| **e** | 2 | 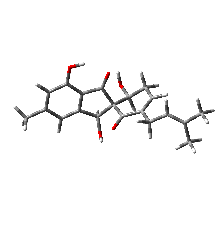 | -1151.80342311 | -722767.55 | 30.04 |
| **e** | 3 | 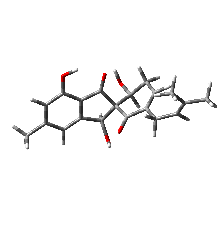 | -1151.80343752 | -722767.56 | 30.50 |
| **e** | 4 | 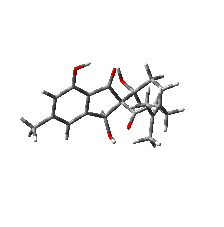 | -1151.80312080 | -722767.36 | 21.81 |
| **f** | 1 | 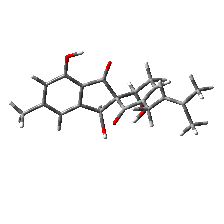 | -1151.80269722 | -722767.10 | 34.14 |
| **f** | 2 | 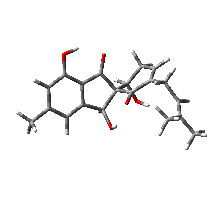 | -1151.80083476 | -722765.93 | 4.74 |
| **f** | 3 | 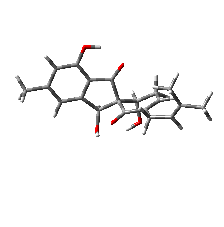 | -1151.80262752 | -722767.06 | 31.71 |
| **f** | 4 | 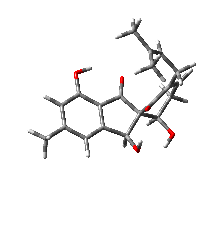 | -1151.80255619 | -722767.01 | 29.40 |
| **g** | 1 | 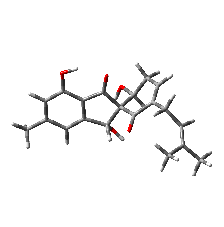 | -1151.80356528 | -722767.64 | 16.52 |
| **g** | 2 | 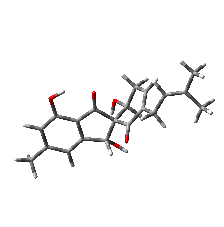 | -1151.80412029 | -722767.99 | 29.76 |
| **g** | 3 | 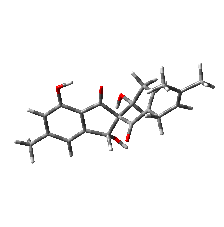 | -1151.80418957 | -722768.04 | 32.02 |
| **g** | 4 | 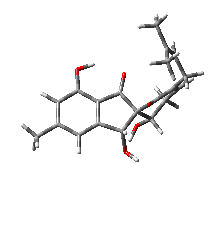 | -1151.80382230 | -722767.80 | 21.70 |
| **h** | 1 | 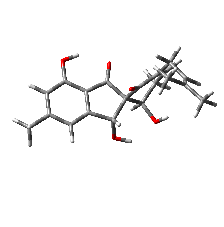 | -1151.80101303 | -722766.04 | 18.22 |
| **h** | 2 | 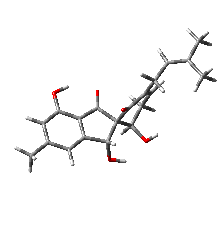 | -1151.80150795 | -722766.35 | 30.79 |
| **h** | 3 | 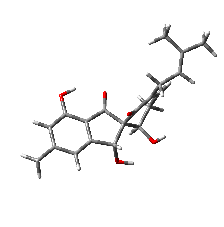 | -1151.80154509 | -722766.38 | 32.02 |
| **h** | 4 | 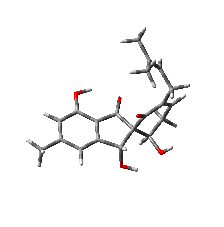 | -1151.80105119 | -722766.07 | 18.97 |

**Table S3** Energies of configurations **e** and **f** of compound **1** at B3LYP/6-31+G(d,p) in gas phase.

| Configuration | Conformer | Structure | E (Hartree) | E (kcal/mol) | Population (%) |
| --- | --- | --- | --- | --- | --- |
| **e** | 1 | 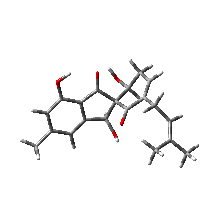 | -1151.56886485 | -722620.37 | 12.70 |
| **e** | 2 | 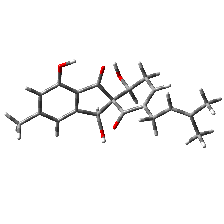 | -1151.56982782 | -722620.97 | 35.25 |
| **e** | 3 | 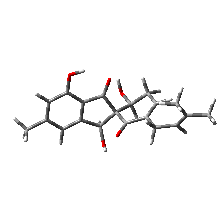 | -1151.56986271 | -722620.99 | 36.58 |
| **e** | 4 | 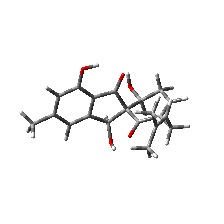 | -1151.56905090 | -722620.48 | 15.47 |
| **f** | 1 | 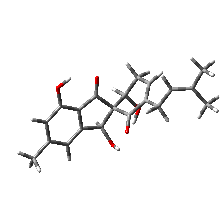 | -1151.56907327 | -722620.50 | 37.78 |
| **f** | 2 | 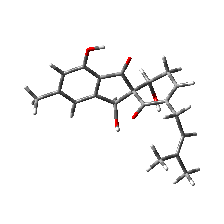 | -1151.56652537 | -722618.90 | 2.54 |
| **f** | 3 | 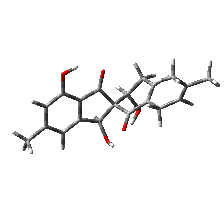 | -1151.56909578 | -722620.51 | 38.70 |
| **f** | 4 | 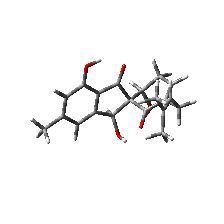 | -1151.56851814 | -722620.15 | 20.98 |

**Table S4** Standard orientations of configurations of compound **1** at B3LYP/6-311G(d,p) level in methanol for ECD calculations.

| **Conformer a-1** | | |  |  |  |  |
| --- | --- | --- | --- | --- | --- | --- |
| Center | Atomic | Atomic |  | Coordinates (Angstroms) | | |
| Number | Number | Type |  | X | Y | Z |
| 1 | 6 | 0 |  | 4.382480 | 0.855910 | -0.927190 |
| 2 | 1 | 0 |  | 5.250750 | 0.990380 | -1.561760 |
| 3 | 6 | 0 |  | 4.253300 | 1.587920 | 0.260650 |
| 4 | 6 | 0 |  | 3.128270 | 1.411260 | 1.093070 |
| 5 | 1 | 0 |  | 3.045060 | 1.972170 | 2.016770 |
| 6 | 6 | 0 |  | 2.151180 | 0.512990 | 0.710060 |
| 7 | 6 | 0 |  | 2.284730 | -0.212550 | -0.474270 |
| 8 | 6 | 0 |  | 3.400800 | -0.057780 | -1.311650 |
| 9 | 6 | 0 |  | 0.889700 | 0.107220 | 1.442580 |
| 10 | 1 | 0 |  | 0.263870 | 0.979110 | 1.649550 |
| 11 | 6 | 0 |  | 0.131910 | -0.852430 | 0.442960 |
| 12 | 6 | 0 |  | 1.162990 | -1.095000 | -0.695710 |
| 13 | 6 | 0 |  | -0.327240 | -2.168900 | 1.112710 |
| 14 | 1 | 0 |  | 0.549360 | -2.719260 | 1.451930 |
| 15 | 6 | 0 |  | -1.162070 | -3.032620 | 0.172200 |
| 16 | 1 | 0 |  | -0.513201 | -3.512040 | -0.567710 |
| 17 | 1 | 0 |  | -1.623361 | -3.836830 | 0.752800 |
| 18 | 6 | 0 |  | -2.216570 | -2.245500 | -0.543490 |
| 19 | 1 | 0 |  | -3.046640 | -2.810850 | -0.959820 |
| 20 | 6 | 0 |  | -2.201730 | -0.909830 | -0.708050 |
| 21 | 6 | 0 |  | -1.053820 | -0.111270 | -0.220320 |
| 22 | 8 | 0 |  | 1.039430 | -1.872750 | -1.636860 |
| 23 | 8 | 0 |  | -0.994770 | 1.096650 | -0.380720 |
| 24 | 8 | 0 |  | 1.278430 | -0.528630 | 2.655170 |
| 25 | 1 | 0 |  | 0.458430 | -0.849000 | 3.062160 |
| 26 | 8 | 0 |  | -1.050070 | -1.878500 | 2.324920 |
| 27 | 1 | 0 |  | -1.912750 | -1.509690 | 2.096980 |
| 28 | 6 | 0 |  | -3.324980 | -0.159230 | -1.397810 |
| 29 | 1 | 0 |  | -2.898310 | 0.612770 | -2.038100 |
| 30 | 1 | 0 |  | -3.853180 | -0.866490 | -2.046750 |
| 31 | 6 | 0 |  | -4.308200 | 0.429950 | -0.412460 |
| 32 | 1 | 0 |  | -4.798730 | -0.310480 | 0.218200 |
| 33 | 6 | 0 |  | -4.642350 | 1.714120 | -0.234480 |
| 34 | 6 | 0 |  | -5.669630 | 2.103110 | 0.801580 |
| 35 | 1 | 0 |  | -6.511810 | 2.630080 | 0.337890 |
| 36 | 1 | 0 |  | -5.239390 | 2.792560 | 1.537570 |
| 37 | 1 | 0 |  | -6.061080 | 1.234060 | 1.334010 |
| 38 | 6 | 0 |  | -4.067110 | 2.873040 | -1.007400 |
| 39 | 1 | 0 |  | -4.860620 | 3.404790 | -1.545210 |
| 40 | 1 | 0 |  | -3.304580 | 2.577270 | -1.725750 |
| 41 | 1 | 0 |  | -3.616830 | 3.598720 | -0.320520 |
| 42 | 8 | 0 |  | 3.528040 | -0.766990 | -2.451410 |
| 43 | 1 | 0 |  | 2.749350 | -1.351100 | -2.527030 |
| 44 | 6 | 0 |  | 5.315800 | 2.585670 | 0.646620 |
| 45 | 1 | 0 |  | 4.960860 | 3.606760 | 0.471900 |
| 46 | 1 | 0 |  | 6.229460 | 2.445160 | 0.067880 |
| 47 | 1 | 0 |  | 5.560160 | 2.507220 | 1.708690 |
| **Conformer a-2** | | |  |  |  |  |
| Center | Atomic | Atomic |  | Coordinates (Angstroms) | | |
| Number | Number | Type |  | X | Y | Z |
| 1 | 6 | 0 |  | 4.567729 | -1.470582 | -0.099670 |
| 2 | 1 | 0 |  | 5.311929 | -2.246542 | 0.036970 |
| 3 | 6 | 0 |  | 4.838110 | -0.365782 | -0.917220 |
| 4 | 6 | 0 |  | 3.871350 | 0.646319 | -1.097210 |
| 5 | 1 | 0 |  | 4.093230 | 1.501849 | -1.724790 |
| 6 | 6 | 0 |  | 2.652140 | 0.523329 | -0.460370 |
| 7 | 6 | 0 |  | 2.388799 | -0.578951 | 0.354050 |
| 8 | 6 | 0 |  | 3.339659 | -1.592201 | 0.552260 |
| 9 | 6 | 0 |  | 1.478590 | 1.480200 | -0.455180 |
| 10 | 1 | 0 |  | 1.135120 | 1.675920 | -1.474030 |
| 11 | 6 | 0 |  | 0.342580 | 0.723930 | 0.339270 |
| 12 | 6 | 0 |  | 1.061459 | -0.526750 | 0.920740 |
| 13 | 6 | 0 |  | -0.302210 | 1.599741 | 1.440310 |
| 14 | 1 | 0 |  | 0.457831 | 1.862820 | 2.174950 |
| 15 | 6 | 0 |  | -1.474840 | 0.900111 | 2.119310 |
| 16 | 1 | 0 |  | -1.102710 | 0.130061 | 2.802630 |
| 17 | 1 | 0 |  | -2.009970 | 1.631112 | 2.732310 |
| 18 | 6 | 0 |  | -2.413400 | 0.263592 | 1.139950 |
| 19 | 1 | 0 |  | -3.419270 | 0.053532 | 1.491500 |
| 20 | 6 | 0 |  | -2.094740 | -0.066198 | -0.124550 |
| 21 | 6 | 0 |  | -0.726080 | 0.176161 | -0.635780 |
| 22 | 8 | 0 |  | 0.582609 | -1.347030 | 1.697470 |
| 23 | 8 | 0 |  | -0.420090 | -0.084949 | -1.788150 |
| 24 | 8 | 0 |  | 1.903581 | 2.686690 | 0.169310 |
| 25 | 1 | 0 |  | 1.113531 | 3.245530 | 0.233230 |
| 26 | 8 | 0 |  | -0.707869 | 2.865111 | 0.883880 |
| 27 | 1 | 0 |  | -1.487959 | 2.729571 | 0.331240 |
| 28 | 6 | 0 |  | -3.067511 | -0.691718 | -1.110930 |
| 29 | 1 | 0 |  | -3.271570 | 0.037312 | -1.900130 |
| 30 | 1 | 0 |  | -2.540381 | -1.511058 | -1.613280 |
| 31 | 6 | 0 |  | -4.336211 | -1.221557 | -0.502930 |
| 32 | 1 | 0 |  | -4.204811 | -2.102577 | 0.123700 |
| 33 | 6 | 0 |  | -5.581841 | -0.752047 | -0.651320 |
| 34 | 6 | 0 |  | -6.750361 | -1.440486 | 0.011500 |
| 35 | 1 | 0 |  | -7.482451 | -1.769346 | -0.735330 |
| 36 | 1 | 0 |  | -7.278651 | -0.753746 | 0.682930 |
| 37 | 1 | 0 |  | -6.435461 | -2.311586 | 0.589210 |
| 38 | 6 | 0 |  | -5.953340 | 0.461163 | -1.466090 |
| 39 | 1 | 0 |  | -6.638840 | 0.184454 | -2.275360 |
| 40 | 1 | 0 |  | -5.094690 | 0.965933 | -1.907900 |
| 41 | 1 | 0 |  | -6.487540 | 1.186904 | -0.842560 |
| 42 | 8 | 0 |  | 3.082728 | -2.653021 | 1.343700 |
| 43 | 1 | 0 |  | 2.183708 | -2.542080 | 1.707990 |
| 44 | 6 | 0 |  | 6.173230 | -0.248332 | -1.607910 |
| 45 | 1 | 0 |  | 6.042470 | -0.153192 | -2.689640 |
| 46 | 1 | 0 |  | 6.805399 | -1.115133 | -1.413240 |
| 47 | 1 | 0 |  | 6.704740 | 0.646197 | -1.269500 |
| **Conformer a-3** | | |  |  |  |  |
| Center | Atomic | Atomic |  | Coordinates (Angstroms) | | |
| Number | Number | Type |  | X | Y | Z |
| 1 | 6 | 0 |  | 4.362760 | -1.719241 | 0.201820 |
| 2 | 1 | 0 |  | 5.022030 | -2.538411 | 0.464390 |
| 3 | 6 | 0 |  | 4.776450 | -0.743121 | -0.714450 |
| 4 | 6 | 0 |  | 3.924100 | 0.328259 | -1.054380 |
| 5 | 1 | 0 |  | 4.258930 | 1.085789 | -1.753530 |
| 6 | 6 | 0 |  | 2.670850 | 0.391339 | -0.476560 |
| 7 | 6 | 0 |  | 2.264480 | -0.583231 | 0.435450 |
| 8 | 6 | 0 |  | 3.100890 | -1.652641 | 0.793060 |
| 9 | 6 | 0 |  | 1.601660 | 1.451779 | -0.633520 |
| 10 | 1 | 0 |  | 1.317760 | 1.563499 | -1.683080 |
| 11 | 6 | 0 |  | 0.365250 | 0.905499 | 0.183510 |
| 12 | 6 | 0 |  | 0.928740 | -0.339001 | 0.927200 |
| 13 | 6 | 0 |  | -0.220089 | 1.959110 | 1.153500 |
| 14 | 1 | 0 |  | 0.538911 | 2.225559 | 1.887940 |
| 15 | 6 | 0 |  | -1.481420 | 1.460660 | 1.851750 |
| 16 | 1 | 0 |  | -1.214110 | 0.736110 | 2.627700 |
| 17 | 1 | 0 |  | -1.956399 | 2.304810 | 2.360070 |
| 18 | 6 | 0 |  | -2.448520 | 0.817720 | 0.904690 |
| 19 | 1 | 0 |  | -3.479930 | 0.738900 | 1.235660 |
| 20 | 6 | 0 |  | -2.125310 | 0.326180 | -0.304930 |
| 21 | 6 | 0 |  | -0.720870 | 0.362530 | -0.773750 |
| 22 | 8 | 0 |  | 0.338240 | -1.018031 | 1.761320 |
| 23 | 8 | 0 |  | -0.402790 | -0.067620 | -1.870890 |
| 24 | 8 | 0 |  | 2.124831 | 2.673479 | -0.124010 |
| 25 | 1 | 0 |  | 1.394891 | 3.311089 | -0.159480 |
| 26 | 8 | 0 |  | -0.473109 | 3.189550 | 0.447530 |
| 27 | 1 | 0 |  | -1.242119 | 3.072230 | -0.124490 |
| 28 | 6 | 0 |  | -3.120050 | -0.325540 | -1.250990 |
| 29 | 1 | 0 |  | -2.927380 | 0.076100 | -2.252550 |
| 30 | 1 | 0 |  | -2.876730 | -1.389420 | -1.322890 |
| 31 | 6 | 0 |  | -4.565650 | -0.107970 | -0.901380 |
| 32 | 1 | 0 |  | -4.910450 | 0.918660 | -1.017540 |
| 33 | 6 | 0 |  | -5.460600 | -1.013840 | -0.485810 |
| 34 | 6 | 0 |  | -6.890960 | -0.615640 | -0.213790 |
| 35 | 1 | 0 |  | -7.168940 | -0.840790 | 0.822380 |
| 36 | 1 | 0 |  | -7.580630 | -1.181990 | -0.850540 |
| 37 | 1 | 0 |  | -7.057450 | 0.448700 | -0.390480 |
| 38 | 6 | 0 |  | -5.165150 | -2.474220 | -0.254760 |
| 39 | 1 | 0 |  | -5.417900 | -2.753600 | 0.774340 |
| 40 | 1 | 0 |  | -4.122230 | -2.738540 | -0.427160 |
| 41 | 1 | 0 |  | -5.786620 | -3.098790 | -0.906840 |
| 42 | 8 | 0 |  | 2.705590 | -2.587941 | 1.680210 |
| 43 | 1 | 0 |  | 1.806830 | -2.352541 | 1.980300 |
| 44 | 6 | 0 |  | 6.137060 | -0.844751 | -1.356160 |
| 45 | 1 | 0 |  | 6.044390 | -1.175791 | -2.395830 |
| 46 | 1 | 0 |  | 6.776430 | -1.557111 | -0.833500 |
| 47 | 1 | 0 |  | 6.636870 | 0.126759 | -1.371080 |
| **Conformer a-4** | | |  |  |  |  |
| Center | Atomic | Atomic |  | Coordinates (Angstroms) | | |
| Number | Number | Type |  | X | Y | Z |
| 1 | 6 | 0 |  | 3.621182 | -1.852868 | 0.700630 |
| 2 | 1 | 0 |  | 4.168122 | -2.641178 | 1.204620 |
| 3 | 6 | 0 |  | 4.056432 | -1.371378 | -0.540740 |
| 4 | 6 | 0 |  | 3.346661 | -0.345798 | -1.200650 |
| 5 | 1 | 0 |  | 3.696121 | 0.026032 | -2.157050 |
| 6 | 6 | 0 |  | 2.212591 | 0.169051 | -0.604160 |
| 7 | 6 | 0 |  | 1.785161 | -0.312669 | 0.634130 |
| 8 | 6 | 0 |  | 2.480382 | -1.327679 | 1.309430 |
| 9 | 6 | 0 |  | 1.313160 | 1.292230 | -1.075670 |
| 10 | 1 | 0 |  | 0.917861 | 1.080730 | -2.072170 |
| 11 | 6 | 0 |  | 0.123490 | 1.320110 | -0.039350 |
| 12 | 6 | 0 |  | 0.582831 | 0.349120 | 1.084850 |
| 13 | 6 | 0 |  | -0.170190 | 2.744820 | 0.488920 |
| 14 | 1 | 0 |  | 0.708870 | 3.113390 | 1.016220 |
| 15 | 6 | 0 |  | -1.390120 | 2.786469 | 1.403760 |
| 16 | 1 | 0 |  | -1.128600 | 2.386989 | 2.389180 |
| 17 | 1 | 0 |  | -1.676331 | 3.831179 | 1.555870 |
| 18 | 6 | 0 |  | -2.548500 | 2.004489 | 0.864830 |
| 19 | 1 | 0 |  | -3.526000 | 2.239828 | 1.278490 |
| 20 | 6 | 0 |  | -2.460119 | 1.037299 | -0.066230 |
| 21 | 6 | 0 |  | -1.145959 | 0.677589 | -0.647480 |
| 22 | 8 | 0 |  | 0.012961 | 0.149450 | 2.152930 |
| 23 | 8 | 0 |  | -1.044119 | -0.128291 | -1.557320 |
| 24 | 8 | 0 |  | 2.084540 | 2.489361 | -1.087240 |
| 25 | 1 | 0 |  | 1.467290 | 3.200101 | -1.320080 |
| 26 | 8 | 0 |  | -0.320441 | 3.655140 | -0.617650 |
| 27 | 1 | 0 |  | -1.160331 | 3.480309 | -1.060550 |
| 28 | 6 | 0 |  | -3.664059 | 0.248508 | -0.544840 |
| 29 | 1 | 0 |  | -4.561519 | 0.842918 | -0.342310 |
| 30 | 1 | 0 |  | -3.599709 | 0.116788 | -1.624970 |
| 31 | 6 | 0 |  | -3.785158 | -1.082142 | 0.162260 |
| 32 | 1 | 0 |  | -3.914388 | -0.999452 | 1.240570 |
| 33 | 6 | 0 |  | -3.754148 | -2.312202 | -0.365500 |
| 34 | 6 | 0 |  | -3.908267 | -3.529192 | 0.514840 |
| 35 | 1 | 0 |  | -3.028387 | -4.178832 | 0.438970 |
| 36 | 1 | 0 |  | -4.767817 | -4.132903 | 0.200450 |
| 37 | 1 | 0 |  | -4.045287 | -3.259042 | 1.563740 |
| 38 | 6 | 0 |  | -3.574218 | -2.620522 | -1.829800 |
| 39 | 1 | 0 |  | -2.723747 | -3.297192 | -1.970550 |
| 40 | 1 | 0 |  | -3.398718 | -1.734992 | -2.437940 |
| 41 | 1 | 0 |  | -4.455017 | -3.142482 | -2.221870 |
| 42 | 8 | 0 |  | 2.065912 | -1.787749 | 2.507150 |
| 43 | 1 | 0 |  | 1.267102 | -1.286110 | 2.758890 |
| 44 | 6 | 0 |  | 5.297302 | -1.944328 | -1.177610 |
| 45 | 1 | 0 |  | 5.078762 | -2.324478 | -2.179560 |
| 46 | 1 | 0 |  | 5.715372 | -2.758037 | -0.584340 |
| 47 | 1 | 0 |  | 6.064802 | -1.172287 | -1.288280 |
| **Conformer b-1** | | |  |  |  |  |
| Center | Atomic | Atomic |  | Coordinates (Angstroms) | | |
| Number | Number | Type |  | X | Y | Z |
| 1 | 6 | 0 |  | -4.481839 | -0.573460 | -1.065790 |
| 2 | 1 | 0 |  | -5.372289 | -0.502651 | -1.679600 |
| 3 | 6 | 0 |  | -4.433519 | -1.469560 | 0.011050 |
| 4 | 6 | 0 |  | -3.274329 | -1.571390 | 0.807260 |
| 5 | 1 | 0 |  | -3.242749 | -2.270200 | 1.634890 |
| 6 | 6 | 0 |  | -2.190029 | -0.768050 | 0.507610 |
| 7 | 6 | 0 |  | -2.249510 | 0.133880 | -0.553900 |
| 8 | 6 | 0 |  | -3.390170 | 0.242130 | -1.364250 |
| 9 | 6 | 0 |  | -0.835179 | -0.706450 | 1.177360 |
| 10 | 1 | 0 |  | -0.287569 | -1.631900 | 0.989120 |
| 11 | 6 | 0 |  | -0.073280 | 0.477390 | 0.470060 |
| 12 | 6 | 0 |  | -1.036100 | 0.908930 | -0.669250 |
| 13 | 6 | 0 |  | 0.196420 | 1.652740 | 1.448560 |
| 14 | 1 | 0 |  | 0.820660 | 1.250730 | 2.256450 |
| 15 | 6 | 0 |  | 0.961140 | 2.783980 | 0.778890 |
| 16 | 1 | 0 |  | 0.289090 | 3.343190 | 0.118390 |
| 17 | 1 | 0 |  | 1.300100 | 3.489480 | 1.543640 |
| 18 | 6 | 0 |  | 2.130510 | 2.285260 | -0.008020 |
| 19 | 1 | 0 |  | 2.897680 | 3.016671 | -0.249790 |
| 20 | 6 | 0 |  | 2.298250 | 1.017380 | -0.425740 |
| 21 | 6 | 0 |  | 1.248150 | 0.005680 | -0.177670 |
| 22 | 8 | 0 |  | -0.812950 | 1.757300 | -1.526970 |
| 23 | 8 | 0 |  | 1.378891 | -1.158390 | -0.522320 |
| 24 | 8 | 0 |  | -1.016169 | -0.530720 | 2.587100 |
| 25 | 1 | 0 |  | -0.257469 | -0.905930 | 3.047410 |
| 26 | 8 | 0 |  | -1.008550 | 2.177480 | 1.985620 |
| 27 | 1 | 0 |  | -1.357810 | 1.480700 | 2.559960 |
| 28 | 6 | 0 |  | 3.542340 | 0.559201 | -1.163430 |
| 29 | 1 | 0 |  | 3.255050 | -0.137939 | -1.950490 |
| 30 | 1 | 0 |  | 3.988230 | 1.434261 | -1.649480 |
| 31 | 6 | 0 |  | 4.563630 | -0.057099 | -0.234950 |
| 32 | 1 | 0 |  | 4.908310 | 0.614791 | 0.550140 |
| 33 | 6 | 0 |  | 5.083971 | -1.290149 | -0.267300 |
| 34 | 6 | 0 |  | 6.116911 | -1.715579 | 0.748690 |
| 35 | 1 | 0 |  | 7.047981 | -2.019229 | 0.255770 |
| 36 | 1 | 0 |  | 5.768941 | -2.585869 | 1.317580 |
| 37 | 1 | 0 |  | 6.349051 | -0.915469 | 1.454440 |
| 38 | 6 | 0 |  | 4.723541 | -2.354449 | -1.271970 |
| 39 | 1 | 0 |  | 5.607511 | -2.651709 | -1.848290 |
| 40 | 1 | 0 |  | 3.948131 | -2.043839 | -1.969940 |
| 41 | 1 | 0 |  | 4.370921 | -3.255539 | -0.757270 |
| 42 | 8 | 0 |  | -3.437670 | 1.110010 | -2.395170 |
| 43 | 1 | 0 |  | -2.582550 | 1.581010 | -2.425270 |
| 44 | 6 | 0 |  | -5.638309 | -2.316861 | 0.334210 |
| 45 | 1 | 0 |  | -5.343139 | -3.329220 | 0.619120 |
| 46 | 1 | 0 |  | -6.324729 | -2.378451 | -0.511340 |
| 47 | 1 | 0 |  | -6.187359 | -1.889471 | 1.179990 |
| **Conformer b-2** | | |  |  |  |  |
| Center | Atomic | Atomic |  | Coordinates (Angstroms) | | |
| Number | Number | Type |  | X | Y | Z |
| 1 | 6 | 0 |  | -4.599580 | 1.387170 | -0.300980 |
| 2 | 1 | 0 |  | -5.356630 | 2.161670 | -0.256310 |
| 3 | 6 | 0 |  | -4.935260 | 0.092490 | -0.722830 |
| 4 | 6 | 0 |  | -3.953160 | -0.915710 | -0.791700 |
| 5 | 1 | 0 |  | -4.219170 | -1.912100 | -1.124020 |
| 6 | 6 | 0 |  | -2.654700 | -0.604440 | -0.431570 |
| 7 | 6 | 0 |  | -2.329910 | 0.678940 | 0.004000 |
| 8 | 6 | 0 |  | -3.292180 | 1.699240 | 0.069680 |
| 9 | 6 | 0 |  | -1.424660 | -1.483850 | -0.448270 |
| 10 | 1 | 0 |  | -1.150340 | -1.718130 | -1.478700 |
| 11 | 6 | 0 |  | -0.278500 | -0.609410 | 0.185890 |
| 12 | 6 | 0 |  | -0.937890 | 0.783540 | 0.376520 |
| 13 | 6 | 0 |  | 0.201990 | -1.192200 | 1.543390 |
| 14 | 1 | 0 |  | 0.586900 | -2.197780 | 1.332750 |
| 15 | 6 | 0 |  | 1.330740 | -0.370410 | 2.146730 |
| 16 | 1 | 0 |  | 0.925160 | 0.556310 | 2.568100 |
| 17 | 1 | 0 |  | 1.769160 | -0.923780 | 2.982660 |
| 18 | 6 | 0 |  | 2.390060 | -0.034350 | 1.145480 |
| 19 | 1 | 0 |  | 3.358080 | 0.257970 | 1.541660 |
| 20 | 6 | 0 |  | 2.229490 | -0.071250 | -0.188960 |
| 21 | 6 | 0 |  | 0.921700 | -0.444440 | -0.770900 |
| 22 | 8 | 0 |  | -0.382110 | 1.803400 | 0.771150 |
| 23 | 8 | 0 |  | 0.773160 | -0.577790 | -1.976060 |
| 24 | 8 | 0 |  | -1.703690 | -2.695150 | 0.263380 |
| 25 | 1 | 0 |  | -1.140490 | -3.396100 | -0.082780 |
| 26 | 8 | 0 |  | -0.855680 | -1.269580 | 2.487190 |
| 27 | 1 | 0 |  | -1.452260 | -1.952620 | 2.148170 |
| 28 | 6 | 0 |  | 3.329960 | 0.246630 | -1.187900 |
| 29 | 1 | 0 |  | 3.582640 | -0.671500 | -1.725510 |
| 30 | 1 | 0 |  | 2.898120 | 0.908020 | -1.948770 |
| 31 | 6 | 0 |  | 4.549350 | 0.902640 | -0.602980 |
| 32 | 1 | 0 |  | 4.383970 | 1.911630 | -0.227480 |
| 33 | 6 | 0 |  | 5.789790 | 0.406420 | -0.507140 |
| 34 | 6 | 0 |  | 6.907880 | 1.232280 | 0.081390 |
| 35 | 1 | 0 |  | 7.714750 | 1.371530 | -0.647760 |
| 36 | 1 | 0 |  | 7.354350 | 0.726850 | 0.945570 |
| 37 | 1 | 0 |  | 6.561750 | 2.217200 | 0.400670 |
| 38 | 6 | 0 |  | 6.204200 | -0.970480 | -0.961260 |
| 39 | 1 | 0 |  | 6.971680 | -0.902160 | -1.740980 |
| 40 | 1 | 0 |  | 5.378230 | -1.564520 | -1.351270 |
| 41 | 1 | 0 |  | 6.655690 | -1.524080 | -0.130220 |
| 42 | 8 | 0 |  | -2.968950 | 2.941200 | 0.484270 |
| 43 | 1 | 0 |  | -2.015960 | 2.943590 | 0.697730 |
| 44 | 6 | 0 |  | -6.364850 | -0.226840 | -1.080890 |
| 45 | 1 | 0 |  | -6.417520 | -0.996040 | -1.853700 |
| 46 | 1 | 0 |  | -6.897170 | 0.658900 | -1.431610 |
| 47 | 1 | 0 |  | -6.899580 | -0.607300 | -0.203800 |
| **Conformer b-3** | | |  |  |  |  |
| Center | Atomic | Atomic |  | Coordinates (Angstroms) | | |
| Number | Number | Type |  | X | Y | Z |
| 1 | 6 | 0 |  | -4.371680 | 1.697749 | 0.130490 |
| 2 | 1 | 0 |  | -5.035900 | 2.516229 | 0.383030 |
| 3 | 6 | 0 |  | -4.851810 | 0.591129 | -0.584320 |
| 4 | 6 | 0 |  | -3.990190 | -0.471261 | -0.924240 |
| 5 | 1 | 0 |  | -4.367640 | -1.319901 | -1.482360 |
| 6 | 6 | 0 |  | -2.665330 | -0.402911 | -0.534640 |
| 7 | 6 | 0 |  | -2.197330 | 0.690699 | 0.191920 |
| 8 | 6 | 0 |  | -3.037090 | 1.763699 | 0.529790 |
| 9 | 6 | 0 |  | -1.543580 | -1.382881 | -0.796530 |
| 10 | 1 | 0 |  | -1.301570 | -1.393421 | -1.860990 |
| 11 | 6 | 0 |  | -0.302760 | -0.817191 | -0.007580 |
| 12 | 6 | 0 |  | -0.802360 | 0.547979 | 0.539110 |
| 13 | 6 | 0 |  | 0.120980 | -1.761791 | 1.149730 |
| 14 | 1 | 0 |  | 0.385240 | -2.722251 | 0.689750 |
| 15 | 6 | 0 |  | 1.343020 | -1.240421 | 1.890960 |
| 16 | 1 | 0 |  | 1.051260 | -0.406821 | 2.539820 |
| 17 | 1 | 0 |  | 1.723170 | -2.026241 | 2.550830 |
| 18 | 6 | 0 |  | 2.423320 | -0.786591 | 0.961900 |
| 19 | 1 | 0 |  | 3.420310 | -0.695011 | 1.382710 |
| 20 | 6 | 0 |  | 2.247520 | -0.483001 | -0.336040 |
| 21 | 6 | 0 |  | 0.898060 | -0.545511 | -0.938810 |
| 22 | 8 | 0 |  | -0.134950 | 1.372249 | 1.155590 |
| 23 | 8 | 0 |  | 0.718730 | -0.339771 | -2.129630 |
| 24 | 8 | 0 |  | -1.957550 | -2.690121 | -0.382620 |
| 25 | 1 | 0 |  | -1.485150 | -3.346641 | -0.906040 |
| 26 | 8 | 0 |  | -0.929820 | -1.949591 | 2.085950 |
| 27 | 1 | 0 |  | -1.603450 | -2.461381 | 1.615270 |
| 28 | 6 | 0 |  | 3.363140 | -0.032311 | -1.264170 |
| 29 | 1 | 0 |  | 3.261890 | -0.603051 | -2.194660 |
| 30 | 1 | 0 |  | 3.171740 | 1.006189 | -1.549740 |
| 31 | 6 | 0 |  | 4.754920 | -0.211281 | -0.725540 |
| 32 | 1 | 0 |  | 5.078220 | -1.247631 | -0.637900 |
| 33 | 6 | 0 |  | 5.627180 | 0.738090 | -0.361890 |
| 34 | 6 | 0 |  | 7.007840 | 0.372460 | 0.126800 |
| 35 | 1 | 0 |  | 7.176360 | 0.752350 | 1.141160 |
| 36 | 1 | 0 |  | 7.778510 | 0.826930 | -0.506840 |
| 37 | 1 | 0 |  | 7.164360 | -0.707990 | 0.134150 |
| 38 | 6 | 0 |  | 5.351020 | 2.220179 | -0.398580 |
| 39 | 1 | 0 |  | 5.515750 | 2.660069 | 0.591430 |
| 40 | 1 | 0 |  | 4.335450 | 2.463889 | -0.709060 |
| 41 | 1 | 0 |  | 6.045980 | 2.723540 | -1.080730 |
| 42 | 8 | 0 |  | -2.574600 | 2.823389 | 1.223760 |
| 43 | 1 | 0 |  | -1.627020 | 2.668939 | 1.403270 |
| 44 | 6 | 0 |  | -6.307820 | 0.526589 | -0.971140 |
| 45 | 1 | 0 |  | -6.438200 | 0.043909 | -1.941900 |
| 46 | 1 | 0 |  | -6.756800 | 1.520149 | -1.011630 |
| 47 | 1 | 0 |  | -6.869270 | -0.061641 | -0.237240 |
| **Conformer b-4** | | |  |  |  |  |
| Center | Atomic | Atomic |  | Coordinates (Angstroms) | | |
| Number | Number | Type |  | X | Y | Z |
| 1 | 6 | 0 |  | 3.710630 | -1.870209 | 0.745531 |
| 2 | 1 | 0 |  | 4.287790 | -2.588429 | 1.316201 |
| 3 | 6 | 0 |  | 4.178940 | -1.408949 | -0.492299 |
| 4 | 6 | 0 |  | 3.427739 | -0.480539 | -1.242099 |
| 5 | 1 | 0 |  | 3.794419 | -0.131999 | -2.200489 |
| 6 | 6 | 0 |  | 2.224029 | -0.032869 | -0.731289 |
| 7 | 6 | 0 |  | 1.770659 | -0.481999 | 0.508461 |
| 8 | 6 | 0 |  | 2.499090 | -1.412789 | 1.264911 |
| 9 | 6 | 0 |  | 1.226899 | 0.926421 | -1.342209 |
| 10 | 1 | 0 |  | 0.765719 | 0.473360 | -2.221669 |
| 11 | 6 | 0 |  | 0.115389 | 1.127900 | -0.245099 |
| 12 | 6 | 0 |  | 0.520009 | 0.138380 | 0.880631 |
| 13 | 6 | 0 |  | 0.085389 | 2.590940 | 0.279111 |
| 14 | 1 | 0 |  | -0.134361 | 3.227710 | -0.586959 |
| 15 | 6 | 0 |  | -1.007751 | 2.805940 | 1.314911 |
| 16 | 1 | 0 |  | -0.697961 | 2.367910 | 2.270501 |
| 17 | 1 | 0 |  | -1.127911 | 3.879030 | 1.491341 |
| 18 | 6 | 0 |  | -2.311971 | 2.203540 | 0.901101 |
| 19 | 1 | 0 |  | -3.197231 | 2.570000 | 1.414801 |
| 20 | 6 | 0 |  | -2.463241 | 1.253120 | -0.038319 |
| 21 | 6 | 0 |  | -1.284281 | 0.722580 | -0.758669 |
| 22 | 8 | 0 |  | -0.108261 | -0.089890 | 1.909391 |
| 23 | 8 | 0 |  | -1.397561 | -0.034210 | -1.709699 |
| 24 | 8 | 0 |  | 1.905409 | 2.130421 | -1.721559 |
| 25 | 1 | 0 |  | 1.432349 | 2.535681 | -2.456599 |
| 26 | 8 | 0 |  | 1.328499 | 2.968121 | 0.851311 |
| 27 | 1 | 0 |  | 1.951229 | 3.000321 | 0.110451 |
| 28 | 6 | 0 |  | -3.813591 | 0.660570 | -0.393569 |
| 29 | 1 | 0 |  | -4.588211 | 1.369960 | -0.082209 |
| 30 | 1 | 0 |  | -3.885111 | 0.557790 | -1.476579 |
| 31 | 6 | 0 |  | -4.052561 | -0.662010 | 0.298301 |
| 32 | 1 | 0 |  | -4.035271 | -0.601220 | 1.385671 |
| 33 | 6 | 0 |  | -4.280060 | -1.861710 | -0.250739 |
| 34 | 6 | 0 |  | -4.516650 | -3.072380 | 0.620191 |
| 35 | 1 | 0 |  | -3.770850 | -3.850130 | 0.417981 |
| 36 | 1 | 0 |  | -5.495750 | -3.519150 | 0.410511 |
| 37 | 1 | 0 |  | -4.474380 | -2.824510 | 1.682631 |
| 38 | 6 | 0 |  | -4.331020 | -2.139490 | -1.731399 |
| 39 | 1 | 0 |  | -3.618760 | -2.930720 | -1.991579 |
| 40 | 1 | 0 |  | -4.098340 | -1.268170 | -2.340939 |
| 41 | 1 | 0 |  | -5.323130 | -2.507830 | -2.017809 |
| 42 | 8 | 0 |  | 2.051450 | -1.849369 | 2.459601 |
| 43 | 1 | 0 |  | 1.199160 | -1.406309 | 2.636531 |
| 44 | 6 | 0 |  | 5.505570 | -1.894209 | -1.020149 |
| 45 | 1 | 0 |  | 5.445270 | -2.119199 | -2.087569 |
| 46 | 1 | 0 |  | 5.845030 | -2.787489 | -0.494409 |
| 47 | 1 | 0 |  | 6.270690 | -1.120619 | -0.895959 |
| **Conformer c-1** | | |  |  |  |  |
| Center | Atomic | Atomic |  | Coordinates (Angstroms) | | |
| Number | Number | Type |  | X | Y | Z |
| 1 | 6 | 0 |  | 4.324330 | -1.648530 | -0.118820 |
| 2 | 1 | 0 |  | 4.980860 | -2.508710 | -0.059260 |
| 3 | 6 | 0 |  | 4.640010 | -0.574110 | -0.960580 |
| 4 | 6 | 0 |  | 3.795541 | 0.554650 | -1.031460 |
| 5 | 1 | 0 |  | 4.055101 | 1.391860 | -1.668530 |
| 6 | 6 | 0 |  | 2.644791 | 0.567110 | -0.270050 |
| 7 | 6 | 0 |  | 2.319090 | -0.517780 | 0.550460 |
| 8 | 6 | 0 |  | 3.161950 | -1.634350 | 0.654170 |
| 9 | 6 | 0 |  | 1.623661 | 1.665940 | -0.060720 |
| 10 | 1 | 0 |  | 2.028661 | 2.349840 | 0.693270 |
| 11 | 6 | 0 |  | 0.389871 | 0.938480 | 0.587080 |
| 12 | 6 | 0 |  | 1.036820 | -0.337850 | 1.195210 |
| 13 | 6 | 0 |  | -0.358759 | 1.842490 | 1.581010 |
| 14 | 1 | 0 |  | 0.330251 | 2.136170 | 2.382460 |
| 15 | 6 | 0 |  | -1.581909 | 1.156390 | 2.178470 |
| 16 | 1 | 0 |  | -1.275759 | 0.481350 | 2.983920 |
| 17 | 1 | 0 |  | -2.216459 | 1.920880 | 2.637890 |
| 18 | 6 | 0 |  | -2.376309 | 0.381710 | 1.173760 |
| 19 | 1 | 0 |  | -3.378550 | 0.086070 | 1.471380 |
| 20 | 6 | 0 |  | -1.949170 | 0.043380 | -0.055230 |
| 21 | 6 | 0 |  | -0.595479 | 0.431270 | -0.490920 |
| 22 | 8 | 0 |  | 0.539290 | -1.085590 | 2.029670 |
| 23 | 8 | 0 |  | -0.233609 | 0.314930 | -1.655930 |
| 24 | 8 | 0 |  | 1.328971 | 2.489980 | -1.172190 |
| 25 | 1 | 0 |  | 0.915191 | 1.910680 | -1.832090 |
| 26 | 8 | 0 |  | -0.828369 | 3.011160 | 0.894970 |
| 27 | 1 | 0 |  | -0.170259 | 3.280480 | 0.239540 |
| 28 | 6 | 0 |  | -2.795250 | -0.688980 | -1.085000 |
| 29 | 1 | 0 |  | -3.031090 | 0.008580 | -1.893210 |
| 30 | 1 | 0 |  | -2.162440 | -1.455830 | -1.547550 |
| 31 | 6 | 0 |  | -4.033300 | -1.341000 | -0.535310 |
| 32 | 1 | 0 |  | -3.845260 | -2.189830 | 0.120570 |
| 33 | 6 | 0 |  | -5.311120 | -1.011670 | -0.765260 |
| 34 | 6 | 0 |  | -6.436240 | -1.807250 | -0.148910 |
| 35 | 1 | 0 |  | -7.084700 | -2.230310 | -0.924990 |
| 36 | 1 | 0 |  | -7.072680 | -1.166460 | 0.472310 |
| 37 | 1 | 0 |  | -6.064690 | -2.626100 | 0.470230 |
| 38 | 6 | 0 |  | -5.761990 | 0.137790 | -1.630910 |
| 39 | 1 | 0 |  | -6.374140 | -0.227040 | -2.463690 |
| 40 | 1 | 0 |  | -4.937839 | 0.717830 | -2.045050 |
| 41 | 1 | 0 |  | -6.397719 | 0.818950 | -1.054140 |
| 42 | 8 | 0 |  | 2.859530 | -2.672620 | 1.459510 |
| 43 | 1 | 0 |  | 2.020990 | -2.465970 | 1.913270 |
| 44 | 6 | 0 |  | 5.886920 | -0.623800 | -1.806390 |
| 45 | 1 | 0 |  | 5.628630 | -0.819080 | -2.852490 |
| 46 | 1 | 0 |  | 6.566810 | -1.410300 | -1.476740 |
| 47 | 1 | 0 |  | 6.417831 | 0.330750 | -1.778930 |
| **Conformer c-2** | | |  |  |  |  |
| Center | Atomic | Atomic |  | Coordinates (Angstroms) | | |
| Number | Number | Type |  | X | Y | Z |
| 1 | 6 | 0 |  | -4.382700 | -0.738431 | -0.913450 |
| 2 | 1 | 0 |  | -5.245490 | -0.843461 | -1.560860 |
| 3 | 6 | 0 |  | -4.209750 | -1.596591 | 0.181120 |
| 4 | 6 | 0 |  | -3.092900 | -1.459240 | 1.031730 |
| 5 | 1 | 0 |  | -2.970850 | -2.119770 | 1.882110 |
| 6 | 6 | 0 |  | -2.168430 | -0.469290 | 0.760180 |
| 7 | 6 | 0 |  | -2.334210 | 0.369360 | -0.344000 |
| 8 | 6 | 0 |  | -3.448000 | 0.259550 | -1.191390 |
| 9 | 6 | 0 |  | -0.930180 | -0.068070 | 1.541400 |
| 10 | 1 | 0 |  | -1.237161 | 0.499590 | 2.423160 |
| 11 | 6 | 0 |  | -0.166251 | 0.936721 | 0.590310 |
| 12 | 6 | 0 |  | -1.227781 | 1.283840 | -0.493980 |
| 13 | 6 | 0 |  | 0.324049 | 2.196651 | 1.346720 |
| 14 | 1 | 0 |  | -0.548221 | 2.748870 | 1.702570 |
| 15 | 6 | 0 |  | 1.175359 | 3.104961 | 0.458250 |
| 16 | 1 | 0 |  | 0.532169 | 3.662961 | -0.230200 |
| 17 | 1 | 0 |  | 1.674979 | 3.844151 | 1.090970 |
| 18 | 6 | 0 |  | 2.187289 | 2.349251 | -0.343260 |
| 19 | 1 | 0 |  | 3.025029 | 2.922281 | -0.733140 |
| 20 | 6 | 0 |  | 2.128779 | 1.032761 | -0.621040 |
| 21 | 6 | 0 |  | 0.977610 | 0.229961 | -0.165670 |
| 22 | 8 | 0 |  | -1.134551 | 2.163750 | -1.344470 |
| 23 | 8 | 0 |  | 0.885720 | -0.964749 | -0.424580 |
| 24 | 8 | 0 |  | -0.178920 | -1.149790 | 2.049290 |
| 25 | 1 | 0 |  | 0.156320 | -1.629509 | 1.276850 |
| 26 | 8 | 0 |  | 1.018399 | 1.829891 | 2.539340 |
| 27 | 1 | 0 |  | 1.829419 | 1.366511 | 2.297180 |
| 28 | 6 | 0 |  | 3.211700 | 0.315031 | -1.404560 |
| 29 | 1 | 0 |  | 2.747290 | -0.384509 | -2.099570 |
| 30 | 1 | 0 |  | 3.742789 | 1.062892 | -2.003850 |
| 31 | 6 | 0 |  | 4.204080 | -0.384738 | -0.504360 |
| 32 | 1 | 0 |  | 4.721050 | 0.280862 | 0.185730 |
| 33 | 6 | 0 |  | 4.522100 | -1.684688 | -0.467470 |
| 34 | 6 | 0 |  | 5.569090 | -2.192098 | 0.494910 |
| 35 | 1 | 0 |  | 6.387620 | -2.684818 | -0.042910 |
| 36 | 1 | 0 |  | 5.145170 | -2.943268 | 1.171600 |
| 37 | 1 | 0 |  | 5.992300 | -1.386688 | 1.098360 |
| 38 | 6 | 0 |  | 3.909050 | -2.748968 | -1.341060 |
| 39 | 1 | 0 |  | 4.671341 | -3.200268 | -1.987000 |
| 40 | 1 | 0 |  | 3.102260 | -2.376879 | -1.969920 |
| 41 | 1 | 0 |  | 3.506461 | -3.559078 | -0.722560 |
| 42 | 8 | 0 |  | -3.609621 | 1.079730 | -2.249430 |
| 43 | 1 | 0 |  | -2.854631 | 1.698230 | -2.264700 |
| 44 | 6 | 0 |  | -5.217860 | -2.685741 | 0.447280 |
| 45 | 1 | 0 |  | -4.807279 | -3.661051 | 0.165640 |
| 46 | 1 | 0 |  | -6.136390 | -2.531911 | -0.120170 |
| 47 | 1 | 0 |  | -5.468650 | -2.737181 | 1.509560 |
| **Conformer c-3** | | |  |  |  |  |
| Center | Atomic | Atomic |  | Coordinates (Angstroms) | | |
| Number | Number | Type |  | X | Y | Z |
| 1 | 6 | 0 |  | 4.039392 | -1.917111 | 0.214670 |
| 2 | 1 | 0 |  | 4.587362 | -2.833761 | 0.399300 |
| 3 | 6 | 0 |  | 4.521521 | -0.978361 | -0.707140 |
| 4 | 6 | 0 |  | 3.820681 | 0.223869 | -0.941260 |
| 5 | 1 | 0 |  | 4.209761 | 0.955809 | -1.639080 |
| 6 | 6 | 0 |  | 2.641001 | 0.444159 | -0.259180 |
| 7 | 6 | 0 |  | 2.148601 | -0.506411 | 0.640740 |
| 8 | 6 | 0 |  | 2.848301 | -1.692981 | 0.906950 |
| 9 | 6 | 0 |  | 1.745651 | 1.665569 | -0.222870 |
| 10 | 1 | 0 |  | 2.188001 | 2.375069 | 0.484720 |
| 11 | 6 | 0 |  | 0.403861 | 1.151208 | 0.414720 |
| 12 | 6 | 0 |  | 0.864191 | -0.119792 | 1.181790 |
| 13 | 6 | 0 |  | -0.282439 | 2.231698 | 1.267560 |
| 14 | 1 | 0 |  | 0.395271 | 2.530178 | 2.077010 |
| 15 | 6 | 0 |  | -1.604899 | 1.751448 | 1.854300 |
| 16 | 1 | 0 |  | -1.420129 | 1.137838 | 2.741710 |
| 17 | 1 | 0 |  | -2.169749 | 2.625718 | 2.193510 |
| 18 | 6 | 0 |  | -2.431339 | 0.964198 | 0.885930 |
| 19 | 1 | 0 |  | -3.469349 | 0.796788 | 1.159050 |
| 20 | 6 | 0 |  | -1.989079 | 0.464058 | -0.281010 |
| 21 | 6 | 0 |  | -0.578539 | 0.649378 | -0.668930 |
| 22 | 8 | 0 |  | 0.239271 | -0.718012 | 2.050490 |
| 23 | 8 | 0 |  | -0.171719 | 0.368298 | -1.790120 |
| 24 | 8 | 0 |  | 1.605731 | 2.396879 | -1.425720 |
| 25 | 1 | 0 |  | 1.160131 | 1.801878 | -2.049910 |
| 26 | 8 | 0 |  | -0.579029 | 3.366258 | 0.442430 |
| 27 | 1 | 0 |  | 0.140021 | 3.494418 | -0.191450 |
| 28 | 6 | 0 |  | -2.854339 | -0.294692 | -1.274240 |
| 29 | 1 | 0 |  | -2.687569 | 0.157128 | -2.259260 |
| 30 | 1 | 0 |  | -2.465949 | -1.313422 | -1.362050 |
| 31 | 6 | 0 |  | -4.326569 | -0.287492 | -0.972610 |
| 32 | 1 | 0 |  | -4.811479 | 0.679607 | -1.097300 |
| 33 | 6 | 0 |  | -5.094039 | -1.316123 | -0.588990 |
| 34 | 6 | 0 |  | -6.575239 | -1.132463 | -0.363810 |
| 35 | 1 | 0 |  | -6.850249 | -1.398743 | 0.663290 |
| 36 | 1 | 0 |  | -7.153518 | -1.792233 | -1.021230 |
| 37 | 1 | 0 |  | -6.891239 | -0.103713 | -0.547730 |
| 38 | 6 | 0 |  | -4.597188 | -2.719922 | -0.351590 |
| 39 | 1 | 0 |  | -4.845998 | -3.042223 | 0.665860 |
| 40 | 1 | 0 |  | -3.521068 | -2.827442 | -0.483840 |
| 41 | 1 | 0 |  | -5.095598 | -3.422543 | -1.029410 |
| 42 | 8 | 0 |  | 2.384952 | -2.600691 | 1.789830 |
| 43 | 1 | 0 |  | 1.554152 | -2.255311 | 2.167040 |
| 44 | 6 | 0 |  | 5.793681 | -1.259251 | -1.465760 |
| 45 | 1 | 0 |  | 5.562411 | -1.594811 | -2.482340 |
| 46 | 1 | 0 |  | 6.385402 | -2.038631 | -0.984020 |
| 47 | 1 | 0 |  | 6.406451 | -0.359371 | -1.554760 |
| **Conformer c-4** | | |  |  |  |  |
| Center | Atomic | Atomic |  | Coordinates (Angstroms) | | |
| Number | Number | Type |  | X | Y | Z |
| 1 | 6 | 0 |  | -3.558561 | 1.451769 | 0.791251 |
| 2 | 1 | 0 |  | -4.127331 | 2.233759 | 1.280801 |
| 3 | 6 | 0 |  | -3.937720 | 0.988479 | -0.475649 |
| 4 | 6 | 0 |  | -3.208920 | -0.038771 | -1.112029 |
| 5 | 1 | 0 |  | -3.518000 | -0.407271 | -2.082989 |
| 6 | 6 | 0 |  | -2.105320 | -0.561390 | -0.468899 |
| 7 | 6 | 0 |  | -1.714660 | -0.080460 | 0.785081 |
| 8 | 6 | 0 |  | -2.444510 | 0.920530 | 1.443411 |
| 9 | 6 | 0 |  | -1.209790 | -1.721810 | -0.850629 |
| 10 | 1 | 0 |  | -1.715579 | -2.641910 | -0.538289 |
| 11 | 6 | 0 |  | 0.057250 | -1.556600 | 0.063851 |
| 12 | 6 | 0 |  | -0.489690 | -0.701230 | 1.240191 |
| 13 | 6 | 0 |  | 0.670701 | -2.914359 | 0.448861 |
| 14 | 1 | 0 |  | -0.080679 | -3.502760 | 0.989891 |
| 15 | 6 | 0 |  | 1.922841 | -2.764479 | 1.305741 |
| 16 | 1 | 0 |  | 1.643141 | -2.572519 | 2.346651 |
| 17 | 1 | 0 |  | 2.462471 | -3.716759 | 1.298841 |
| 18 | 6 | 0 |  | 2.826790 | -1.666019 | 0.841121 |
| 19 | 1 | 0 |  | 3.833890 | -1.665508 | 1.251201 |
| 20 | 6 | 0 |  | 2.491620 | -0.700899 | -0.033119 |
| 21 | 6 | 0 |  | 1.136170 | -0.679769 | -0.615519 |
| 22 | 8 | 0 |  | 0.039230 | -0.534800 | 2.333711 |
| 23 | 8 | 0 |  | 0.849330 | 0.025131 | -1.574559 |
| 24 | 8 | 0 |  | -0.945819 | -1.906150 | -2.228329 |
| 25 | 1 | 0 |  | -0.441490 | -1.125680 | -2.509469 |
| 26 | 8 | 0 |  | 1.061931 | -3.608319 | -0.743429 |
| 27 | 1 | 0 |  | 0.410181 | -3.437260 | -1.437489 |
| 28 | 6 | 0 |  | 3.452030 | 0.388151 | -0.471429 |
| 29 | 1 | 0 |  | 4.467670 | 0.062642 | -0.221389 |
| 30 | 1 | 0 |  | 3.404860 | 0.490441 | -1.556109 |
| 31 | 6 | 0 |  | 3.171139 | 1.704731 | 0.215371 |
| 32 | 1 | 0 |  | 3.236629 | 1.659931 | 1.301671 |
| 33 | 6 | 0 |  | 2.863209 | 2.884361 | -0.338159 |
| 34 | 6 | 0 |  | 2.629809 | 4.100371 | 0.526031 |
| 35 | 1 | 0 |  | 1.622208 | 4.502921 | 0.368881 |
| 36 | 1 | 0 |  | 3.329268 | 4.903791 | 0.266681 |
| 37 | 1 | 0 |  | 2.746589 | 3.874201 | 1.587701 |
| 38 | 6 | 0 |  | 2.720229 | 3.134961 | -1.817569 |
| 39 | 1 | 0 |  | 1.726029 | 3.540181 | -2.037689 |
| 40 | 1 | 0 |  | 2.857589 | 2.240421 | -2.422589 |
| 41 | 1 | 0 |  | 3.443689 | 3.889021 | -2.148839 |
| 42 | 8 | 0 |  | -2.079801 | 1.373350 | 2.660081 |
| 43 | 1 | 0 |  | -1.289750 | 0.875050 | 2.941261 |
| 44 | 6 | 0 |  | -5.126691 | 1.599379 | -1.172539 |
| 45 | 1 | 0 |  | -4.794471 | 2.301559 | -1.944619 |
| 46 | 1 | 0 |  | -5.763991 | 2.146018 | -0.476399 |
| 47 | 1 | 0 |  | -5.727450 | 0.834358 | -1.669649 |
| **Conformer d-1** | | |  |  |  |  |
| Center | Atomic | Atomic |  | Coordinates (Angstroms) | | |
| Number | Number | Type |  | X | Y | Z |
| 1 | 6 | 0 |  | -4.187441 | -1.035409 | -0.964310 |
| 2 | 1 | 0 |  | -5.017431 | -1.227919 | -1.634110 |
| 3 | 6 | 0 |  | -3.992691 | -1.835719 | 0.170110 |
| 4 | 6 | 0 |  | -2.922201 | -1.582310 | 1.053030 |
| 5 | 1 | 0 |  | -2.788031 | -2.194310 | 1.937150 |
| 6 | 6 | 0 |  | -2.060730 | -0.540260 | 0.769700 |
| 7 | 6 | 0 |  | -2.243680 | 0.238520 | -0.376740 |
| 8 | 6 | 0 |  | -3.318420 | 0.017320 | -1.251910 |
| 9 | 6 | 0 |  | -0.897340 | 0.001180 | 1.577580 |
| 10 | 1 | 0 |  | -1.297890 | 0.636890 | 2.371460 |
| 11 | 6 | 0 |  | -0.139200 | 0.960799 | 0.581890 |
| 12 | 6 | 0 |  | -1.195720 | 1.219460 | -0.525200 |
| 13 | 6 | 0 |  | 0.342470 | 2.255419 | 1.275100 |
| 14 | 1 | 0 |  | 0.943790 | 1.943529 | 2.134400 |
| 15 | 6 | 0 |  | 1.205621 | 3.127799 | 0.365050 |
| 16 | 1 | 0 |  | 0.567431 | 3.659029 | -0.351170 |
| 17 | 1 | 0 |  | 1.704661 | 3.895059 | 0.964200 |
| 18 | 6 | 0 |  | 2.219021 | 2.336789 | -0.397760 |
| 19 | 1 | 0 |  | 3.052841 | 2.895878 | -0.814670 |
| 20 | 6 | 0 |  | 2.171360 | 1.008069 | -0.604720 |
| 21 | 6 | 0 |  | 1.029960 | 0.218959 | -0.103920 |
| 22 | 8 | 0 |  | -1.142720 | 2.095120 | -1.385840 |
| 23 | 8 | 0 |  | 0.967739 | -0.994121 | -0.260930 |
| 24 | 8 | 0 |  | -0.109211 | -0.967421 | 2.233930 |
| 25 | 1 | 0 |  | 0.277229 | -1.517681 | 1.536720 |
| 26 | 8 | 0 |  | -0.742459 | 2.987300 | 1.838530 |
| 27 | 1 | 0 |  | -1.219119 | 3.425090 | 1.122710 |
| 28 | 6 | 0 |  | 3.263180 | 0.258438 | -1.345410 |
| 29 | 1 | 0 |  | 2.807990 | -0.492542 | -1.991220 |
| 30 | 1 | 0 |  | 3.779560 | 0.974448 | -1.994370 |
| 31 | 6 | 0 |  | 4.270080 | -0.364162 | -0.405750 |
| 32 | 1 | 0 |  | 4.776180 | 0.354168 | 0.237920 |
| 33 | 6 | 0 |  | 4.611479 | -1.652822 | -0.284010 |
| 34 | 6 | 0 |  | 5.668689 | -2.076073 | 0.707560 |
| 35 | 1 | 0 |  | 6.495639 | -2.588373 | 0.201830 |
| 36 | 1 | 0 |  | 5.259609 | -2.788503 | 1.433650 |
| 37 | 1 | 0 |  | 6.077429 | -1.224913 | 1.255620 |
| 38 | 6 | 0 |  | 4.017739 | -2.784112 | -1.083420 |
| 39 | 1 | 0 |  | 4.790729 | -3.271622 | -1.689030 |
| 40 | 1 | 0 |  | 3.212719 | -2.468462 | -1.744770 |
| 41 | 1 | 0 |  | 3.618539 | -3.552672 | -0.411900 |
| 42 | 8 | 0 |  | -3.503460 | 0.784740 | -2.345560 |
| 43 | 1 | 0 |  | -2.801230 | 1.461940 | -2.357680 |
| 44 | 6 | 0 |  | -4.928051 | -2.986109 | 0.443900 |
| 45 | 1 | 0 |  | -4.455142 | -3.934099 | 0.166110 |
| 46 | 1 | 0 |  | -5.854511 | -2.896149 | -0.124750 |
| 47 | 1 | 0 |  | -5.174851 | -3.048379 | 1.506260 |
| **Conformer d-2** | | |  |  |  |  |
| Center | Atomic | Atomic |  | Coordinates (Angstroms) | | |
| Number | Number | Type |  | X | Y | Z |
| 1 | 6 | 0 |  | 4.444970 | -0.465880 | 1.395170 |
| 2 | 1 | 0 |  | 5.175250 | -0.519330 | 2.194230 |
| 3 | 6 | 0 |  | 4.664310 | -1.145400 | 0.188470 |
| 4 | 6 | 0 |  | 3.724550 | -1.068030 | -0.860320 |
| 5 | 1 | 0 |  | 3.909290 | -1.580660 | -1.796990 |
| 6 | 6 | 0 |  | 2.576760 | -0.323070 | -0.669750 |
| 7 | 6 | 0 |  | 2.350010 | 0.330960 | 0.544810 |
| 8 | 6 | 0 |  | 3.286350 | 0.286140 | 1.589670 |
| 9 | 6 | 0 |  | 1.460670 | 0.002310 | -1.643370 |
| 10 | 1 | 0 |  | 1.799150 | 0.814910 | -2.291540 |
| 11 | 6 | 0 |  | 0.316510 | 0.605690 | -0.742310 |
| 12 | 6 | 0 |  | 1.066000 | 0.989810 | 0.561470 |
| 13 | 6 | 0 |  | -0.382590 | 1.806950 | -1.418870 |
| 14 | 1 | 0 |  | -0.722770 | 1.457680 | -2.398470 |
| 15 | 6 | 0 |  | -1.591340 | 2.310210 | -0.631710 |
| 16 | 1 | 0 |  | -1.249810 | 2.898810 | 0.228420 |
| 17 | 1 | 0 |  | -2.175640 | 2.989100 | -1.259510 |
| 18 | 6 | 0 |  | -2.460550 | 1.201440 | -0.130440 |
| 19 | 1 | 0 |  | -3.477410 | 1.466320 | 0.143520 |
| 20 | 6 | 0 |  | -2.076370 | -0.080010 | 0.006370 |
| 21 | 6 | 0 |  | -0.702160 | -0.484360 | -0.346100 |
| 22 | 8 | 0 |  | 0.636430 | 1.714700 | 1.455750 |
| 23 | 8 | 0 |  | -0.352530 | -1.658030 | -0.299210 |
| 24 | 8 | 0 |  | 1.086060 | -1.042120 | -2.514070 |
| 25 | 1 | 0 |  | 0.748720 | -1.754120 | -1.950380 |
| 26 | 8 | 0 |  | 0.536090 | 2.857230 | -1.707300 |
| 27 | 1 | 0 |  | 0.742110 | 3.315530 | -0.883350 |
| 28 | 6 | 0 |  | -2.986970 | -1.193340 | 0.498860 |
| 29 | 1 | 0 |  | -3.139100 | -1.898490 | -0.322700 |
| 30 | 1 | 0 |  | -2.432520 | -1.759250 | 1.256860 |
| 31 | 6 | 0 |  | -4.292450 | -0.733610 | 1.085830 |
| 32 | 1 | 0 |  | -4.205410 | -0.224800 | 2.044760 |
| 33 | 6 | 0 |  | -5.522920 | -0.885620 | 0.578780 |
| 34 | 6 | 0 |  | -6.733650 | -0.384760 | 1.328170 |
| 35 | 1 | 0 |  | -7.426130 | -1.206850 | 1.543920 |
| 36 | 1 | 0 |  | -7.292060 | 0.341930 | 0.726780 |
| 37 | 1 | 0 |  | -6.460450 | 0.089050 | 2.272970 |
| 38 | 6 | 0 |  | -5.835250 | -1.546790 | -0.739910 |
| 39 | 1 | 0 |  | -6.491070 | -2.411950 | -0.588950 |
| 40 | 1 | 0 |  | -4.949020 | -1.882820 | -1.277310 |
| 41 | 1 | 0 |  | -6.381370 | -0.854070 | -1.390170 |
| 42 | 8 | 0 |  | 3.073720 | 0.935780 | 2.752510 |
| 43 | 1 | 0 |  | 2.221730 | 1.405650 | 2.681350 |
| 44 | 6 | 0 |  | 5.909260 | -1.978080 | 0.015180 |
| 45 | 1 | 0 |  | 5.682210 | -3.037350 | 0.176830 |
| 46 | 1 | 0 |  | 6.686510 | -1.692710 | 0.725370 |
| 47 | 1 | 0 |  | 6.308760 | -1.884420 | -0.997030 |
| **Conformer d-3** | | |  |  |  |  |
| Center | Atomic | Atomic |  | Coordinates (Angstroms) | | |
| Number | Number | Type |  | X | Y | Z |
| 1 | 6 | 0 |  | 4.222350 | -1.630201 | 0.586600 |
| 2 | 1 | 0 |  | 4.853260 | -2.392211 | 1.029350 |
| 3 | 6 | 0 |  | 4.603290 | -1.001651 | -0.607710 |
| 4 | 6 | 0 |  | 3.793701 | -0.000051 | -1.181670 |
| 5 | 1 | 0 |  | 4.102741 | 0.494309 | -2.094980 |
| 6 | 6 | 0 |  | 2.610501 | 0.338149 | -0.553160 |
| 7 | 6 | 0 |  | 2.222280 | -0.309311 | 0.623210 |
| 8 | 6 | 0 |  | 3.028620 | -1.290091 | 1.222300 |
| 9 | 6 | 0 |  | 1.607721 | 1.421890 | -0.897170 |
| 10 | 1 | 0 |  | 1.994981 | 2.375339 | -0.528480 |
| 11 | 6 | 0 |  | 0.347191 | 1.086490 | -0.012540 |
| 12 | 6 | 0 |  | 0.925661 | 0.138180 | 1.072580 |
| 13 | 6 | 0 |  | -0.306609 | 2.358340 | 0.572720 |
| 14 | 1 | 0 |  | -0.517739 | 3.017470 | -0.274850 |
| 15 | 6 | 0 |  | -1.615129 | 2.069510 | 1.305830 |
| 16 | 1 | 0 |  | -1.398049 | 1.640460 | 2.291550 |
| 17 | 1 | 0 |  | -2.143859 | 3.009801 | 1.487580 |
| 18 | 6 | 0 |  | -2.499559 | 1.121001 | 0.562120 |
| 19 | 1 | 0 |  | -3.547219 | 1.106851 | 0.847930 |
| 20 | 6 | 0 |  | -2.094159 | 0.289101 | -0.413610 |
| 21 | 6 | 0 |  | -0.673899 | 0.248260 | -0.811680 |
| 22 | 8 | 0 |  | 0.370721 | -0.186800 | 2.119520 |
| 23 | 8 | 0 |  | -0.289760 | -0.461110 | -1.734180 |
| 24 | 8 | 0 |  | 1.371271 | 1.619180 | -2.273770 |
| 25 | 1 | 0 |  | 0.998181 | 0.788720 | -2.604560 |
| 26 | 8 | 0 |  | 0.604861 | 3.093780 | 1.384540 |
| 27 | 1 | 0 |  | 0.699861 | 2.634350 | 2.227900 |
| 28 | 6 | 0 |  | -3.017040 | -0.666289 | -1.153040 |
| 29 | 1 | 0 |  | -2.774070 | -0.592909 | -2.219100 |
| 30 | 1 | 0 |  | -2.748850 | -1.688879 | -0.871940 |
| 31 | 6 | 0 |  | -4.485340 | -0.405499 | -0.962750 |
| 32 | 1 | 0 |  | -4.849799 | 0.502941 | -1.440230 |
| 33 | 6 | 0 |  | -5.376350 | -1.152329 | -0.297510 |
| 34 | 6 | 0 |  | -6.830510 | -0.751458 | -0.240770 |
| 35 | 1 | 0 |  | -7.156880 | -0.611158 | 0.796240 |
| 36 | 1 | 0 |  | -7.467570 | -1.537238 | -0.663330 |
| 37 | 1 | 0 |  | -7.019589 | 0.173762 | -0.788480 |
| 38 | 6 | 0 |  | -5.051100 | -2.427699 | 0.438140 |
| 39 | 1 | 0 |  | -5.364090 | -2.350289 | 1.485430 |
| 40 | 1 | 0 |  | -3.990670 | -2.677589 | 0.422690 |
| 41 | 1 | 0 |  | -5.606430 | -3.270299 | 0.010220 |
| 42 | 8 | 0 |  | 2.660690 | -1.898511 | 2.368630 |
| 43 | 1 | 0 |  | 1.809750 | -1.514630 | 2.651780 |
| 44 | 6 | 0 |  | 5.881900 | -1.413532 | -1.292460 |
| 45 | 1 | 0 |  | 5.672030 | -2.153781 | -2.072170 |
| 46 | 1 | 0 |  | 6.586720 | -1.862912 | -0.591250 |
| 47 | 1 | 0 |  | 6.363900 | -0.561282 | -1.775840 |
| **Conformer d-4** | | |  |  |  |  |
| Center | Atomic | Atomic |  | Coordinates (Angstroms) | | |
| Number | Number | Type |  | X | Y | Z |
| 1 | 6 | 0 |  | 3.479820 | -1.708061 | 0.943700 |
| 2 | 1 | 0 |  | 4.001010 | -2.425172 | 1.567230 |
| 3 | 6 | 0 |  | 3.854960 | -1.529142 | -0.395540 |
| 4 | 6 | 0 |  | 3.190191 | -0.587321 | -1.207860 |
| 5 | 1 | 0 |  | 3.498011 | -0.440641 | -2.236420 |
| 6 | 6 | 0 |  | 2.151551 | 0.143289 | -0.663640 |
| 7 | 6 | 0 |  | 1.763211 | -0.058271 | 0.664090 |
| 8 | 6 | 0 |  | 2.431261 | -0.971771 | 1.494740 |
| 9 | 6 | 0 |  | 1.336151 | 1.268779 | -1.270350 |
| 10 | 1 | 0 |  | 1.923121 | 2.188479 | -1.202170 |
| 11 | 6 | 0 |  | 0.120431 | 1.447539 | -0.282930 |
| 12 | 6 | 0 |  | 0.616041 | 0.746179 | 1.009900 |
| 13 | 6 | 0 |  | -0.241159 | 2.935979 | -0.071100 |
| 14 | 1 | 0 |  | -0.412628 | 3.357329 | -1.066030 |
| 15 | 6 | 0 |  | -1.503438 | 3.129830 | 0.768110 |
| 16 | 1 | 0 |  | -1.266319 | 2.980270 | 1.828490 |
| 17 | 1 | 0 |  | -1.848268 | 4.163410 | 0.671420 |
| 18 | 6 | 0 |  | -2.601439 | 2.185140 | 0.397970 |
| 19 | 1 | 0 |  | -3.600679 | 2.456080 | 0.729130 |
| 20 | 6 | 0 |  | -2.443279 | 1.040670 | -0.290730 |
| 21 | 6 | 0 |  | -1.104129 | 0.636810 | -0.761990 |
| 22 | 8 | 0 |  | 0.110881 | 0.844369 | 2.125670 |
| 23 | 8 | 0 |  | -0.947129 | -0.332330 | -1.493980 |
| 24 | 8 | 0 |  | 1.017501 | 1.126839 | -2.636790 |
| 25 | 1 | 0 |  | 0.454371 | 0.340679 | -2.699050 |
| 26 | 8 | 0 |  | 0.858872 | 3.676029 | 0.450580 |
| 27 | 1 | 0 |  | 0.956982 | 3.456919 | 1.385370 |
| 28 | 6 | 0 |  | -3.597429 | 0.108040 | -0.605300 |
| 29 | 1 | 0 |  | -4.527379 | 0.679510 | -0.511040 |
| 30 | 1 | 0 |  | -3.521789 | -0.216640 | -1.643320 |
| 31 | 6 | 0 |  | -3.646569 | -1.074070 | 0.335070 |
| 32 | 1 | 0 |  | -3.722679 | -0.799940 | 1.386430 |
| 33 | 6 | 0 |  | -3.617410 | -2.378500 | 0.035100 |
| 34 | 6 | 0 |  | -3.696680 | -3.419100 | 1.126330 |
| 35 | 1 | 0 |  | -2.814840 | -4.070290 | 1.109090 |
| 36 | 1 | 0 |  | -4.566570 | -4.070580 | 0.982010 |
| 37 | 1 | 0 |  | -3.768980 | -2.966070 | 2.117020 |
| 38 | 6 | 0 |  | -3.509260 | -2.944060 | -1.357770 |
| 39 | 1 | 0 |  | -2.620710 | -3.580390 | -1.439760 |
| 40 | 1 | 0 |  | -3.446100 | -2.180310 | -2.131080 |
| 41 | 1 | 0 |  | -4.371640 | -3.584050 | -1.577380 |
| 42 | 8 | 0 |  | 2.067330 | -1.147211 | 2.781730 |
| 43 | 1 | 0 |  | 1.330391 | -0.536261 | 2.970060 |
| 44 | 6 | 0 |  | 4.969280 | -2.362192 | -0.976410 |
| 45 | 1 | 0 |  | 4.557160 | -3.209282 | -1.535420 |
| 46 | 1 | 0 |  | 5.619210 | -2.763562 | -0.197610 |
| 47 | 1 | 0 |  | 5.576060 | -1.779672 | -1.672990 |
| **Conformer e-1** | | |  |  |  |  |
| Center | Atomic | Atomic |  | Coordinates (Angstroms) | | |
| Number | Number | Type |  | X | Y | Z |
| 1 | 6 | 0 |  | 4.187441 | -1.035409 | -0.964310 |
| 2 | 1 | 0 |  | 5.017431 | -1.227919 | -1.634110 |
| 3 | 6 | 0 |  | 3.992691 | -1.835719 | 0.170110 |
| 4 | 6 | 0 |  | 2.922201 | -1.582310 | 1.053030 |
| 5 | 1 | 0 |  | 2.788031 | -2.194310 | 1.937150 |
| 6 | 6 | 0 |  | 2.060730 | -0.540260 | 0.769700 |
| 7 | 6 | 0 |  | 2.243680 | 0.238520 | -0.376740 |
| 8 | 6 | 0 |  | 3.318420 | 0.017320 | -1.251910 |
| 9 | 6 | 0 |  | 0.897340 | 0.001180 | 1.577580 |
| 10 | 1 | 0 |  | 1.297890 | 0.636890 | 2.371460 |
| 11 | 6 | 0 |  | 0.139200 | 0.960799 | 0.581890 |
| 12 | 6 | 0 |  | 1.195720 | 1.219460 | -0.525200 |
| 13 | 6 | 0 |  | -0.342470 | 2.255419 | 1.275100 |
| 14 | 1 | 0 |  | -0.943790 | 1.943529 | 2.134400 |
| 15 | 6 | 0 |  | -1.205621 | 3.127799 | 0.365050 |
| 16 | 1 | 0 |  | -0.567431 | 3.659029 | -0.351170 |
| 17 | 1 | 0 |  | -1.704661 | 3.895059 | 0.964200 |
| 18 | 6 | 0 |  | -2.219021 | 2.336789 | -0.397760 |
| 19 | 1 | 0 |  | -3.052841 | 2.895878 | -0.814670 |
| 20 | 6 | 0 |  | -2.171360 | 1.008069 | -0.604720 |
| 21 | 6 | 0 |  | -1.029960 | 0.218959 | -0.103920 |
| 22 | 8 | 0 |  | 1.142720 | 2.095120 | -1.385840 |
| 23 | 8 | 0 |  | -0.967739 | -0.994121 | -0.260930 |
| 24 | 8 | 0 |  | 0.109211 | -0.967421 | 2.233930 |
| 25 | 1 | 0 |  | -0.277229 | -1.517681 | 1.536720 |
| 26 | 8 | 0 |  | 0.742459 | 2.987300 | 1.838530 |
| 27 | 1 | 0 |  | 1.219119 | 3.425090 | 1.122710 |
| 28 | 6 | 0 |  | -3.263180 | 0.258438 | -1.345410 |
| 29 | 1 | 0 |  | -2.807990 | -0.492542 | -1.991220 |
| 30 | 1 | 0 |  | -3.779560 | 0.974448 | -1.994370 |
| 31 | 6 | 0 |  | -4.270080 | -0.364162 | -0.405750 |
| 32 | 1 | 0 |  | -4.776180 | 0.354168 | 0.237920 |
| 33 | 6 | 0 |  | -4.611479 | -1.652822 | -0.284010 |
| 34 | 6 | 0 |  | -5.668689 | -2.076073 | 0.707560 |
| 35 | 1 | 0 |  | -6.495639 | -2.588373 | 0.201830 |
| 36 | 1 | 0 |  | -5.259609 | -2.788503 | 1.433650 |
| 37 | 1 | 0 |  | -6.077429 | -1.224913 | 1.255620 |
| 38 | 6 | 0 |  | -4.017739 | -2.784112 | -1.083420 |
| 39 | 1 | 0 |  | -4.790729 | -3.271622 | -1.689030 |
| 40 | 1 | 0 |  | -3.212719 | -2.468462 | -1.744770 |
| 41 | 1 | 0 |  | -3.618539 | -3.552672 | -0.411900 |
| 42 | 8 | 0 |  | 3.503460 | 0.784740 | -2.345560 |
| 43 | 1 | 0 |  | 2.801230 | 1.461940 | -2.357680 |
| 44 | 6 | 0 |  | 4.928051 | -2.986109 | 0.443900 |
| 45 | 1 | 0 |  | 4.455142 | -3.934099 | 0.166110 |
| 46 | 1 | 0 |  | 5.854511 | -2.896149 | -0.124750 |
| 47 | 1 | 0 |  | 5.174851 | -3.048379 | 1.506260 |
| **Conformer e-2** | | |  |  |  |  |
| Center | Atomic | Atomic |  | Coordinates (Angstroms) | | |
| Number | Number | Type |  | X | Y | Z |
| 1 | 6 | 0 |  | -4.444970 | -0.465880 | 1.395170 |
| 2 | 1 | 0 |  | -5.175250 | -0.519330 | 2.194230 |
| 3 | 6 | 0 |  | -4.664310 | -1.145400 | 0.188470 |
| 4 | 6 | 0 |  | -3.724550 | -1.068030 | -0.860320 |
| 5 | 1 | 0 |  | -3.909290 | -1.580660 | -1.796990 |
| 6 | 6 | 0 |  | -2.576760 | -0.323070 | -0.669750 |
| 7 | 6 | 0 |  | -2.350010 | 0.330960 | 0.544810 |
| 8 | 6 | 0 |  | -3.286350 | 0.286140 | 1.589670 |
| 9 | 6 | 0 |  | -1.460670 | 0.002310 | -1.643370 |
| 10 | 1 | 0 |  | -1.799150 | 0.814910 | -2.291540 |
| 11 | 6 | 0 |  | -0.316510 | 0.605690 | -0.742310 |
| 12 | 6 | 0 |  | -1.066000 | 0.989810 | 0.561470 |
| 13 | 6 | 0 |  | 0.382590 | 1.806950 | -1.418870 |
| 14 | 1 | 0 |  | 0.722770 | 1.457680 | -2.398470 |
| 15 | 6 | 0 |  | 1.591340 | 2.310210 | -0.631710 |
| 16 | 1 | 0 |  | 1.249810 | 2.898810 | 0.228420 |
| 17 | 1 | 0 |  | 2.175640 | 2.989100 | -1.259510 |
| 18 | 6 | 0 |  | 2.460550 | 1.201440 | -0.130440 |
| 19 | 1 | 0 |  | 3.477410 | 1.466320 | 0.143520 |
| 20 | 6 | 0 |  | 2.076370 | -0.080010 | 0.006370 |
| 21 | 6 | 0 |  | 0.702160 | -0.484360 | -0.346100 |
| 22 | 8 | 0 |  | -0.636430 | 1.714700 | 1.455750 |
| 23 | 8 | 0 |  | 0.352530 | -1.658030 | -0.299210 |
| 24 | 8 | 0 |  | -1.086060 | -1.042120 | -2.514070 |
| 25 | 1 | 0 |  | -0.748720 | -1.754120 | -1.950380 |
| 26 | 8 | 0 |  | -0.536090 | 2.857230 | -1.707300 |
| 27 | 1 | 0 |  | -0.742110 | 3.315530 | -0.883350 |
| 28 | 6 | 0 |  | 2.986970 | -1.193340 | 0.498860 |
| 29 | 1 | 0 |  | 3.139100 | -1.898490 | -0.322700 |
| 30 | 1 | 0 |  | 2.432520 | -1.759250 | 1.256860 |
| 31 | 6 | 0 |  | 4.292450 | -0.733610 | 1.085830 |
| 32 | 1 | 0 |  | 4.205410 | -0.224800 | 2.044760 |
| 33 | 6 | 0 |  | 5.522920 | -0.885620 | 0.578780 |
| 34 | 6 | 0 |  | 6.733650 | -0.384760 | 1.328170 |
| 35 | 1 | 0 |  | 7.426130 | -1.206850 | 1.543920 |
| 36 | 1 | 0 |  | 7.292060 | 0.341930 | 0.726780 |
| 37 | 1 | 0 |  | 6.460450 | 0.089050 | 2.272970 |
| 38 | 6 | 0 |  | 5.835250 | -1.546790 | -0.739910 |
| 39 | 1 | 0 |  | 6.491070 | -2.411950 | -0.588950 |
| 40 | 1 | 0 |  | 4.949020 | -1.882820 | -1.277310 |
| 41 | 1 | 0 |  | 6.381370 | -0.854070 | -1.390170 |
| 42 | 8 | 0 |  | -3.073720 | 0.935780 | 2.752510 |
| 43 | 1 | 0 |  | -2.221730 | 1.405650 | 2.681350 |
| 44 | 6 | 0 |  | -5.909260 | -1.978080 | 0.015180 |
| 45 | 1 | 0 |  | -5.682210 | -3.037350 | 0.176830 |
| 46 | 1 | 0 |  | -6.686510 | -1.692710 | 0.725370 |
| 47 | 1 | 0 |  | -6.308760 | -1.884420 | -0.997030 |
| **Conformer e-3** | | |  |  |  |  |
| Center | Atomic | Atomic |  | Coordinates (Angstroms) | | |
| Number | Number | Type |  | X | Y | Z |
| 1 | 6 | 0 |  | -4.222350 | -1.630201 | 0.586600 |
| 2 | 1 | 0 |  | -4.853260 | -2.392211 | 1.029350 |
| 3 | 6 | 0 |  | -4.603290 | -1.001651 | -0.607710 |
| 4 | 6 | 0 |  | -3.793701 | -0.000051 | -1.181670 |
| 5 | 1 | 0 |  | -4.102741 | 0.494309 | -2.094980 |
| 6 | 6 | 0 |  | -2.610501 | 0.338149 | -0.553160 |
| 7 | 6 | 0 |  | -2.222280 | -0.309311 | 0.623210 |
| 8 | 6 | 0 |  | -3.028620 | -1.290091 | 1.222300 |
| 9 | 6 | 0 |  | -1.607721 | 1.421890 | -0.897170 |
| 10 | 1 | 0 |  | -1.994981 | 2.375339 | -0.528480 |
| 11 | 6 | 0 |  | -0.347191 | 1.086490 | -0.012540 |
| 12 | 6 | 0 |  | -0.925661 | 0.138180 | 1.072580 |
| 13 | 6 | 0 |  | 0.306609 | 2.358340 | 0.572720 |
| 14 | 1 | 0 |  | 0.517739 | 3.017470 | -0.274850 |
| 15 | 6 | 0 |  | 1.615129 | 2.069510 | 1.305830 |
| 16 | 1 | 0 |  | 1.398049 | 1.640460 | 2.291550 |
| 17 | 1 | 0 |  | 2.143859 | 3.009801 | 1.487580 |
| 18 | 6 | 0 |  | 2.499559 | 1.121001 | 0.562120 |
| 19 | 1 | 0 |  | 3.547219 | 1.106851 | 0.847930 |
| 20 | 6 | 0 |  | 2.094159 | 0.289101 | -0.413610 |
| 21 | 6 | 0 |  | 0.673899 | 0.248260 | -0.811680 |
| 22 | 8 | 0 |  | -0.370721 | -0.186800 | 2.119520 |
| 23 | 8 | 0 |  | 0.289760 | -0.461110 | -1.734180 |
| 24 | 8 | 0 |  | -1.371271 | 1.619180 | -2.273770 |
| 25 | 1 | 0 |  | -0.998181 | 0.788720 | -2.604560 |
| 26 | 8 | 0 |  | -0.604861 | 3.093780 | 1.384540 |
| 27 | 1 | 0 |  | -0.699861 | 2.634350 | 2.227900 |
| 28 | 6 | 0 |  | 3.017040 | -0.666289 | -1.153040 |
| 29 | 1 | 0 |  | 2.774070 | -0.592909 | -2.219100 |
| 30 | 1 | 0 |  | 2.748850 | -1.688879 | -0.871940 |
| 31 | 6 | 0 |  | 4.485340 | -0.405499 | -0.962750 |
| 32 | 1 | 0 |  | 4.849799 | 0.502941 | -1.440230 |
| 33 | 6 | 0 |  | 5.376350 | -1.152329 | -0.297510 |
| 34 | 6 | 0 |  | 6.830510 | -0.751458 | -0.240770 |
| 35 | 1 | 0 |  | 7.156880 | -0.611158 | 0.796240 |
| 36 | 1 | 0 |  | 7.467570 | -1.537238 | -0.663330 |
| 37 | 1 | 0 |  | 7.019589 | 0.173762 | -0.788480 |
| 38 | 6 | 0 |  | 5.051100 | -2.427699 | 0.438140 |
| 39 | 1 | 0 |  | 5.364090 | -2.350289 | 1.485430 |
| 40 | 1 | 0 |  | 3.990670 | -2.677589 | 0.422690 |
| 41 | 1 | 0 |  | 5.606430 | -3.270299 | 0.010220 |
| 42 | 8 | 0 |  | -2.660690 | -1.898511 | 2.368630 |
| 43 | 1 | 0 |  | -1.809750 | -1.514630 | 2.651780 |
| 44 | 6 | 0 |  | -5.881900 | -1.413532 | -1.292460 |
| 45 | 1 | 0 |  | -5.672030 | -2.153781 | -2.072170 |
| 46 | 1 | 0 |  | -6.586720 | -1.862912 | -0.591250 |
| 47 | 1 | 0 |  | -6.363900 | -0.561282 | -1.775840 |
| **Conformer e-4** | | |  |  |  |  |
| Center | Atomic | Atomic |  | Coordinates (Angstroms) | | |
| Number | Number | Type |  | X | Y | Z |
| 1 | 6 | 0 |  | -3.479820 | -1.708061 | 0.943700 |
| 2 | 1 | 0 |  | -4.001010 | -2.425172 | 1.567230 |
| 3 | 6 | 0 |  | -3.854960 | -1.529142 | -0.395540 |
| 4 | 6 | 0 |  | -3.190191 | -0.587321 | -1.207860 |
| 5 | 1 | 0 |  | -3.498011 | -0.440641 | -2.236420 |
| 6 | 6 | 0 |  | -2.151551 | 0.143289 | -0.663640 |
| 7 | 6 | 0 |  | -1.763211 | -0.058271 | 0.664090 |
| 8 | 6 | 0 |  | -2.431261 | -0.971771 | 1.494740 |
| 9 | 6 | 0 |  | -1.336151 | 1.268779 | -1.270350 |
| 10 | 1 | 0 |  | -1.923121 | 2.188479 | -1.202170 |
| 11 | 6 | 0 |  | -0.120431 | 1.447539 | -0.282930 |
| 12 | 6 | 0 |  | -0.616041 | 0.746179 | 1.009900 |
| 13 | 6 | 0 |  | 0.241159 | 2.935979 | -0.071100 |
| 14 | 1 | 0 |  | 0.412628 | 3.357329 | -1.066030 |
| 15 | 6 | 0 |  | 1.503438 | 3.129830 | 0.768110 |
| 16 | 1 | 0 |  | 1.266319 | 2.980270 | 1.828490 |
| 17 | 1 | 0 |  | 1.848268 | 4.163410 | 0.671420 |
| 18 | 6 | 0 |  | 2.601439 | 2.185140 | 0.397970 |
| 19 | 1 | 0 |  | 3.600679 | 2.456080 | 0.729130 |
| 20 | 6 | 0 |  | 2.443279 | 1.040670 | -0.290730 |
| 21 | 6 | 0 |  | 1.104129 | 0.636810 | -0.761990 |
| 22 | 8 | 0 |  | -0.110881 | 0.844369 | 2.125670 |
| 23 | 8 | 0 |  | 0.947129 | -0.332330 | -1.493980 |
| 24 | 8 | 0 |  | -1.017501 | 1.126839 | -2.636790 |
| 25 | 1 | 0 |  | -0.454371 | 0.340679 | -2.699050 |
| 26 | 8 | 0 |  | -0.858872 | 3.676029 | 0.450580 |
| 27 | 1 | 0 |  | -0.956982 | 3.456919 | 1.385370 |
| 28 | 6 | 0 |  | 3.597429 | 0.108040 | -0.605300 |
| 29 | 1 | 0 |  | 4.527379 | 0.679510 | -0.511040 |
| 30 | 1 | 0 |  | 3.521789 | -0.216640 | -1.643320 |
| 31 | 6 | 0 |  | 3.646569 | -1.074070 | 0.335070 |
| 32 | 1 | 0 |  | 3.722679 | -0.799940 | 1.386430 |
| 33 | 6 | 0 |  | 3.617410 | -2.378500 | 0.035100 |
| 34 | 6 | 0 |  | 3.696680 | -3.419100 | 1.126330 |
| 35 | 1 | 0 |  | 2.814840 | -4.070290 | 1.109090 |
| 36 | 1 | 0 |  | 4.566570 | -4.070580 | 0.982010 |
| 37 | 1 | 0 |  | 3.768980 | -2.966070 | 2.117020 |
| 38 | 6 | 0 |  | 3.509260 | -2.944060 | -1.357770 |
| 39 | 1 | 0 |  | 2.620710 | -3.580390 | -1.439760 |
| 40 | 1 | 0 |  | 3.446100 | -2.180310 | -2.131080 |
| 41 | 1 | 0 |  | 4.371640 | -3.584050 | -1.577380 |
| 42 | 8 | 0 |  | -2.067330 | -1.147211 | 2.781730 |
| 43 | 1 | 0 |  | -1.330391 | -0.536261 | 2.970060 |
| 44 | 6 | 0 |  | -4.969280 | -2.362192 | -0.976410 |
| 45 | 1 | 0 |  | -4.557160 | -3.209282 | -1.535420 |
| 46 | 1 | 0 |  | -5.619210 | -2.763562 | -0.197610 |
| 47 | 1 | 0 |  | -5.576060 | -1.779672 | -1.672990 |
| **Conformer f-1** | | |  |  |  |  |
| Center | Atomic | Atomic |  | Coordinates (Angstroms) | | |
| Number | Number | Type |  | X | Y | Z |
| 1 | 6 | 0 |  | -4.324330 | -1.648530 | -0.118820 |
| 2 | 1 | 0 |  | -4.980860 | -2.508710 | -0.059260 |
| 3 | 6 | 0 |  | -4.640010 | -0.574110 | -0.960580 |
| 4 | 6 | 0 |  | -3.795541 | 0.554650 | -1.031460 |
| 5 | 1 | 0 |  | -4.055101 | 1.391860 | -1.668530 |
| 6 | 6 | 0 |  | -2.644791 | 0.567110 | -0.270050 |
| 7 | 6 | 0 |  | -2.319090 | -0.517780 | 0.550460 |
| 8 | 6 | 0 |  | -3.161950 | -1.634350 | 0.654170 |
| 9 | 6 | 0 |  | -1.623661 | 1.665940 | -0.060720 |
| 10 | 1 | 0 |  | -2.028661 | 2.349840 | 0.693270 |
| 11 | 6 | 0 |  | -0.389871 | 0.938480 | 0.587080 |
| 12 | 6 | 0 |  | -1.036820 | -0.337850 | 1.195210 |
| 13 | 6 | 0 |  | 0.358759 | 1.842490 | 1.581010 |
| 14 | 1 | 0 |  | -0.330251 | 2.136170 | 2.382460 |
| 15 | 6 | 0 |  | 1.581909 | 1.156390 | 2.178470 |
| 16 | 1 | 0 |  | 1.275759 | 0.481350 | 2.983920 |
| 17 | 1 | 0 |  | 2.216459 | 1.920880 | 2.637890 |
| 18 | 6 | 0 |  | 2.376309 | 0.381710 | 1.173760 |
| 19 | 1 | 0 |  | 3.378550 | 0.086070 | 1.471380 |
| 20 | 6 | 0 |  | 1.949170 | 0.043380 | -0.055230 |
| 21 | 6 | 0 |  | 0.595479 | 0.431270 | -0.490920 |
| 22 | 8 | 0 |  | -0.539290 | -1.085590 | 2.029670 |
| 23 | 8 | 0 |  | 0.233609 | 0.314930 | -1.655930 |
| 24 | 8 | 0 |  | -1.328971 | 2.489980 | -1.172190 |
| 25 | 1 | 0 |  | -0.915191 | 1.910680 | -1.832090 |
| 26 | 8 | 0 |  | 0.828369 | 3.011160 | 0.894970 |
| 27 | 1 | 0 |  | 0.170259 | 3.280480 | 0.239540 |
| 28 | 6 | 0 |  | 2.795250 | -0.688980 | -1.085000 |
| 29 | 1 | 0 |  | 3.031090 | 0.008580 | -1.893210 |
| 30 | 1 | 0 |  | 2.162440 | -1.455830 | -1.547550 |
| 31 | 6 | 0 |  | 4.033300 | -1.341000 | -0.535310 |
| 32 | 1 | 0 |  | 3.845260 | -2.189830 | 0.120570 |
| 33 | 6 | 0 |  | 5.311120 | -1.011670 | -0.765260 |
| 34 | 6 | 0 |  | 6.436240 | -1.807250 | -0.148910 |
| 35 | 1 | 0 |  | 7.084700 | -2.230310 | -0.924990 |
| 36 | 1 | 0 |  | 7.072680 | -1.166460 | 0.472310 |
| 37 | 1 | 0 |  | 6.064690 | -2.626100 | 0.470230 |
| 38 | 6 | 0 |  | 5.761990 | 0.137790 | -1.630910 |
| 39 | 1 | 0 |  | 6.374140 | -0.227040 | -2.463690 |
| 40 | 1 | 0 |  | 4.937839 | 0.717830 | -2.045050 |
| 41 | 1 | 0 |  | 6.397719 | 0.818950 | -1.054140 |
| 42 | 8 | 0 |  | -2.859530 | -2.672620 | 1.459510 |
| 43 | 1 | 0 |  | -2.020990 | -2.465970 | 1.913270 |
| 44 | 6 | 0 |  | -5.886920 | -0.623800 | -1.806390 |
| 45 | 1 | 0 |  | -5.628630 | -0.819080 | -2.852490 |
| 46 | 1 | 0 |  | -6.566810 | -1.410300 | -1.476740 |
| 47 | 1 | 0 |  | -6.417831 | 0.330750 | -1.778930 |
| **Conformer f-2** | | |  |  |  |  |
| Center | Atomic | Atomic |  | Coordinates (Angstroms) | | |
| Number | Number | Type |  | X | Y | Z |
| 1 | 6 | 0 |  | 4.382700 | -0.738431 | -0.913450 |
| 2 | 1 | 0 |  | 5.245490 | -0.843461 | -1.560860 |
| 3 | 6 | 0 |  | 4.209750 | -1.596591 | 0.181120 |
| 4 | 6 | 0 |  | 3.092900 | -1.459240 | 1.031730 |
| 5 | 1 | 0 |  | 2.970850 | -2.119770 | 1.882110 |
| 6 | 6 | 0 |  | 2.168430 | -0.469290 | 0.760180 |
| 7 | 6 | 0 |  | 2.334210 | 0.369360 | -0.344000 |
| 8 | 6 | 0 |  | 3.448000 | 0.259550 | -1.191390 |
| 9 | 6 | 0 |  | 0.930180 | -0.068070 | 1.541400 |
| 10 | 1 | 0 |  | 1.237161 | 0.499590 | 2.423160 |
| 11 | 6 | 0 |  | 0.166251 | 0.936721 | 0.590310 |
| 12 | 6 | 0 |  | 1.227781 | 1.283840 | -0.493980 |
| 13 | 6 | 0 |  | -0.324049 | 2.196651 | 1.346720 |
| 14 | 1 | 0 |  | 0.548221 | 2.748870 | 1.702570 |
| 15 | 6 | 0 |  | -1.175359 | 3.104961 | 0.458250 |
| 16 | 1 | 0 |  | -0.532169 | 3.662961 | -0.230200 |
| 17 | 1 | 0 |  | -1.674979 | 3.844151 | 1.090970 |
| 18 | 6 | 0 |  | -2.187289 | 2.349251 | -0.343260 |
| 19 | 1 | 0 |  | -3.025029 | 2.922281 | -0.733140 |
| 20 | 6 | 0 |  | -2.128779 | 1.032761 | -0.621040 |
| 21 | 6 | 0 |  | -0.977610 | 0.229961 | -0.165670 |
| 22 | 8 | 0 |  | 1.134551 | 2.163750 | -1.344470 |
| 23 | 8 | 0 |  | -0.885720 | -0.964749 | -0.424580 |
| 24 | 8 | 0 |  | 0.178920 | -1.149790 | 2.049290 |
| 25 | 1 | 0 |  | -0.156320 | -1.629509 | 1.276850 |
| 26 | 8 | 0 |  | -1.018399 | 1.829891 | 2.539340 |
| 27 | 1 | 0 |  | -1.829419 | 1.366511 | 2.297180 |
| 28 | 6 | 0 |  | -3.211700 | 0.315031 | -1.404560 |
| 29 | 1 | 0 |  | -2.747290 | -0.384509 | -2.099570 |
| 30 | 1 | 0 |  | -3.742789 | 1.062892 | -2.003850 |
| 31 | 6 | 0 |  | -4.204080 | -0.384738 | -0.504360 |
| 32 | 1 | 0 |  | -4.721050 | 0.280862 | 0.185730 |
| 33 | 6 | 0 |  | -4.522100 | -1.684688 | -0.467470 |
| 34 | 6 | 0 |  | -5.569090 | -2.192098 | 0.494910 |
| 35 | 1 | 0 |  | -6.387620 | -2.684818 | -0.042910 |
| 36 | 1 | 0 |  | -5.145170 | -2.943268 | 1.171600 |
| 37 | 1 | 0 |  | -5.992300 | -1.386688 | 1.098360 |
| 38 | 6 | 0 |  | -3.909050 | -2.748968 | -1.341060 |
| 39 | 1 | 0 |  | -4.671341 | -3.200268 | -1.987000 |
| 40 | 1 | 0 |  | -3.102260 | -2.376879 | -1.969920 |
| 41 | 1 | 0 |  | -3.506461 | -3.559078 | -0.722560 |
| 42 | 8 | 0 |  | 3.609621 | 1.079730 | -2.249430 |
| 43 | 1 | 0 |  | 2.854631 | 1.698230 | -2.264700 |
| 44 | 6 | 0 |  | 5.217860 | -2.685741 | 0.447280 |
| 45 | 1 | 0 |  | 4.807279 | -3.661051 | 0.165640 |
| 46 | 1 | 0 |  | 6.136390 | -2.531911 | -0.120170 |
| 47 | 1 | 0 |  | 5.468650 | -2.737181 | 1.509560 |
| **Conformer f-3** | | |  |  |  |  |
| Center | Atomic | Atomic |  | Coordinates (Angstroms) | | |
| Number | Number | Type |  | X | Y | Z |
| 1 | 6 | 0 |  | -4.039871 | -1.916739 | 0.214310 |
| 2 | 1 | 0 |  | -4.588091 | -2.833259 | 0.398830 |
| 3 | 6 | 0 |  | -4.522050 | -0.977429 | -0.707010 |
| 4 | 6 | 0 |  | -3.821000 | 0.224641 | -0.940830 |
| 5 | 1 | 0 |  | -4.210110 | 0.957041 | -1.638150 |
| 6 | 6 | 0 |  | -2.640990 | 0.444361 | -0.259010 |
| 7 | 6 | 0 |  | -2.148580 | -0.506689 | 0.640330 |
| 8 | 6 | 0 |  | -2.848530 | -1.693239 | 0.906250 |
| 9 | 6 | 0 |  | -1.745480 | 1.665641 | -0.222350 |
| 10 | 1 | 0 |  | -2.187690 | 2.374961 | 0.485510 |
| 11 | 6 | 0 |  | -0.403700 | 1.150910 | 0.414920 |
| 12 | 6 | 0 |  | -0.864070 | -0.120450 | 1.181430 |
| 13 | 6 | 0 |  | 0.282690 | 2.230910 | 1.268240 |
| 14 | 1 | 0 |  | -0.394980 | 2.529150 | 2.077810 |
| 15 | 6 | 0 |  | 1.605120 | 1.750360 | 1.854760 |
| 16 | 1 | 0 |  | 1.420350 | 1.136280 | 2.741860 |
| 17 | 1 | 0 |  | 2.169990 | 2.624460 | 2.194380 |
| 18 | 6 | 0 |  | 2.431520 | 0.963540 | 0.886000 |
| 19 | 1 | 0 |  | 3.469550 | 0.795979 | 1.158970 |
| 20 | 6 | 0 |  | 1.989210 | 0.463940 | -0.281160 |
| 21 | 6 | 0 |  | 0.578680 | 0.649490 | -0.668970 |
| 22 | 8 | 0 |  | -0.239140 | -0.719120 | 2.049790 |
| 23 | 8 | 0 |  | 0.171810 | 0.368900 | -1.790270 |
| 24 | 8 | 0 |  | -1.605630 | 2.397350 | -1.424990 |
| 25 | 1 | 0 |  | -1.160040 | 1.802550 | -2.049420 |
| 26 | 8 | 0 |  | 0.579391 | 3.365860 | 0.443580 |
| 27 | 1 | 0 |  | -0.139779 | 3.494520 | -0.190050 |
| 28 | 6 | 0 |  | 2.854460 | -0.294361 | -1.274750 |
| 29 | 1 | 0 |  | 2.687590 | 0.157820 | -2.259570 |
| 30 | 1 | 0 |  | 2.466150 | -1.313090 | -1.362960 |
| 31 | 6 | 0 |  | 4.326710 | -0.287191 | -0.973180 |
| 32 | 1 | 0 |  | 4.811700 | 0.679829 | -1.098160 |
| 33 | 6 | 0 |  | 5.094070 | -1.315761 | -0.589170 |
| 34 | 6 | 0 |  | 6.575270 | -1.132141 | -0.363930 |
| 35 | 1 | 0 |  | 6.850130 | -1.397791 | 0.663380 |
| 36 | 1 | 0 |  | 7.153549 | -1.792372 | -1.020870 |
| 37 | 1 | 0 |  | 6.891410 | -0.103541 | -0.548430 |
| 38 | 6 | 0 |  | 4.597069 | -2.719431 | -0.351370 |
| 39 | 1 | 0 |  | 4.846079 | -3.041611 | 0.666060 |
| 40 | 1 | 0 |  | 3.520889 | -2.826791 | -0.483300 |
| 41 | 1 | 0 |  | 5.095169 | -3.422251 | -1.029210 |
| 42 | 8 | 0 |  | -2.385121 | -2.601389 | 1.788680 |
| 43 | 1 | 0 |  | -1.554141 | -2.256350 | 2.165820 |
| 44 | 6 | 0 |  | -5.794260 | -1.258239 | -1.465560 |
| 45 | 1 | 0 |  | -5.562700 | -1.607819 | -2.477370 |
| 46 | 1 | 0 |  | -6.392541 | -2.027888 | -0.976240 |
| 47 | 1 | 0 |  | -6.400570 | -0.355368 | -1.566880 |
| **Conformer f-4** | | |  |  |  |  |
| Center | Atomic | Atomic |  | Coordinates (Angstroms) | | |
| Number | Number | Type |  | X | Y | Z |
| 1 | 6 | 0 |  | 3.558561 | 1.451769 | 0.791251 |
| 2 | 1 | 0 |  | 4.127331 | 2.233759 | 1.280801 |
| 3 | 6 | 0 |  | 3.937720 | 0.988479 | -0.475649 |
| 4 | 6 | 0 |  | 3.208920 | -0.038771 | -1.112029 |
| 5 | 1 | 0 |  | 3.518000 | -0.407271 | -2.082989 |
| 6 | 6 | 0 |  | 2.105320 | -0.561390 | -0.468899 |
| 7 | 6 | 0 |  | 1.714660 | -0.080460 | 0.785081 |
| 8 | 6 | 0 |  | 2.444510 | 0.920530 | 1.443411 |
| 9 | 6 | 0 |  | 1.209790 | -1.721810 | -0.850629 |
| 10 | 1 | 0 |  | 1.715579 | -2.641910 | -0.538289 |
| 11 | 6 | 0 |  | -0.057250 | -1.556600 | 0.063851 |
| 12 | 6 | 0 |  | 0.489690 | -0.701230 | 1.240191 |
| 13 | 6 | 0 |  | -0.670701 | -2.914359 | 0.448861 |
| 14 | 1 | 0 |  | 0.080679 | -3.502760 | 0.989891 |
| 15 | 6 | 0 |  | -1.922841 | -2.764479 | 1.305741 |
| 16 | 1 | 0 |  | -1.643141 | -2.572519 | 2.346651 |
| 17 | 1 | 0 |  | -2.462471 | -3.716759 | 1.298841 |
| 18 | 6 | 0 |  | -2.826790 | -1.666019 | 0.841121 |
| 19 | 1 | 0 |  | -3.833890 | -1.665508 | 1.251201 |
| 20 | 6 | 0 |  | -2.491620 | -0.700899 | -0.033119 |
| 21 | 6 | 0 |  | -1.136170 | -0.679769 | -0.615519 |
| 22 | 8 | 0 |  | -0.039230 | -0.534800 | 2.333711 |
| 23 | 8 | 0 |  | -0.849330 | 0.025131 | -1.574559 |
| 24 | 8 | 0 |  | 0.945819 | -1.906150 | -2.228329 |
| 25 | 1 | 0 |  | 0.441490 | -1.125680 | -2.509469 |
| 26 | 8 | 0 |  | -1.061931 | -3.608319 | -0.743429 |
| 27 | 1 | 0 |  | -0.410181 | -3.437260 | -1.437489 |
| 28 | 6 | 0 |  | -3.452030 | 0.388151 | -0.471429 |
| 29 | 1 | 0 |  | -4.467670 | 0.062642 | -0.221389 |
| 30 | 1 | 0 |  | -3.404860 | 0.490441 | -1.556109 |
| 31 | 6 | 0 |  | -3.171139 | 1.704731 | 0.215371 |
| 32 | 1 | 0 |  | -3.236629 | 1.659931 | 1.301671 |
| 33 | 6 | 0 |  | -2.863209 | 2.884361 | -0.338159 |
| 34 | 6 | 0 |  | -2.629809 | 4.100371 | 0.526031 |
| 35 | 1 | 0 |  | -1.622208 | 4.502921 | 0.368881 |
| 36 | 1 | 0 |  | -3.329268 | 4.903791 | 0.266681 |
| 37 | 1 | 0 |  | -2.746589 | 3.874201 | 1.587701 |
| 38 | 6 | 0 |  | -2.720229 | 3.134961 | -1.817569 |
| 39 | 1 | 0 |  | -1.726029 | 3.540181 | -2.037689 |
| 40 | 1 | 0 |  | -2.857589 | 2.240421 | -2.422589 |
| 41 | 1 | 0 |  | -3.443689 | 3.889021 | -2.148839 |
| 42 | 8 | 0 |  | 2.079801 | 1.373350 | 2.660081 |
| 43 | 1 | 0 |  | 1.289750 | 0.875050 | 2.941261 |
| 44 | 6 | 0 |  | 5.126691 | 1.599379 | -1.172539 |
| 45 | 1 | 0 |  | 4.794471 | 2.301559 | -1.944619 |
| 46 | 1 | 0 |  | 5.763991 | 2.146018 | -0.476399 |
| 47 | 1 | 0 |  | 5.727450 | 0.834358 | -1.669649 |
| **Conformer g-1** | | |  |  |  |  |
| Center | Atomic | Atomic |  | Coordinates (Angstroms) | | |
| Number | Number | Type |  | X | Y | Z |
| 1 | 6 | 0 |  | 4.481839 | -0.573460 | -1.065790 |
| 2 | 1 | 0 |  | 5.372289 | -0.502651 | -1.679600 |
| 3 | 6 | 0 |  | 4.433519 | -1.469560 | 0.011050 |
| 4 | 6 | 0 |  | 3.274329 | -1.571390 | 0.807260 |
| 5 | 1 | 0 |  | 3.242749 | -2.270200 | 1.634890 |
| 6 | 6 | 0 |  | 2.190029 | -0.768050 | 0.507610 |
| 7 | 6 | 0 |  | 2.249510 | 0.133880 | -0.553900 |
| 8 | 6 | 0 |  | 3.390170 | 0.242130 | -1.364250 |
| 9 | 6 | 0 |  | 0.835179 | -0.706450 | 1.177360 |
| 10 | 1 | 0 |  | 0.287569 | -1.631900 | 0.989120 |
| 11 | 6 | 0 |  | 0.073280 | 0.477390 | 0.470060 |
| 12 | 6 | 0 |  | 1.036100 | 0.908930 | -0.669250 |
| 13 | 6 | 0 |  | -0.196420 | 1.652740 | 1.448560 |
| 14 | 1 | 0 |  | -0.820660 | 1.250730 | 2.256450 |
| 15 | 6 | 0 |  | -0.961140 | 2.783980 | 0.778890 |
| 16 | 1 | 0 |  | -0.289090 | 3.343190 | 0.118390 |
| 17 | 1 | 0 |  | -1.300100 | 3.489480 | 1.543640 |
| 18 | 6 | 0 |  | -2.130510 | 2.285260 | -0.008020 |
| 19 | 1 | 0 |  | -2.897680 | 3.016671 | -0.249790 |
| 20 | 6 | 0 |  | -2.298250 | 1.017380 | -0.425740 |
| 21 | 6 | 0 |  | -1.248150 | 0.005680 | -0.177670 |
| 22 | 8 | 0 |  | 0.812950 | 1.757300 | -1.526970 |
| 23 | 8 | 0 |  | -1.378891 | -1.158390 | -0.522320 |
| 24 | 8 | 0 |  | 1.016169 | -0.530720 | 2.587100 |
| 25 | 1 | 0 |  | 0.257469 | -0.905930 | 3.047410 |
| 26 | 8 | 0 |  | 1.008550 | 2.177480 | 1.985620 |
| 27 | 1 | 0 |  | 1.357810 | 1.480700 | 2.559960 |
| 28 | 6 | 0 |  | -3.542340 | 0.559201 | -1.163430 |
| 29 | 1 | 0 |  | -3.255050 | -0.137939 | -1.950490 |
| 30 | 1 | 0 |  | -3.988230 | 1.434261 | -1.649480 |
| 31 | 6 | 0 |  | -4.563630 | -0.057099 | -0.234950 |
| 32 | 1 | 0 |  | -4.908310 | 0.614791 | 0.550140 |
| 33 | 6 | 0 |  | -5.083971 | -1.290149 | -0.267300 |
| 34 | 6 | 0 |  | -6.116911 | -1.715579 | 0.748690 |
| 35 | 1 | 0 |  | -7.047981 | -2.019229 | 0.255770 |
| 36 | 1 | 0 |  | -5.768941 | -2.585869 | 1.317580 |
| 37 | 1 | 0 |  | -6.349051 | -0.915469 | 1.454440 |
| 38 | 6 | 0 |  | -4.723541 | -2.354449 | -1.271970 |
| 39 | 1 | 0 |  | -5.607511 | -2.651709 | -1.848290 |
| 40 | 1 | 0 |  | -3.948131 | -2.043839 | -1.969940 |
| 41 | 1 | 0 |  | -4.370921 | -3.255539 | -0.757270 |
| 42 | 8 | 0 |  | 3.437670 | 1.110010 | -2.395170 |
| 43 | 1 | 0 |  | 2.582550 | 1.581010 | -2.425270 |
| 44 | 6 | 0 |  | 5.638309 | -2.316861 | 0.334210 |
| 45 | 1 | 0 |  | 5.343139 | -3.329220 | 0.619120 |
| 46 | 1 | 0 |  | 6.324729 | -2.378451 | -0.511340 |
| 47 | 1 | 0 |  | 6.187359 | -1.889471 | 1.179990 |
| **Conformer g-2** | | |  |  |  |  |
| Center | Atomic | Atomic |  | Coordinates (Angstroms) | | |
| Number | Number | Type |  | X | Y | Z |
| 1 | 6 | 0 |  | 4.599580 | 1.387170 | -0.300980 |
| 2 | 1 | 0 |  | 5.356630 | 2.161670 | -0.256310 |
| 3 | 6 | 0 |  | 4.935260 | 0.092490 | -0.722830 |
| 4 | 6 | 0 |  | 3.953160 | -0.915710 | -0.791700 |
| 5 | 1 | 0 |  | 4.219170 | -1.912100 | -1.124020 |
| 6 | 6 | 0 |  | 2.654700 | -0.604440 | -0.431570 |
| 7 | 6 | 0 |  | 2.329910 | 0.678940 | 0.004000 |
| 8 | 6 | 0 |  | 3.292180 | 1.699240 | 0.069680 |
| 9 | 6 | 0 |  | 1.424660 | -1.483850 | -0.448270 |
| 10 | 1 | 0 |  | 1.150340 | -1.718130 | -1.478700 |
| 11 | 6 | 0 |  | 0.278500 | -0.609410 | 0.185890 |
| 12 | 6 | 0 |  | 0.937890 | 0.783540 | 0.376520 |
| 13 | 6 | 0 |  | -0.201990 | -1.192200 | 1.543390 |
| 14 | 1 | 0 |  | -0.586900 | -2.197780 | 1.332750 |
| 15 | 6 | 0 |  | -1.330740 | -0.370410 | 2.146730 |
| 16 | 1 | 0 |  | -0.925160 | 0.556310 | 2.568100 |
| 17 | 1 | 0 |  | -1.769160 | -0.923780 | 2.982660 |
| 18 | 6 | 0 |  | -2.390060 | -0.034350 | 1.145480 |
| 19 | 1 | 0 |  | -3.358080 | 0.257970 | 1.541660 |
| 20 | 6 | 0 |  | -2.229490 | -0.071250 | -0.188960 |
| 21 | 6 | 0 |  | -0.921700 | -0.444440 | -0.770900 |
| 22 | 8 | 0 |  | 0.382110 | 1.803400 | 0.771150 |
| 23 | 8 | 0 |  | -0.773160 | -0.577790 | -1.976060 |
| 24 | 8 | 0 |  | 1.703690 | -2.695150 | 0.263380 |
| 25 | 1 | 0 |  | 1.140490 | -3.396100 | -0.082780 |
| 26 | 8 | 0 |  | 0.855680 | -1.269580 | 2.487190 |
| 27 | 1 | 0 |  | 1.452260 | -1.952620 | 2.148170 |
| 28 | 6 | 0 |  | -3.329960 | 0.246630 | -1.187900 |
| 29 | 1 | 0 |  | -3.582640 | -0.671500 | -1.725510 |
| 30 | 1 | 0 |  | -2.898120 | 0.908020 | -1.948770 |
| 31 | 6 | 0 |  | -4.549350 | 0.902640 | -0.602980 |
| 32 | 1 | 0 |  | -4.383970 | 1.911630 | -0.227480 |
| 33 | 6 | 0 |  | -5.789790 | 0.406420 | -0.507140 |
| 34 | 6 | 0 |  | -6.907880 | 1.232280 | 0.081390 |
| 35 | 1 | 0 |  | -7.714750 | 1.371530 | -0.647760 |
| 36 | 1 | 0 |  | -7.354350 | 0.726850 | 0.945570 |
| 37 | 1 | 0 |  | -6.561750 | 2.217200 | 0.400670 |
| 38 | 6 | 0 |  | -6.204200 | -0.970480 | -0.961260 |
| 39 | 1 | 0 |  | -6.971680 | -0.902160 | -1.740980 |
| 40 | 1 | 0 |  | -5.378230 | -1.564520 | -1.351270 |
| 41 | 1 | 0 |  | -6.655690 | -1.524080 | -0.130220 |
| 42 | 8 | 0 |  | 2.968950 | 2.941200 | 0.484270 |
| 43 | 1 | 0 |  | 2.015960 | 2.943590 | 0.697730 |
| 44 | 6 | 0 |  | 6.364850 | -0.226840 | -1.080890 |
| 45 | 1 | 0 |  | 6.417520 | -0.996040 | -1.853700 |
| 46 | 1 | 0 |  | 6.897170 | 0.658900 | -1.431610 |
| 47 | 1 | 0 |  | 6.899580 | -0.607300 | -0.203800 |
| **Conformer g-3** | | |  |  |  |  |
| Center | Atomic | Atomic |  | Coordinates (Angstroms) | | |
| Number | Number | Type |  | X | Y | Z |
| 1 | 6 | 0 |  | 4.371680 | 1.697749 | 0.130490 |
| 2 | 1 | 0 |  | 5.035900 | 2.516229 | 0.383030 |
| 3 | 6 | 0 |  | 4.851810 | 0.591129 | -0.584320 |
| 4 | 6 | 0 |  | 3.990190 | -0.471261 | -0.924240 |
| 5 | 1 | 0 |  | 4.367640 | -1.319901 | -1.482360 |
| 6 | 6 | 0 |  | 2.665330 | -0.402911 | -0.534640 |
| 7 | 6 | 0 |  | 2.197330 | 0.690699 | 0.191920 |
| 8 | 6 | 0 |  | 3.037090 | 1.763699 | 0.529790 |
| 9 | 6 | 0 |  | 1.543580 | -1.382881 | -0.796530 |
| 10 | 1 | 0 |  | 1.301570 | -1.393421 | -1.860990 |
| 11 | 6 | 0 |  | 0.302760 | -0.817191 | -0.007580 |
| 12 | 6 | 0 |  | 0.802360 | 0.547979 | 0.539110 |
| 13 | 6 | 0 |  | -0.120980 | -1.761791 | 1.149730 |
| 14 | 1 | 0 |  | -0.385240 | -2.722251 | 0.689750 |
| 15 | 6 | 0 |  | -1.343020 | -1.240421 | 1.890960 |
| 16 | 1 | 0 |  | -1.051260 | -0.406821 | 2.539820 |
| 17 | 1 | 0 |  | -1.723170 | -2.026241 | 2.550830 |
| 18 | 6 | 0 |  | -2.423320 | -0.786591 | 0.961900 |
| 19 | 1 | 0 |  | -3.420310 | -0.695011 | 1.382710 |
| 20 | 6 | 0 |  | -2.247520 | -0.483001 | -0.336040 |
| 21 | 6 | 0 |  | -0.898060 | -0.545511 | -0.938810 |
| 22 | 8 | 0 |  | 0.134950 | 1.372249 | 1.155590 |
| 23 | 8 | 0 |  | -0.718730 | -0.339771 | -2.129630 |
| 24 | 8 | 0 |  | 1.957550 | -2.690121 | -0.382620 |
| 25 | 1 | 0 |  | 1.485150 | -3.346641 | -0.906040 |
| 26 | 8 | 0 |  | 0.929820 | -1.949591 | 2.085950 |
| 27 | 1 | 0 |  | 1.603450 | -2.461381 | 1.615270 |
| 28 | 6 | 0 |  | -3.363140 | -0.032311 | -1.264170 |
| 29 | 1 | 0 |  | -3.261890 | -0.603051 | -2.194660 |
| 30 | 1 | 0 |  | -3.171740 | 1.006189 | -1.549740 |
| 31 | 6 | 0 |  | -4.754920 | -0.211281 | -0.725540 |
| 32 | 1 | 0 |  | -5.078220 | -1.247631 | -0.637900 |
| 33 | 6 | 0 |  | -5.627180 | 0.738090 | -0.361890 |
| 34 | 6 | 0 |  | -7.007840 | 0.372460 | 0.126800 |
| 35 | 1 | 0 |  | -7.176360 | 0.752350 | 1.141160 |
| 36 | 1 | 0 |  | -7.778510 | 0.826930 | -0.506840 |
| 37 | 1 | 0 |  | -7.164360 | -0.707990 | 0.134150 |
| 38 | 6 | 0 |  | -5.351020 | 2.220179 | -0.398580 |
| 39 | 1 | 0 |  | -5.515750 | 2.660069 | 0.591430 |
| 40 | 1 | 0 |  | -4.335450 | 2.463889 | -0.709060 |
| 41 | 1 | 0 |  | -6.045980 | 2.723540 | -1.080730 |
| 42 | 8 | 0 |  | 2.574600 | 2.823389 | 1.223760 |
| 43 | 1 | 0 |  | 1.627020 | 2.668939 | 1.403270 |
| 44 | 6 | 0 |  | 6.307820 | 0.526589 | -0.971140 |
| 45 | 1 | 0 |  | 6.438200 | 0.043909 | -1.941900 |
| 46 | 1 | 0 |  | 6.756800 | 1.520149 | -1.011630 |
| 47 | 1 | 0 |  | 6.869270 | -0.061641 | -0.237240 |
| **Conformer g-4** | | |  |  |  |  |
| Center | Atomic | Atomic |  | Coordinates (Angstroms) | | |
| Number | Number | Type |  | X | Y | Z |
| 1 | 6 | 0 |  | -3.710630 | -1.870209 | 0.745531 |
| 2 | 1 | 0 |  | -4.287790 | -2.588429 | 1.316201 |
| 3 | 6 | 0 |  | -4.178940 | -1.408949 | -0.492299 |
| 4 | 6 | 0 |  | -3.427739 | -0.480539 | -1.242099 |
| 5 | 1 | 0 |  | -3.794419 | -0.131999 | -2.200489 |
| 6 | 6 | 0 |  | -2.224029 | -0.032869 | -0.731289 |
| 7 | 6 | 0 |  | -1.770659 | -0.481999 | 0.508461 |
| 8 | 6 | 0 |  | -2.499090 | -1.412789 | 1.264911 |
| 9 | 6 | 0 |  | -1.226899 | 0.926421 | -1.342209 |
| 10 | 1 | 0 |  | -0.765719 | 0.473360 | -2.221669 |
| 11 | 6 | 0 |  | -0.115389 | 1.127900 | -0.245099 |
| 12 | 6 | 0 |  | -0.520009 | 0.138380 | 0.880631 |
| 13 | 6 | 0 |  | -0.085389 | 2.590940 | 0.279111 |
| 14 | 1 | 0 |  | 0.134361 | 3.227710 | -0.586959 |
| 15 | 6 | 0 |  | 1.007751 | 2.805940 | 1.314911 |
| 16 | 1 | 0 |  | 0.697961 | 2.367910 | 2.270501 |
| 17 | 1 | 0 |  | 1.127911 | 3.879030 | 1.491341 |
| 18 | 6 | 0 |  | 2.311971 | 2.203540 | 0.901101 |
| 19 | 1 | 0 |  | 3.197231 | 2.570000 | 1.414801 |
| 20 | 6 | 0 |  | 2.463241 | 1.253120 | -0.038319 |
| 21 | 6 | 0 |  | 1.284281 | 0.722580 | -0.758669 |
| 22 | 8 | 0 |  | 0.108261 | -0.089890 | 1.909391 |
| 23 | 8 | 0 |  | 1.397561 | -0.034210 | -1.709699 |
| 24 | 8 | 0 |  | -1.905409 | 2.130421 | -1.721559 |
| 25 | 1 | 0 |  | -1.432349 | 2.535681 | -2.456599 |
| 26 | 8 | 0 |  | -1.328499 | 2.968121 | 0.851311 |
| 27 | 1 | 0 |  | -1.951229 | 3.000321 | 0.110451 |
| 28 | 6 | 0 |  | 3.813591 | 0.660570 | -0.393569 |
| 29 | 1 | 0 |  | 4.588211 | 1.369960 | -0.082209 |
| 30 | 1 | 0 |  | 3.885111 | 0.557790 | -1.476579 |
| 31 | 6 | 0 |  | 4.052561 | -0.662010 | 0.298301 |
| 32 | 1 | 0 |  | 4.035271 | -0.601220 | 1.385671 |
| 33 | 6 | 0 |  | 4.280060 | -1.861710 | -0.250739 |
| 34 | 6 | 0 |  | 4.516650 | -3.072380 | 0.620191 |
| 35 | 1 | 0 |  | 3.770850 | -3.850130 | 0.417981 |
| 36 | 1 | 0 |  | 5.495750 | -3.519150 | 0.410511 |
| 37 | 1 | 0 |  | 4.474380 | -2.824510 | 1.682631 |
| 38 | 6 | 0 |  | 4.331020 | -2.139490 | -1.731399 |
| 39 | 1 | 0 |  | 3.618760 | -2.930720 | -1.991579 |
| 40 | 1 | 0 |  | 4.098340 | -1.268170 | -2.340939 |
| 41 | 1 | 0 |  | 5.323130 | -2.507830 | -2.017809 |
| 42 | 8 | 0 |  | -2.051450 | -1.849369 | 2.459601 |
| 43 | 1 | 0 |  | -1.199160 | -1.406309 | 2.636531 |
| 44 | 6 | 0 |  | -5.505570 | -1.894209 | -1.020149 |
| 45 | 1 | 0 |  | -5.445270 | -2.119199 | -2.087569 |
| 46 | 1 | 0 |  | -5.845030 | -2.787489 | -0.494409 |
| 47 | 1 | 0 |  | -6.270690 | -1.120619 | -0.895959 |
| **Conformer h-1** | | |  |  |  |  |
| Center | Atomic | Atomic |  | Coordinates (Angstroms) | | |
| Number | Number | Type |  | X | Y | Z |
| 1 | 6 | 0 |  | -4.382480 | 0.855910 | -0.927190 |
| 2 | 1 | 0 |  | -5.250750 | 0.990380 | -1.561760 |
| 3 | 6 | 0 |  | -4.253300 | 1.587920 | 0.260650 |
| 4 | 6 | 0 |  | -3.128270 | 1.411260 | 1.093070 |
| 5 | 1 | 0 |  | -3.045060 | 1.972170 | 2.016770 |
| 6 | 6 | 0 |  | -2.151180 | 0.512990 | 0.710060 |
| 7 | 6 | 0 |  | -2.284730 | -0.212550 | -0.474270 |
| 8 | 6 | 0 |  | -3.400800 | -0.057780 | -1.311650 |
| 9 | 6 | 0 |  | -0.889700 | 0.107220 | 1.442580 |
| 10 | 1 | 0 |  | -0.263870 | 0.979110 | 1.649550 |
| 11 | 6 | 0 |  | -0.131910 | -0.852430 | 0.442960 |
| 12 | 6 | 0 |  | -1.162990 | -1.095000 | -0.695710 |
| 13 | 6 | 0 |  | 0.327240 | -2.168900 | 1.112710 |
| 14 | 1 | 0 |  | -0.549360 | -2.719260 | 1.451930 |
| 15 | 6 | 0 |  | 1.162070 | -3.032620 | 0.172200 |
| 16 | 1 | 0 |  | 0.513201 | -3.512040 | -0.567710 |
| 17 | 1 | 0 |  | 1.623361 | -3.836830 | 0.752800 |
| 18 | 6 | 0 |  | 2.216570 | -2.245500 | -0.543490 |
| 19 | 1 | 0 |  | 3.046640 | -2.810850 | -0.959820 |
| 20 | 6 | 0 |  | 2.201730 | -0.909830 | -0.708050 |
| 21 | 6 | 0 |  | 1.053820 | -0.111270 | -0.220320 |
| 22 | 8 | 0 |  | -1.039430 | -1.872750 | -1.636860 |
| 23 | 8 | 0 |  | 0.994770 | 1.096650 | -0.380720 |
| 24 | 8 | 0 |  | -1.278430 | -0.528630 | 2.655170 |
| 25 | 1 | 0 |  | -0.458430 | -0.849000 | 3.062160 |
| 26 | 8 | 0 |  | 1.050070 | -1.878500 | 2.324920 |
| 27 | 1 | 0 |  | 1.912750 | -1.509690 | 2.096980 |
| 28 | 6 | 0 |  | 3.324980 | -0.159230 | -1.397810 |
| 29 | 1 | 0 |  | 2.898310 | 0.612770 | -2.038100 |
| 30 | 1 | 0 |  | 3.853180 | -0.866490 | -2.046750 |
| 31 | 6 | 0 |  | 4.308200 | 0.429950 | -0.412460 |
| 32 | 1 | 0 |  | 4.798730 | -0.310480 | 0.218200 |
| 33 | 6 | 0 |  | 4.642350 | 1.714120 | -0.234480 |
| 34 | 6 | 0 |  | 5.669630 | 2.103110 | 0.801580 |
| 35 | 1 | 0 |  | 6.511810 | 2.630080 | 0.337890 |
| 36 | 1 | 0 |  | 5.239390 | 2.792560 | 1.537570 |
| 37 | 1 | 0 |  | 6.061080 | 1.234060 | 1.334010 |
| 38 | 6 | 0 |  | 4.067110 | 2.873040 | -1.007400 |
| 39 | 1 | 0 |  | 4.860620 | 3.404790 | -1.545210 |
| 40 | 1 | 0 |  | 3.304580 | 2.577270 | -1.725750 |
| 41 | 1 | 0 |  | 3.616830 | 3.598720 | -0.320520 |
| 42 | 8 | 0 |  | -3.528040 | -0.766990 | -2.451410 |
| 43 | 1 | 0 |  | -2.749350 | -1.351100 | -2.527030 |
| 44 | 6 | 0 |  | -5.315800 | 2.585670 | 0.646620 |
| 45 | 1 | 0 |  | -4.960860 | 3.606760 | 0.471900 |
| 46 | 1 | 0 |  | -6.229460 | 2.445160 | 0.067880 |
| 47 | 1 | 0 |  | -5.560160 | 2.507220 | 1.708690 |
| **Conformer h-2** | | |  |  |  |  |
| Center | Atomic | Atomic |  | Coordinates (Angstroms) | | |
| Number | Number | Type |  | X | Y | Z |
| 1 | 6 | 0 |  | -4.567729 | -1.470582 | -0.099670 |
| 2 | 1 | 0 |  | -5.311929 | -2.246542 | 0.036970 |
| 3 | 6 | 0 |  | -4.838110 | -0.365782 | -0.917220 |
| 4 | 6 | 0 |  | -3.871350 | 0.646319 | -1.097210 |
| 5 | 1 | 0 |  | -4.093230 | 1.501849 | -1.724790 |
| 6 | 6 | 0 |  | -2.652140 | 0.523329 | -0.460370 |
| 7 | 6 | 0 |  | -2.388799 | -0.578951 | 0.354050 |
| 8 | 6 | 0 |  | -3.339659 | -1.592201 | 0.552260 |
| 9 | 6 | 0 |  | -1.478590 | 1.480200 | -0.455180 |
| 10 | 1 | 0 |  | -1.135120 | 1.675920 | -1.474030 |
| 11 | 6 | 0 |  | -0.342580 | 0.723930 | 0.339270 |
| 12 | 6 | 0 |  | -1.061459 | -0.526750 | 0.920740 |
| 13 | 6 | 0 |  | 0.302210 | 1.599741 | 1.440310 |
| 14 | 1 | 0 |  | -0.457831 | 1.862820 | 2.174950 |
| 15 | 6 | 0 |  | 1.474840 | 0.900111 | 2.119310 |
| 16 | 1 | 0 |  | 1.102710 | 0.130061 | 2.802630 |
| 17 | 1 | 0 |  | 2.009970 | 1.631112 | 2.732310 |
| 18 | 6 | 0 |  | 2.413400 | 0.263592 | 1.139950 |
| 19 | 1 | 0 |  | 3.419270 | 0.053532 | 1.491500 |
| 20 | 6 | 0 |  | 2.094740 | -0.066198 | -0.124550 |
| 21 | 6 | 0 |  | 0.726080 | 0.176161 | -0.635780 |
| 22 | 8 | 0 |  | -0.582609 | -1.347030 | 1.697470 |
| 23 | 8 | 0 |  | 0.420090 | -0.084949 | -1.788150 |
| 24 | 8 | 0 |  | -1.903581 | 2.686690 | 0.169310 |
| 25 | 1 | 0 |  | -1.113531 | 3.245530 | 0.233230 |
| 26 | 8 | 0 |  | 0.707869 | 2.865111 | 0.883880 |
| 27 | 1 | 0 |  | 1.487959 | 2.729571 | 0.331240 |
| 28 | 6 | 0 |  | 3.067511 | -0.691718 | -1.110930 |
| 29 | 1 | 0 |  | 3.271570 | 0.037312 | -1.900130 |
| 30 | 1 | 0 |  | 2.540381 | -1.511058 | -1.613280 |
| 31 | 6 | 0 |  | 4.336211 | -1.221557 | -0.502930 |
| 32 | 1 | 0 |  | 4.204811 | -2.102577 | 0.123700 |
| 33 | 6 | 0 |  | 5.581841 | -0.752047 | -0.651320 |
| 34 | 6 | 0 |  | 6.750361 | -1.440486 | 0.011500 |
| 35 | 1 | 0 |  | 7.482451 | -1.769346 | -0.735330 |
| 36 | 1 | 0 |  | 7.278651 | -0.753746 | 0.682930 |
| 37 | 1 | 0 |  | 6.435461 | -2.311586 | 0.589210 |
| 38 | 6 | 0 |  | 5.953340 | 0.461163 | -1.466090 |
| 39 | 1 | 0 |  | 6.638840 | 0.184454 | -2.275360 |
| 40 | 1 | 0 |  | 5.094690 | 0.965933 | -1.907900 |
| 41 | 1 | 0 |  | 6.487540 | 1.186904 | -0.842560 |
| 42 | 8 | 0 |  | -3.082728 | -2.653021 | 1.343700 |
| 43 | 1 | 0 |  | -2.183708 | -2.542080 | 1.707990 |
| 44 | 6 | 0 |  | -6.173230 | -0.248332 | -1.607910 |
| 45 | 1 | 0 |  | -6.042470 | -0.153192 | -2.689640 |
| 46 | 1 | 0 |  | -6.805399 | -1.115133 | -1.413240 |
| 47 | 1 | 0 |  | -6.704740 | 0.646197 | -1.269500 |
| **Conformer h-3** | | |  |  |  |  |
| Center | Atomic | Atomic |  | Coordinates (Angstroms) | | |
| Number | Number | Type |  | X | Y | Z |
| 1 | 6 | 0 |  | -4.362760 | -1.719241 | 0.201820 |
| 2 | 1 | 0 |  | -5.022030 | -2.538411 | 0.464390 |
| 3 | 6 | 0 |  | -4.776450 | -0.743121 | -0.714450 |
| 4 | 6 | 0 |  | -3.924100 | 0.328259 | -1.054380 |
| 5 | 1 | 0 |  | -4.258930 | 1.085789 | -1.753530 |
| 6 | 6 | 0 |  | -2.670850 | 0.391339 | -0.476560 |
| 7 | 6 | 0 |  | -2.264480 | -0.583231 | 0.435450 |
| 8 | 6 | 0 |  | -3.100890 | -1.652641 | 0.793060 |
| 9 | 6 | 0 |  | -1.601660 | 1.451779 | -0.633520 |
| 10 | 1 | 0 |  | -1.317760 | 1.563499 | -1.683080 |
| 11 | 6 | 0 |  | -0.365250 | 0.905499 | 0.183510 |
| 12 | 6 | 0 |  | -0.928740 | -0.339001 | 0.927200 |
| 13 | 6 | 0 |  | 0.220089 | 1.959110 | 1.153500 |
| 14 | 1 | 0 |  | -0.538911 | 2.225559 | 1.887940 |
| 15 | 6 | 0 |  | 1.481420 | 1.460660 | 1.851750 |
| 16 | 1 | 0 |  | 1.214110 | 0.736110 | 2.627700 |
| 17 | 1 | 0 |  | 1.956399 | 2.304810 | 2.360070 |
| 18 | 6 | 0 |  | 2.448520 | 0.817720 | 0.904690 |
| 19 | 1 | 0 |  | 3.479930 | 0.738900 | 1.235660 |
| 20 | 6 | 0 |  | 2.125310 | 0.326180 | -0.304930 |
| 21 | 6 | 0 |  | 0.720870 | 0.362530 | -0.773750 |
| 22 | 8 | 0 |  | -0.338240 | -1.018031 | 1.761320 |
| 23 | 8 | 0 |  | 0.402790 | -0.067620 | -1.870890 |
| 24 | 8 | 0 |  | -2.124831 | 2.673479 | -0.124010 |
| 25 | 1 | 0 |  | -1.394891 | 3.311089 | -0.159480 |
| 26 | 8 | 0 |  | 0.473109 | 3.189550 | 0.447530 |
| 27 | 1 | 0 |  | 1.242119 | 3.072230 | -0.124490 |
| 28 | 6 | 0 |  | 3.120050 | -0.325540 | -1.250990 |
| 29 | 1 | 0 |  | 2.927380 | 0.076100 | -2.252550 |
| 30 | 1 | 0 |  | 2.876730 | -1.389420 | -1.322890 |
| 31 | 6 | 0 |  | 4.565650 | -0.107970 | -0.901380 |
| 32 | 1 | 0 |  | 4.910450 | 0.918660 | -1.017540 |
| 33 | 6 | 0 |  | 5.460600 | -1.013840 | -0.485810 |
| 34 | 6 | 0 |  | 6.890960 | -0.615640 | -0.213790 |
| 35 | 1 | 0 |  | 7.168940 | -0.840790 | 0.822380 |
| 36 | 1 | 0 |  | 7.580630 | -1.181990 | -0.850540 |
| 37 | 1 | 0 |  | 7.057450 | 0.448700 | -0.390480 |
| 38 | 6 | 0 |  | 5.165150 | -2.474220 | -0.254760 |
| 39 | 1 | 0 |  | 5.417900 | -2.753600 | 0.774340 |
| 40 | 1 | 0 |  | 4.122230 | -2.738540 | -0.427160 |
| 41 | 1 | 0 |  | 5.786620 | -3.098790 | -0.906840 |
| 42 | 8 | 0 |  | -2.705590 | -2.587941 | 1.680210 |
| 43 | 1 | 0 |  | -1.806830 | -2.352541 | 1.980300 |
| 44 | 6 | 0 |  | -6.137060 | -0.844751 | -1.356160 |
| 45 | 1 | 0 |  | -6.044390 | -1.175791 | -2.395830 |
| 46 | 1 | 0 |  | -6.776430 | -1.557111 | -0.833500 |
| 47 | 1 | 0 |  | -6.636870 | 0.126759 | -1.371080 |
| **Conformer h-4** | | |  |  |  |  |
| Center | Atomic | Atomic |  | Coordinates (Angstroms) | | |
| Number | Number | Type |  | X | Y | Z |
| 1 | 6 | 0 |  | -3.621182 | -1.852868 | 0.700630 |
| 2 | 1 | 0 |  | -4.168122 | -2.641178 | 1.204620 |
| 3 | 6 | 0 |  | -4.056432 | -1.371378 | -0.540740 |
| 4 | 6 | 0 |  | -3.346661 | -0.345798 | -1.200650 |
| 5 | 1 | 0 |  | -3.696121 | 0.026032 | -2.157050 |
| 6 | 6 | 0 |  | -2.212591 | 0.169051 | -0.604160 |
| 7 | 6 | 0 |  | -1.785161 | -0.312669 | 0.634130 |
| 8 | 6 | 0 |  | -2.480382 | -1.327679 | 1.309430 |
| 9 | 6 | 0 |  | -1.313160 | 1.292230 | -1.075670 |
| 10 | 1 | 0 |  | -0.917861 | 1.080730 | -2.072170 |
| 11 | 6 | 0 |  | -0.123490 | 1.320110 | -0.039350 |
| 12 | 6 | 0 |  | -0.582831 | 0.349120 | 1.084850 |
| 13 | 6 | 0 |  | 0.170190 | 2.744820 | 0.488920 |
| 14 | 1 | 0 |  | -0.708870 | 3.113390 | 1.016220 |
| 15 | 6 | 0 |  | 1.390120 | 2.786469 | 1.403760 |
| 16 | 1 | 0 |  | 1.128600 | 2.386989 | 2.389180 |
| 17 | 1 | 0 |  | 1.676331 | 3.831179 | 1.555870 |
| 18 | 6 | 0 |  | 2.548500 | 2.004489 | 0.864830 |
| 19 | 1 | 0 |  | 3.526000 | 2.239828 | 1.278490 |
| 20 | 6 | 0 |  | 2.460119 | 1.037299 | -0.066230 |
| 21 | 6 | 0 |  | 1.145959 | 0.677589 | -0.647480 |
| 22 | 8 | 0 |  | -0.012961 | 0.149450 | 2.152930 |
| 23 | 8 | 0 |  | 1.044119 | -0.128291 | -1.557320 |
| 24 | 8 | 0 |  | -2.084540 | 2.489361 | -1.087240 |
| 25 | 1 | 0 |  | -1.467290 | 3.200101 | -1.320080 |
| 26 | 8 | 0 |  | 0.320441 | 3.655140 | -0.617650 |
| 27 | 1 | 0 |  | 1.160331 | 3.480309 | -1.060550 |
| 28 | 6 | 0 |  | 3.664059 | 0.248508 | -0.544840 |
| 29 | 1 | 0 |  | 4.561519 | 0.842918 | -0.342310 |
| 30 | 1 | 0 |  | 3.599709 | 0.116788 | -1.624970 |
| 31 | 6 | 0 |  | 3.785158 | -1.082142 | 0.162260 |
| 32 | 1 | 0 |  | 3.914388 | -0.999452 | 1.240570 |
| 33 | 6 | 0 |  | 3.754148 | -2.312202 | -0.365500 |
| 34 | 6 | 0 |  | 3.908267 | -3.529192 | 0.514840 |
| 35 | 1 | 0 |  | 3.028387 | -4.178832 | 0.438970 |
| 36 | 1 | 0 |  | 4.767817 | -4.132903 | 0.200450 |
| 37 | 1 | 0 |  | 4.045287 | -3.259042 | 1.563740 |
| 38 | 6 | 0 |  | 3.574218 | -2.620522 | -1.829800 |
| 39 | 1 | 0 |  | 2.723747 | -3.297192 | -1.970550 |
| 40 | 1 | 0 |  | 3.398718 | -1.734992 | -2.437940 |
| 41 | 1 | 0 |  | 4.455017 | -3.142482 | -2.221870 |
| 42 | 8 | 0 |  | -2.065912 | -1.787749 | 2.507150 |
| 43 | 1 | 0 |  | -1.267102 | -1.286110 | 2.758890 |
| 44 | 6 | 0 |  | -5.297302 | -1.944328 | -1.177610 |
| 45 | 1 | 0 |  | -5.078762 | -2.324478 | -2.179560 |
| 46 | 1 | 0 |  | -5.715372 | -2.758037 | -0.584340 |
| 47 | 1 | 0 |  | -6.064802 | -1.172287 | -1.288280 |

**Table S5** Standard orientations of configurations **e** and **f** at B3LYP/6-31+G(d,p) level in gas phase for NMR calculations.

| **Conformer e-1** | | |  |  |  |  |
| --- | --- | --- | --- | --- | --- | --- |
| Center | Atomic | Atomic |  | Coordinates (Angstroms) | | |
| Number | Number | Type |  | X | Y | Z |
| 1 | 6 | 0 |  | 4.225860 | -0.998337 | -0.939540 |
| 2 | 1 | 0 |  | 5.076031 | -1.161667 | -1.594430 |
| 3 | 6 | 0 |  | 4.004551 | -1.837978 | 0.164690 |
| 4 | 6 | 0 |  | 2.907071 | -1.621788 | 1.029320 |
| 5 | 1 | 0 |  | 2.751991 | -2.263838 | 1.891050 |
| 6 | 6 | 0 |  | 2.040630 | -0.577149 | 0.755590 |
| 7 | 6 | 0 |  | 2.251100 | 0.242051 | -0.361820 |
| 8 | 6 | 0 |  | 3.354150 | 0.059412 | -1.214620 |
| 9 | 6 | 0 |  | 0.849140 | -0.070549 | 1.549880 |
| 10 | 1 | 0 |  | 1.220810 | 0.525991 | 2.390440 |
| 11 | 6 | 0 |  | 0.121889 | 0.938900 | 0.571680 |
| 12 | 6 | 0 |  | 1.208939 | 1.229091 | -0.497230 |
| 13 | 6 | 0 |  | -0.346771 | 2.221040 | 1.300600 |
| 14 | 1 | 0 |  | -0.942681 | 1.895370 | 2.161180 |
| 15 | 6 | 0 |  | -1.209462 | 3.127699 | 0.418950 |
| 16 | 1 | 0 |  | -0.571252 | 3.660720 | -0.299460 |
| 17 | 1 | 0 |  | -1.683802 | 3.891489 | 1.046290 |
| 18 | 6 | 0 |  | -2.249962 | 2.365819 | -0.342810 |
| 19 | 1 | 0 |  | -3.095142 | 2.940138 | -0.721150 |
| 20 | 6 | 0 |  | -2.208991 | 1.041829 | -0.598570 |
| 21 | 6 | 0 |  | -1.047480 | 0.238289 | -0.158330 |
| 22 | 8 | 0 |  | 1.187789 | 2.152181 | -1.321300 |
| 23 | 8 | 0 |  | -0.975910 | -0.965520 | -0.399240 |
| 24 | 8 | 0 |  | 0.040320 | -1.067550 | 2.132620 |
| 25 | 1 | 0 |  | -0.339279 | -1.587220 | 1.404000 |
| 26 | 8 | 0 |  | 0.749328 | 2.931741 | 1.873070 |
| 27 | 1 | 0 |  | 1.237768 | 3.375311 | 1.164310 |
| 28 | 6 | 0 |  | -3.316940 | 0.314238 | -1.338790 |
| 29 | 1 | 0 |  | -2.873650 | -0.413022 | -2.022030 |
| 30 | 1 | 0 |  | -3.853651 | 1.050718 | -1.951390 |
| 31 | 6 | 0 |  | -4.298370 | -0.350582 | -0.398220 |
| 32 | 1 | 0 |  | -4.820190 | 0.342297 | 0.263730 |
| 33 | 6 | 0 |  | -4.597239 | -1.656383 | -0.290350 |
| 34 | 6 | 0 |  | -5.632739 | -2.125723 | 0.705450 |
| 35 | 1 | 0 |  | -6.447719 | -2.661474 | 0.200480 |
| 36 | 1 | 0 |  | -5.193068 | -2.830493 | 1.423750 |
| 37 | 1 | 0 |  | -6.068059 | -1.294033 | 1.266890 |
| 38 | 6 | 0 |  | -3.973329 | -2.758572 | -1.110130 |
| 39 | 1 | 0 |  | -4.732938 | -3.251393 | -1.731980 |
| 40 | 1 | 0 |  | -3.166729 | -2.413162 | -1.757300 |
| 41 | 1 | 0 |  | -3.558338 | -3.532002 | -0.451150 |
| 42 | 8 | 0 |  | 3.563839 | 0.868002 | -2.275520 |
| 43 | 1 | 0 |  | 2.856759 | 1.545172 | -2.288990 |
| 44 | 6 | 0 |  | 4.942042 | -2.992537 | 0.427320 |
| 45 | 1 | 0 |  | 4.476822 | -3.939937 | 0.127900 |
| 46 | 1 | 0 |  | 5.876742 | -2.889456 | -0.129960 |
| 47 | 1 | 0 |  | 5.182112 | -3.072957 | 1.492370 |
| **Conformer e-2** | | |  |  |  |  |
| Center | Atomic | Atomic |  | Coordinates (Angstroms) | | |
| Number | Number | Type |  | X | Y | Z |
| 1 | 6 | 0 |  | -4.479310 | -0.366341 | 1.414971 |
| 2 | 1 | 0 |  | -5.216450 | -0.355681 | 2.211781 |
| 3 | 6 | 0 |  | -4.691890 | -1.132851 | 0.256991 |
| 4 | 6 | 0 |  | -3.742280 | -1.139701 | -0.790099 |
| 5 | 1 | 0 |  | -3.921070 | -1.720261 | -1.690029 |
| 6 | 6 | 0 |  | -2.586550 | -0.391181 | -0.644679 |
| 7 | 6 | 0 |  | -2.367470 | 0.352539 | 0.522821 |
| 8 | 6 | 0 |  | -3.315190 | 0.393689 | 1.561071 |
| 9 | 6 | 0 |  | -1.458130 | -0.145861 | -1.631259 |
| 10 | 1 | 0 |  | -1.791880 | 0.603739 | -2.357439 |
| 11 | 6 | 0 |  | -0.322820 | 0.541930 | -0.770189 |
| 12 | 6 | 0 |  | -1.086921 | 1.014880 | 0.495191 |
| 13 | 6 | 0 |  | 0.351229 | 1.710700 | -1.528009 |
| 14 | 1 | 0 |  | 0.672679 | 1.313460 | -2.497929 |
| 15 | 6 | 0 |  | 1.571969 | 2.274230 | -0.796969 |
| 16 | 1 | 0 |  | 1.240609 | 2.900040 | 0.043581 |
| 17 | 1 | 0 |  | 2.126639 | 2.930190 | -1.477949 |
| 18 | 6 | 0 |  | 2.473719 | 1.205050 | -0.259669 |
| 19 | 1 | 0 |  | 3.499029 | 1.493271 | -0.036219 |
| 20 | 6 | 0 |  | 2.102900 | -0.070170 | -0.023519 |
| 21 | 6 | 0 |  | 0.715410 | -0.501350 | -0.299409 |
| 22 | 8 | 0 |  | -0.670671 | 1.824220 | 1.333551 |
| 23 | 8 | 0 |  | 0.371750 | -1.671660 | -0.135259 |
| 24 | 8 | 0 |  | -1.070320 | -1.260850 | -2.401869 |
| 25 | 1 | 0 |  | -0.727620 | -1.926840 | -1.781869 |
| 26 | 8 | 0 |  | -0.585801 | 2.735290 | -1.855169 |
| 27 | 1 | 0 |  | -0.792991 | 3.235780 | -1.052409 |
| 28 | 6 | 0 |  | 3.035290 | -1.143339 | 0.517371 |
| 29 | 1 | 0 |  | 3.208220 | -1.881079 | -0.273609 |
| 30 | 1 | 0 |  | 2.487040 | -1.693130 | 1.293541 |
| 31 | 6 | 0 |  | 4.327410 | -0.629519 | 1.092541 |
| 32 | 1 | 0 |  | 4.215390 | -0.044769 | 2.006981 |
| 33 | 6 | 0 |  | 5.577070 | -0.825319 | 0.635911 |
| 34 | 6 | 0 |  | 6.767670 | -0.268949 | 1.382351 |
| 35 | 1 | 0 |  | 7.447750 | -1.074698 | 1.689601 |
| 36 | 1 | 0 |  | 7.353060 | 0.406322 | 0.744021 |
| 37 | 1 | 0 |  | 6.467370 | 0.281671 | 2.278401 |
| 38 | 6 | 0 |  | 5.929140 | -1.598809 | -0.612059 |
| 39 | 1 | 0 |  | 6.552660 | -2.468009 | -0.363899 |
| 40 | 1 | 0 |  | 5.056890 | -1.957479 | -1.161689 |
| 41 | 1 | 0 |  | 6.522740 | -0.975309 | -1.293389 |
| 42 | 8 | 0 |  | -3.108381 | 1.131529 | 2.672961 |
| 43 | 1 | 0 |  | -2.251021 | 1.594719 | 2.578381 |
| 44 | 6 | 0 |  | -5.942980 | -1.969562 | 0.132261 |
| 45 | 1 | 0 |  | -5.718750 | -3.025622 | 0.327831 |
| 46 | 1 | 0 |  | -6.712020 | -1.655492 | 0.842781 |
| 47 | 1 | 0 |  | -6.360490 | -1.909452 | -0.877849 |
| **Conformer e-3** | | |  |  |  |  |
| Center | Atomic | Atomic |  | Coordinates (Angstroms) | | |
| Number | Number | Type |  | X | Y | Z |
| 1 | 6 | 0 |  | -4.293170 | -1.550881 | 0.655870 |
| 2 | 1 | 0 |  | -4.947070 | -2.266181 | 1.145090 |
| 3 | 6 | 0 |  | -4.643890 | -0.998641 | -0.587900 |
| 4 | 6 | 0 |  | -3.804380 | -0.055361 | -1.222280 |
| 5 | 1 | 0 |  | -4.089870 | 0.381959 | -2.174090 |
| 6 | 6 | 0 |  | -2.616540 | 0.299299 | -0.604600 |
| 7 | 6 | 0 |  | -2.258990 | -0.273061 | 0.623280 |
| 8 | 6 | 0 |  | -3.097410 | -1.190201 | 1.282560 |
| 9 | 6 | 0 |  | -1.583160 | 1.335390 | -1.011220 |
| 10 | 1 | 0 |  | -1.959971 | 2.326909 | -0.736460 |
| 11 | 6 | 0 |  | -0.345920 | 1.050390 | -0.066940 |
| 12 | 6 | 0 |  | -0.965600 | 0.187280 | 1.064350 |
| 13 | 6 | 0 |  | 0.292409 | 2.356560 | 0.463220 |
| 14 | 1 | 0 |  | 0.504569 | 2.981180 | -0.412430 |
| 15 | 6 | 0 |  | 1.596769 | 2.118920 | 1.227220 |
| 16 | 1 | 0 |  | 1.371049 | 1.725340 | 2.228240 |
| 17 | 1 | 0 |  | 2.105799 | 3.078640 | 1.374940 |
| 18 | 6 | 0 |  | 2.507480 | 1.151540 | 0.535450 |
| 19 | 1 | 0 |  | 3.556930 | 1.172510 | 0.822680 |
| 20 | 6 | 0 |  | 2.118600 | 0.254030 | -0.393430 |
| 21 | 6 | 0 |  | 0.695350 | 0.162350 | -0.785250 |
| 22 | 8 | 0 |  | -0.441600 | -0.060670 | 2.157650 |
| 23 | 8 | 0 |  | 0.324950 | -0.630090 | -1.650950 |
| 24 | 8 | 0 |  | -1.311130 | 1.412940 | -2.392350 |
| 25 | 1 | 0 |  | -0.935930 | 0.555320 | -2.654990 |
| 26 | 8 | 0 |  | -0.635661 | 3.121290 | 1.230470 |
| 27 | 1 | 0 |  | -0.750751 | 2.696650 | 2.093130 |
| 28 | 6 | 0 |  | 3.058640 | -0.728860 | -1.074170 |
| 29 | 1 | 0 |  | 2.806270 | -0.737580 | -2.142280 |
| 30 | 1 | 0 |  | 2.814020 | -1.737450 | -0.723230 |
| 31 | 6 | 0 |  | 4.523180 | -0.423640 | -0.911440 |
| 32 | 1 | 0 |  | 4.858730 | 0.477631 | -1.427090 |
| 33 | 6 | 0 |  | 5.449950 | -1.130009 | -0.240080 |
| 34 | 6 | 0 |  | 6.896300 | -0.691559 | -0.228600 |
| 35 | 1 | 0 |  | 7.247310 | -0.516749 | 0.797240 |
| 36 | 1 | 0 |  | 7.544280 | -1.471149 | -0.650940 |
| 37 | 1 | 0 |  | 7.050220 | 0.225741 | -0.804140 |
| 38 | 6 | 0 |  | 5.175250 | -2.394229 | 0.538310 |
| 39 | 1 | 0 |  | 5.504720 | -2.280909 | 1.579400 |
| 40 | 1 | 0 |  | 4.120930 | -2.676420 | 0.551090 |
| 41 | 1 | 0 |  | 5.745730 | -3.234829 | 0.121510 |
| 42 | 8 | 0 |  | -2.757710 | -1.719591 | 2.477680 |
| 43 | 1 | 0 |  | -1.899850 | -1.334871 | 2.750700 |
| 44 | 6 | 0 |  | -5.924590 | -1.430911 | -1.261500 |
| 45 | 1 | 0 |  | -5.724110 | -2.229751 | -1.986670 |
| 46 | 1 | 0 |  | -6.650740 | -1.814551 | -0.539610 |
| 47 | 1 | 0 |  | -6.384700 | -0.602901 | -1.809110 |
| **Conformer e-4** | | |  |  |  |  |
| Center | Atomic | Atomic |  | Coordinates (Angstroms) | | |
| Number | Number | Type |  | X | Y | Z |
| 1 | 6 | 0 |  | -3.532060 | -1.670881 | 0.948661 |
| 2 | 1 | 0 |  | -4.068300 | -2.375441 | 1.576771 |
| 3 | 6 | 0 |  | -3.902190 | -1.487301 | -0.394409 |
| 4 | 6 | 0 |  | -3.217970 | -0.559771 | -1.212399 |
| 5 | 1 | 0 |  | -3.521420 | -0.407561 | -2.243669 |
| 6 | 6 | 0 |  | -2.159430 | 0.150219 | -0.671069 |
| 7 | 6 | 0 |  | -1.776430 | -0.056301 | 0.661091 |
| 8 | 6 | 0 |  | -2.466800 | -0.951651 | 1.497611 |
| 9 | 6 | 0 |  | -1.319970 | 1.259179 | -1.281579 |
| 10 | 1 | 0 |  | -1.892261 | 2.192299 | -1.230289 |
| 11 | 6 | 0 |  | -0.104930 | 1.426310 | -0.281979 |
| 12 | 6 | 0 |  | -0.623670 | 0.737790 | 1.008251 |
| 13 | 6 | 0 |  | 0.261969 | 2.913610 | -0.056719 |
| 14 | 1 | 0 |  | 0.409749 | 3.354360 | -1.049439 |
| 15 | 6 | 0 |  | 1.541359 | 3.102280 | 0.763061 |
| 16 | 1 | 0 |  | 1.325699 | 2.924800 | 1.826011 |
| 17 | 1 | 0 |  | 1.870389 | 4.144590 | 0.679191 |
| 18 | 6 | 0 |  | 2.641829 | 2.174090 | 0.349441 |
| 19 | 1 | 0 |  | 3.651689 | 2.454540 | 0.647381 |
| 20 | 6 | 0 |  | 2.471940 | 1.024240 | -0.334699 |
| 21 | 6 | 0 |  | 1.117120 | 0.608990 | -0.761669 |
| 22 | 8 | 0 |  | -0.136120 | 0.854770 | 2.139271 |
| 23 | 8 | 0 |  | 0.950270 | -0.378100 | -1.475959 |
| 24 | 8 | 0 |  | -0.993970 | 1.099349 | -2.643239 |
| 25 | 1 | 0 |  | -0.438550 | 0.303600 | -2.706389 |
| 26 | 8 | 0 |  | -0.828621 | 3.644760 | 0.499911 |
| 27 | 1 | 0 |  | -0.919811 | 3.404030 | 1.433551 |
| 28 | 6 | 0 |  | 3.622010 | 0.095950 | -0.681119 |
| 29 | 1 | 0 |  | 4.549740 | 0.682620 | -0.658639 |
| 30 | 1 | 0 |  | 3.494330 | -0.267930 | -1.702769 |
| 31 | 6 | 0 |  | 3.741710 | -1.054140 | 0.294941 |
| 32 | 1 | 0 |  | 3.936370 | -0.747240 | 1.323491 |
| 33 | 6 | 0 |  | 3.640610 | -2.371240 | 0.047341 |
| 34 | 6 | 0 |  | 3.812461 | -3.376300 | 1.163001 |
| 35 | 1 | 0 |  | 2.909091 | -3.989870 | 1.278171 |
| 36 | 1 | 0 |  | 4.635401 | -4.069329 | 0.942431 |
| 37 | 1 | 0 |  | 4.021120 | -2.893050 | 2.121781 |
| 38 | 6 | 0 |  | 3.361470 | -2.980520 | -1.304429 |
| 39 | 1 | 0 |  | 2.486861 | -3.640960 | -1.247219 |
| 40 | 1 | 0 |  | 3.162280 | -2.240880 | -2.080449 |
| 41 | 1 | 0 |  | 4.206191 | -3.604670 | -1.625939 |
| 42 | 8 | 0 |  | -2.108610 | -1.126451 | 2.787991 |
| 43 | 1 | 0 |  | -1.358060 | -0.528511 | 2.981571 |
| 44 | 6 | 0 |  | -5.034630 | -2.300641 | -0.974809 |
| 45 | 1 | 0 |  | -4.641490 | -3.156391 | -1.538069 |
| 46 | 1 | 0 |  | -5.692480 | -2.691881 | -0.194139 |
| 47 | 1 | 0 |  | -5.636520 | -1.705231 | -1.668239 |
| **Conformer f-1** | | |  |  |  |  |
| Center | Atomic | Atomic |  | Coordinates (Angstroms) | | |
| Number | Number | Type |  | X | Y | Z |
| 1 | 6 | 0 |  | -4.372939 | -1.622920 | -0.155440 |
| 2 | 1 | 0 |  | -5.041059 | -2.477260 | -0.110340 |
| 3 | 6 | 0 |  | -4.679919 | -0.520570 | -0.968550 |
| 4 | 6 | 0 |  | -3.818999 | 0.600860 | -1.020710 |
| 5 | 1 | 0 |  | -4.070319 | 1.458880 | -1.636780 |
| 6 | 6 | 0 |  | -2.655979 | 0.577031 | -0.272080 |
| 7 | 6 | 0 |  | -2.340219 | -0.534649 | 0.520580 |
| 8 | 6 | 0 |  | -3.199669 | -1.643380 | 0.606760 |
| 9 | 6 | 0 |  | -1.613069 | 1.655661 | -0.045790 |
| 10 | 1 | 0 |  | -2.007200 | 2.338111 | 0.719930 |
| 11 | 6 | 0 |  | -0.387789 | 0.899671 | 0.593940 |
| 12 | 6 | 0 |  | -1.051809 | -0.387889 | 1.161690 |
| 13 | 6 | 0 |  | 0.340041 | 1.765711 | 1.640010 |
| 14 | 1 | 0 |  | -0.365700 | 2.009061 | 2.448970 |
| 15 | 6 | 0 |  | 1.562941 | 1.063151 | 2.225800 |
| 16 | 1 | 0 |  | 1.248541 | 0.344951 | 2.993340 |
| 17 | 1 | 0 |  | 2.181060 | 1.817471 | 2.727400 |
| 18 | 6 | 0 |  | 2.381741 | 0.342901 | 1.197420 |
| 19 | 1 | 0 |  | 3.388531 | 0.051751 | 1.491420 |
| 20 | 6 | 0 |  | 1.971891 | 0.043831 | -0.052420 |
| 21 | 6 | 0 |  | 0.615421 | 0.426451 | -0.485400 |
| 22 | 8 | 0 |  | -0.561589 | -1.170979 | 1.978530 |
| 23 | 8 | 0 |  | 0.259791 | 0.334731 | -1.661230 |
| 24 | 8 | 0 |  | -1.308360 | 2.492791 | -1.143090 |
| 25 | 1 | 0 |  | -0.886870 | 1.923131 | -1.812710 |
| 26 | 8 | 0 |  | 0.805250 | 2.977411 | 1.036570 |
| 27 | 1 | 0 |  | 0.190450 | 3.268621 | 0.344420 |
| 28 | 6 | 0 |  | 2.840311 | -0.635319 | -1.101240 |
| 29 | 1 | 0 |  | 3.080901 | 0.099211 | -1.877090 |
| 30 | 1 | 0 |  | 2.221041 | -1.385879 | -1.610300 |
| 31 | 6 | 0 |  | 4.077781 | -1.300099 | -0.561380 |
| 32 | 1 | 0 |  | 3.886991 | -2.168909 | 0.070470 |
| 33 | 6 | 0 |  | 5.362321 | -0.968199 | -0.781100 |
| 34 | 6 | 0 |  | 6.484441 | -1.787528 | -0.186090 |
| 35 | 1 | 0 |  | 7.132331 | -2.194128 | -0.974100 |
| 36 | 1 | 0 |  | 7.125771 | -1.169388 | 0.456430 |
| 37 | 1 | 0 |  | 6.108541 | -2.624348 | 0.409660 |
| 38 | 6 | 0 |  | 5.821181 | 0.202852 | -1.616650 |
| 39 | 1 | 0 |  | 6.426351 | -0.143198 | -2.465080 |
| 40 | 1 | 0 |  | 4.999891 | 0.803461 | -2.011750 |
| 41 | 1 | 0 |  | 6.466071 | 0.864922 | -1.024100 |
| 42 | 8 | 0 |  | -2.902929 | -2.705150 | 1.384750 |
| 43 | 1 | 0 |  | -2.052559 | -2.526709 | 1.836720 |
| 44 | 6 | 0 |  | -5.936479 | -0.531840 | -1.806120 |
| 45 | 1 | 0 |  | -5.692209 | -0.704240 | -2.861620 |
| 46 | 1 | 0 |  | -6.626069 | -1.318830 | -1.490260 |
| 47 | 1 | 0 |  | -6.458959 | 0.428530 | -1.748280 |
| **Conformer f-2** | | |  |  |  |  |
| Center | Atomic | Atomic |  | Coordinates (Angstroms) | | |
| Number | Number | Type |  | X | Y | Z |
| 1 | 6 | 0 |  | 4.436591 | -0.668047 | -0.871950 |
| 2 | 1 | 0 |  | 5.326001 | -0.722537 | -1.491930 |
| 3 | 6 | 0 |  | 4.232552 | -1.591937 | 0.165760 |
| 4 | 6 | 0 |  | 3.079622 | -1.519488 | 0.981120 |
| 5 | 1 | 0 |  | 2.931552 | -2.231278 | 1.787650 |
| 6 | 6 | 0 |  | 2.147301 | -0.527419 | 0.728780 |
| 7 | 6 | 0 |  | 2.345861 | 0.379512 | -0.318970 |
| 8 | 6 | 0 |  | 3.495351 | 0.334152 | -1.128100 |
| 9 | 6 | 0 |  | 0.871881 | -0.187819 | 1.483400 |
| 10 | 1 | 0 |  | 1.130381 | 0.307051 | 2.425590 |
| 11 | 6 | 0 |  | 0.143271 | 0.891100 | 0.579090 |
| 12 | 6 | 0 |  | 1.239430 | 1.296341 | -0.451180 |
| 13 | 6 | 0 |  | -0.333270 | 2.112550 | 1.406080 |
| 14 | 1 | 0 |  | 0.547429 | 2.645340 | 1.780860 |
| 15 | 6 | 0 |  | -1.182061 | 3.079289 | 0.572190 |
| 16 | 1 | 0 |  | -0.532551 | 3.652810 | -0.101890 |
| 17 | 1 | 0 |  | -1.655551 | 3.796639 | 1.251960 |
| 18 | 6 | 0 |  | -2.222180 | 2.378029 | -0.246250 |
| 19 | 1 | 0 |  | -3.073281 | 2.973618 | -0.576200 |
| 20 | 6 | 0 |  | -2.171440 | 1.078509 | -0.610300 |
| 21 | 6 | 0 |  | -0.999679 | 0.255450 | -0.241860 |
| 22 | 8 | 0 |  | 1.172590 | 2.233121 | -1.252210 |
| 23 | 8 | 0 |  | -0.894878 | -0.911350 | -0.621100 |
| 24 | 8 | 0 |  | 0.097382 | -1.302790 | 1.869110 |
| 25 | 1 | 0 |  | -0.209498 | -1.729360 | 1.050510 |
| 26 | 8 | 0 |  | -1.015580 | 1.702449 | 2.591250 |
| 27 | 1 | 0 |  | -1.697530 | 1.050299 | 2.376740 |
| 28 | 6 | 0 |  | -3.276279 | 0.401908 | -1.401850 |
| 29 | 1 | 0 |  | -2.831019 | -0.266032 | -2.141840 |
| 30 | 1 | 0 |  | -3.823460 | 1.178608 | -1.951980 |
| 31 | 6 | 0 |  | -4.245759 | -0.342702 | -0.509250 |
| 32 | 1 | 0 |  | -4.789029 | 0.295977 | 0.189230 |
| 33 | 6 | 0 |  | -4.512148 | -1.660003 | -0.481940 |
| 34 | 6 | 0 |  | -5.540118 | -2.214953 | 0.477080 |
| 35 | 1 | 0 |  | -6.339027 | -2.738434 | -0.064940 |
| 36 | 1 | 0 |  | -5.086287 | -2.951613 | 1.153220 |
| 37 | 1 | 0 |  | -5.999448 | -1.430404 | 1.085440 |
| 38 | 6 | 0 |  | -3.856997 | -2.693422 | -1.364750 |
| 39 | 1 | 0 |  | -4.599167 | -3.156283 | -2.029200 |
| 40 | 1 | 0 |  | -3.047958 | -2.290122 | -1.974030 |
| 41 | 1 | 0 |  | -3.437517 | -3.502182 | -0.752800 |
| 42 | 8 | 0 |  | 3.685990 | 1.218702 | -2.129170 |
| 43 | 1 | 0 |  | 2.921480 | 1.831082 | -2.146170 |
| 44 | 6 | 0 |  | 5.246413 | -2.684767 | 0.408000 |
| 45 | 1 | 0 |  | 4.864373 | -3.649047 | 0.050700 |
| 46 | 1 | 0 |  | 6.187533 | -2.485426 | -0.110840 |
| 47 | 1 | 0 |  | 5.458493 | -2.797457 | 1.476190 |
| **Conformer f-3** | | |  |  |  |  |
| Center | Atomic | Atomic |  | Coordinates (Angstroms) | | |
| Number | Number | Type |  | X | Y | Z |
| 1 | 6 | 0 |  | -4.130130 | -1.867401 | 0.193940 |
| 2 | 1 | 0 |  | -4.703399 | -2.770531 | 0.378800 |
| 3 | 6 | 0 |  | -4.585130 | -0.911141 | -0.728080 |
| 4 | 6 | 0 |  | -3.849730 | 0.274029 | -0.962420 |
| 5 | 1 | 0 |  | -4.216160 | 1.018929 | -1.662150 |
| 6 | 6 | 0 |  | -2.658840 | 0.459100 | -0.282400 |
| 7 | 6 | 0 |  | -2.195060 | -0.508230 | 0.618960 |
| 8 | 6 | 0 |  | -2.929320 | -1.676700 | 0.885830 |
| 9 | 6 | 0 |  | -1.724850 | 1.654560 | -0.246010 |
| 10 | 1 | 0 |  | -2.153480 | 2.385950 | 0.452930 |
| 11 | 6 | 0 |  | -0.401440 | 1.108300 | 0.411520 |
| 12 | 6 | 0 |  | -0.902570 | -0.156770 | 1.165040 |
| 13 | 6 | 0 |  | 0.275790 | 2.165960 | 1.304260 |
| 14 | 1 | 0 |  | -0.417490 | 2.440400 | 2.114200 |
| 15 | 6 | 0 |  | 1.587180 | 1.664950 | 1.905420 |
| 16 | 1 | 0 |  | 1.380640 | 1.025490 | 2.772670 |
| 17 | 1 | 0 |  | 2.146280 | 2.533470 | 2.274130 |
| 18 | 6 | 0 |  | 2.430170 | 0.898581 | 0.931700 |
| 19 | 1 | 0 |  | 3.467600 | 0.728151 | 1.213260 |
| 20 | 6 | 0 |  | 2.005250 | 0.415560 | -0.253950 |
| 21 | 6 | 0 |  | 0.599050 | 0.605010 | -0.657100 |
| 22 | 8 | 0 |  | -0.298880 | -0.780090 | 2.041440 |
| 23 | 8 | 0 |  | 0.202250 | 0.327320 | -1.789680 |
| 24 | 8 | 0 |  | -1.555990 | 2.373070 | -1.451280 |
| 25 | 1 | 0 |  | -1.106280 | 1.768210 | -2.069630 |
| 26 | 8 | 0 |  | 0.589200 | 3.330660 | 0.533800 |
| 27 | 1 | 0 |  | -0.084010 | 3.474850 | -0.150150 |
| 28 | 6 | 0 |  | 2.888570 | -0.325039 | -1.246620 |
| 29 | 1 | 0 |  | 2.692910 | 0.103141 | -2.238350 |
| 30 | 1 | 0 |  | 2.539820 | -1.361469 | -1.316270 |
| 31 | 6 | 0 |  | 4.363990 | -0.255259 | -0.960130 |
| 32 | 1 | 0 |  | 4.804360 | 0.735081 | -1.086200 |
| 33 | 6 | 0 |  | 5.187950 | -1.254409 | -0.597770 |
| 34 | 6 | 0 |  | 6.665040 | -1.003519 | -0.399140 |
| 35 | 1 | 0 |  | 6.975550 | -1.263659 | 0.621710 |
| 36 | 1 | 0 |  | 7.262650 | -1.629039 | -1.075710 |
| 37 | 1 | 0 |  | 6.930480 | 0.041931 | -0.581080 |
| 38 | 6 | 0 |  | 4.761001 | -2.683749 | -0.364740 |
| 39 | 1 | 0 |  | 5.046161 | -3.007609 | 0.644800 |
| 40 | 1 | 0 |  | 3.686041 | -2.838529 | -0.473010 |
| 41 | 1 | 0 |  | 5.272941 | -3.358099 | -1.064070 |
| 42 | 8 | 0 |  | -2.489489 | -2.597850 | 1.768540 |
| 43 | 1 | 0 |  | -1.643850 | -2.280630 | 2.147460 |
| 44 | 6 | 0 |  | -5.865770 | -1.155911 | -1.490050 |
| 45 | 1 | 0 |  | -5.645490 | -1.519421 | -2.501600 |
| 46 | 1 | 0 |  | -6.491910 | -1.904381 | -0.997400 |
| 47 | 1 | 0 |  | -6.447000 | -0.234651 | -1.596490 |
| **Conformer f-4** | | |  |  |  |  |
| Center | Atomic | Atomic |  | Coordinates (Angstroms) | | |
| Number | Number | Type |  | X | Y | Z |
| 1 | 6 | 0 |  | 3.587082 | 1.463348 | 0.800110 |
| 2 | 1 | 0 |  | 4.159403 | 2.238367 | 1.300400 |
| 3 | 6 | 0 |  | 3.964972 | 1.008007 | -0.473110 |
| 4 | 6 | 0 |  | 3.230421 | -0.011242 | -1.123020 |
| 5 | 1 | 0 |  | 3.537551 | -0.373442 | -2.099390 |
| 6 | 6 | 0 |  | 2.117701 | -0.532671 | -0.487300 |
| 7 | 6 | 0 |  | 1.729371 | -0.060201 | 0.773580 |
| 8 | 6 | 0 |  | 2.465522 | 0.930678 | 1.445310 |
| 9 | 6 | 0 |  | 1.214060 | -1.687021 | -0.879690 |
| 10 | 1 | 0 |  | 1.721509 | -2.615991 | -0.584850 |
| 11 | 6 | 0 |  | -0.048800 | -1.536400 | 0.048520 |
| 12 | 6 | 0 |  | 0.505181 | -0.684940 | 1.226180 |
| 13 | 6 | 0 |  | -0.630621 | -2.905499 | 0.453000 |
| 14 | 1 | 0 |  | 0.139499 | -3.465510 | 1.005470 |
| 15 | 6 | 0 |  | -1.882871 | -2.775688 | 1.318350 |
| 16 | 1 | 0 |  | -1.597901 | -2.566889 | 2.357170 |
| 17 | 1 | 0 |  | -2.400382 | -3.742468 | 1.315090 |
| 18 | 6 | 0 |  | -2.813630 | -1.697658 | 0.851810 |
| 19 | 1 | 0 |  | -3.821240 | -1.717077 | 1.266570 |
| 20 | 6 | 0 |  | -2.504409 | -0.723558 | -0.028220 |
| 21 | 6 | 0 |  | -1.152109 | -0.678829 | -0.618970 |
| 22 | 8 | 0 |  | -0.016279 | -0.529480 | 2.332590 |
| 23 | 8 | 0 |  | -0.884849 | 0.033071 | -1.586420 |
| 24 | 8 | 0 |  | 0.943760 | -1.852340 | -2.257130 |
| 25 | 1 | 0 |  | 0.418620 | -1.075900 | -2.526030 |
| 26 | 8 | 0 |  | -1.006642 | -3.641449 | -0.715130 |
| 27 | 1 | 0 |  | -0.396681 | -3.449119 | -1.445440 |
| 28 | 6 | 0 |  | -3.489399 | 0.346403 | -0.462750 |
| 29 | 1 | 0 |  | -4.499839 | -0.002296 | -0.212470 |
| 30 | 1 | 0 |  | -3.446269 | 0.452513 | -1.549180 |
| 31 | 6 | 0 |  | -3.237438 | 1.670073 | 0.225260 |
| 32 | 1 | 0 |  | -3.321538 | 1.630183 | 1.312130 |
| 33 | 6 | 0 |  | -2.928287 | 2.854032 | -0.330950 |
| 34 | 6 | 0 |  | -2.727866 | 4.077802 | 0.533210 |
| 35 | 1 | 0 |  | -1.717966 | 4.488811 | 0.401670 |
| 36 | 1 | 0 |  | -3.428955 | 4.875343 | 0.252590 |
| 37 | 1 | 0 |  | -2.870006 | 3.856962 | 1.595000 |
| 38 | 6 | 0 |  | -2.754227 | 3.098092 | -1.809740 |
| 39 | 1 | 0 |  | -1.766816 | 3.534592 | -2.007690 |
| 40 | 1 | 0 |  | -2.837917 | 2.192152 | -2.410910 |
| 41 | 1 | 0 |  | -3.496426 | 3.823573 | -2.169270 |
| 42 | 8 | 0 |  | 2.100382 | 1.371839 | 2.667220 |
| 43 | 1 | 0 |  | 1.302662 | 0.877249 | 2.946680 |
| 44 | 6 | 0 |  | 5.160522 | 1.619106 | -1.164290 |
| 45 | 1 | 0 |  | 4.836263 | 2.329377 | -1.935110 |
| 46 | 1 | 0 |  | 5.800643 | 2.159506 | -0.462150 |
| 47 | 1 | 0 |  | 5.763762 | 0.853936 | -1.663100 |

**Table S6** UV-shift and σ values for ECD spectrum comparisons.

| Configuration | UV-Shift (nm) | σ (eV) |
| --- | --- | --- |
| **a** | -8 | 0.3 |
| **b** | 8 | 0.3 |
| **c** | 6 | 0.3 |
| **d** | 0 | 0.3 |
| **e** | 0 | 0.3 |
| **f** | 6 | 0.3 |
| **g** | 8 | 0.3 |
| **h** | -8 | 0.3 |

Computed ^13^C-NMR chemical shifts of each conformer were first Boltzmann-weighted averaged, and then fitted to experimental values by Ordinary Least Squares (OLS) Linear Regression method in order to remove systematic error that results from the conformational search and random error from experimental conditions. All computed chemical shifts showed high correlations to the experimental values, while configuration **e** had the highest *R*^2^ and *R*^2^_adj_ values and least CMAD and CLAD suggesting that it be the true isomer of compound **1**.

**Table S7** Statistics of Ordinary Least Squares (OLS) Linear Regression of experimental and computed ^13^C-NMR chemical shifts.

| Configuration | CMAD^a^ | CLAD^b^ | *R*^2^ | *R^2^*_adj_ | *RMSE* | *F* | *p* value |
| --- | --- | --- | --- | --- | --- | --- | --- |
| e | 3.21 | 8.59 | 0.9954 | 0.9951 | 4.08 | 3868.57 | < 0.01 |
| f | 3.70 | 9.50 | 0.9935 | 0.9931 | 4.83 | 2750.51 | < 0.01 |

^a^ CMAD = corrected mean absolute deviation, computed as $(1/n)\sum_{i}^{n} |\delta_{\text{calc}}-\delta_{\text{exp}}|$, where $\delta_{\text{calc}}$ and $\delta_{\text{exp}}$ refer to the calculated and experimental chemical shifts. ^b^ CLAD = corrected largest absolute deviation, computed as $\max(|\delta_{\text{calc}}-\delta_{\text{exp}}|)$.

**Table S8** Experimental and computed ^13^C-NMR chemical shifts.

**Configuration e**

| Atom | Experimental | e-1 | e-2 | e-3 | e-4 | Boltzmann | Boltzmann (scaled) |
| --- | --- | --- | --- | --- | --- | --- | --- |
| 1 | 67.7 | 79.9 | 79.9 | 80.1 | 79.7 | 79.9 | 73.4 |
| 2 | 154.7 | 165.1 | 165.0 | 165.5 | 165.2 | 165.2 | 155.2 |
| 3 | 116.8 | 121.6 | 121.6 | 121.5 | 121.4 | 121.5 | 113.3 |
| 4 | 147.4 | 162.1 | 162.1 | 161.8 | 161.8 | 161.9 | 152.1 |
| 5 | 115.6 | 120.7 | 120.5 | 120.6 | 120.3 | 120.5 | 112.4 |
| 6 | 157.2 | 165.7 | 165.8 | 165.5 | 165.5 | 165.6 | 155.6 |
| 7 | 121.7 | 124.2 | 124.2 | 123.9 | 124.2 | 124.1 | 115.8 |
| 8 | 200.1 | 208.4 | 208.0 | 208.0 | 207.5 | 208.0 | 196.2 |
| 9 | 71.8 | 82.3 | 82.5 | 82.6 | 82.9 | 82.6 | 76.0 |
| 10 | 196.9 | 205.1 | 205.5 | 205.3 | 204.8 | 205.3 | 193.6 |
| 11 | 138.4 | 145.0 | 144.8 | 144.6 | 144.7 | 144.7 | 135.6 |
| 12 | 141.9 | 161.5 | 160.0 | 160.0 | 160.7 | 160.3 | 150.5 |
| 13 | 32.4 | 37.5 | 37.5 | 37.5 | 37.8 | 37.5 | 32.8 |
| 14 | **67.4** | 74.7 | 75.0 | 75.0 | 74.9 | 75.0 | **68.7** |
| 15 | 27.3 | 34.2 | 31.5 | 31.2 | 33.7 | 32.1 | 27.6 |
| 16 | 121.3 | 130.1 | 126.8 | 127.0 | 129.0 | 127.6 | 119.2 |
| 17 | 132.2 | 145.2 | 148.7 | 148.9 | 145.7 | 147.9 | 138.6 |
| 18 | 17.5 | 19.3 | 18.7 | 18.6 | 19.2 | 18.8 | 14.9 |
| 19 | 25.5 | 27.9 | 27.9 | 27.9 | 27.7 | 27.9 | 23.5 |
| 20 | 21.7 | 24.7 | 24.6 | 24.8 | 24.7 | 24.7 | 20.5 |

**Configuration f**

| Atom | Experimental | f-1 | f-2 | f-3 | f-4 | Boltzmann | Boltzmann (scaled) |
| --- | --- | --- | --- | --- | --- | --- | --- |
| 1 | 67.7 | 83.8 | 82.2 | 83.7 | 83.7 | 83.7 | 77.2 |
| 2 | 154.7 | 162.5 | 164.2 | 162.5 | 162.5 | 162.5 | 153.5 |
| 3 | 116.8 | 120.5 | 121.9 | 120.6 | 120.6 | 120.6 | 112.9 |
| 4 | 147.4 | 161.8 | 161.8 | 162.1 | 161.8 | 161.9 | 152.9 |
| 5 | 115.6 | 120.5 | 120.7 | 120.8 | 120.8 | 120.7 | 113.0 |
| 6 | 157.2 | 166.0 | 166.1 | 166.1 | 165.9 | 166.0 | 156.8 |
| 7 | 121.7 | 123.3 | 123.2 | 123.0 | 122.9 | 123.1 | 115.3 |
| 8 | 200.1 | 203.3 | 206.7 | 203.2 | 203.0 | 203.3 | 192.9 |
| 9 | 71.8 | 80.2 | 80.4 | 79.8 | 80.1 | 80.0 | 73.7 |
| 10 | 196.9 | 205.9 | 203.8 | 205.6 | 205.7 | 205.7 | 195.2 |
| 11 | 138.4 | 143.1 | 145.5 | 143.1 | 143.2 | 143.2 | 134.8 |
| 12 | 141.9 | 159.3 | 160.5 | 158.7 | 159.6 | 159.1 | 150.2 |
| 13 | 32.4 | 36.0 | 37.3 | 36.1 | 36.3 | 36.2 | 31.2 |
| 14 | 67.4 | 78.4 | 77.1 | 78.2 | 78.7 | 78.4 | 72.1 |
| 15 | 27.3 | 31.4 | 33.9 | 31.1 | 33.9 | 31.9 | 27.1 |
| 16 | 121.3 | 126.9 | 130.0 | 127.2 | 128.7 | 127.5 | 119.6 |
| 17 | 132.2 | 148.9 | 145.4 | 148.6 | 145.4 | 148.0 | 139.4 |
| 18 | 17.5 | 18.7 | 19.3 | 18.6 | 19.0 | 18.7 | 14.4 |
| 19 | 25.5 | 27.9 | 27.9 | 27.9 | 27.6 | 27.8 | 23.2 |
| 20 | 21.7 | 24.7 | 24.7 | 24.7 | 24.6 | 24.7 | 20.1 |

**Table S9** Crystal data and structure refinement for compound **3**.

Identification code 02266

Empirical formula C_16_H_16_O_5_

Formula weight 288.29

Temperature 150(2) K

Wavelength 1.54184 Å

Crystal system Monoclinic

Space group *P*2**_1_**/c

Unit cell dimensions a = 11.24184(14) Å α= 90°.

b = 8.09453(11) Å β= 101.7202(14)°.

c = 16.0237(2) Å γ = 90°.

Volume 1427.72(3) Å^3^

Z 4

Density (calculated) 1.341 Mg/m^3^

Absorption coefficient 0.832 mm^-1^

F(000) 608

Crystal size 0.230 x 0.130 x 0.040 mm^3^

Theta range for data collection 4.016 to 70.560°.

Index ranges -13<=h<=13, -9<=k<=9, -17<=l<=19

Reflections collected 11426

Independent reflections 2701 [R(int) = 0.0569]

Completeness to theta = 67.684° 99.9 %

Absorption correction Semi-empirical from equivalents

Max. and min. transmission 1.00000 and 0.27159

Refinement method Full-matrix least-squares on F^2^

Data / restraints / parameters 2701 / 0 / 199

Goodness-of-fit on F^2^ 1.035

Final R indices [I>2sigma(I)] R1 = 0.0465, wR2 = 0.1268

R indices (all data) R1 = 0.0506, wR2 = 0.1330

Extinction coefficient n/a

Largest diff. peak and hole 0.243 and -0.247 e.Å^-3^

**Cytotoxicity Assay**

Cytotoxicity was assayed with the CCK-8 (Dojindo, Japan) method. 10 Cell lines K562, MCF-7, A549, Huh-7, H1975, Hela, HL7702, HL60, MOLT-4, and DU145 were purchased from Shanghai Cell Bank, Chinese Academy of Sciences. Cells were routinely grown and maintained in RPMI or DMEM media with 10% fetal bovine serum and with 1% penicillin/streptomycin. All cell lines were incubated in a Thermo/ Forma Scientific CO_2_ water-jacketed incubator with 5% CO_2_ in air at 37 °C. A cell viability assay was determined with the CCK-8 (Dojindo, Japan) assay. Cells were seeded at a density of 400−800 cells/well in 384-well plates and treated with various concentrations of compounds or solvent control. After 72 h incubation, CCK-8 reagent was added, and absorbance was measured at 450 nm using an Envision 2104 multilabel reader (PerkinElmer, USA). Dose−response curves were plotted to determine the IC_50_ values using Prism 5.0 (GraphPad Software Inc., USA).

**COX-2 Inhibitory Activity Assay**

The COX-2 enzyme is a well-established target for anti-inflammatory evaluation. Hence, the compounds isolated were tested for COX-2 inhibitory activity using the COX (ovine) inhibitor screening kit, as per the manufacturer’s instructions. The COX-2 enzyme catalyses the biosynthesis of PGH_2_ from arachidonic acid. In this assay, the PGF2a produced by the reduction of PGH_2_ with stannous chloride (SnCl_2_) was measured in this enzyme immunoassay. The stock solutions were prepared in dimethyl sulfoxide, and the final concentration was set as 10 mM for the isolated compounds. The percentage inhibition was calculated by comparison with control incubations. The IC_50_ values of the most active compounds were calculated from concentration – inhibition response curves.
